# Discriminatory punishment undermines the enforcement of group cooperation

**DOI:** 10.1038/s41598-023-33167-2

**Published:** 2023-04-13

**Authors:** Welmer E. Molenmaker, Jörg Gross, Erik W. de Kwaadsteniet, Eric van Dijk, Carsten K. W. de Dreu

**Affiliations:** 1 Institute of Psychology, Leiden University, PO Box 9555, 2300 RB Leiden, The Netherlands; 2 Institute of Psychology, Zurich University, Zurich, Switzerland; 3 Center for Experimental Economics and Political Decision Making, University of Amsterdam, Amsterdam, The Netherlands

**Keywords:** Human behaviour, Psychology and behaviour, Social anthropology, Environmental economics

## Abstract

Peer punishment can help groups to establish collectively beneficial public goods. However, when humans condition punishment on other factors than poor contribution, punishment can become ineffective and group cooperation deteriorates. Here we show that this happens in pluriform groups where members have different socio-demographic characteristics. In our public good provision experiment, participants were confronted with a public good from which all group members benefitted equally, and in-between rounds they could punish each other. Groups were uniform (members shared the same academic background) or pluriform (half the members shared the same academic background, and the other half shared another background). We show that punishment effectively enforced cooperation in uniform groups where punishment was conditioned on poor contribution. In pluriform groups, punishment was conditioned on poor contribution too, but also partially on others’ social-demographic characteristics—dissimilar others were punished more than similar others regardless of their contribution. As a result, punishment lost its effectiveness in deterring free-riding and maintaining public good provision. Follow-up experiments indicated that such discriminatory punishment was used to demarcate and reinforce subgroup boundaries. This work reveals that peer punishment fails to enforce cooperation in groups with a pluriform structure, which is rule rather than exception in contemporary societies.

## Introduction

By virtue of cooperation, humans create and maintain public goods that benefit entire groups or societies, like public healthcare and education, social security, or state defence^1,2^. Contributing to the provision of public goods is, however, personally costly and can be exploited by free-riders who benefit from the public good without contributing themselves. The temptation to free-ride on others’ cooperation, alongside the expectation that others may free-ride, makes public good provision and group cooperation fragile and challenging to maintain^3–5^. Indeed, both laboratory experiments and field studies robustly showed that free-riding incentives jeopardize cooperation and that groups have difficulty to sustain public goods over time^6–10^.

To tackle this problem of free-riding, humans can enforce group cooperation by punishing those who refuse to contribute. Groups with rather than without such possibilities to punish each other indeed mitigate free-riding and maintain high levels of group cooperation^6,7,11–15^. The reason is two-fold. First, as punishment is costly to receive, punishing free-riders can effectively reduce the gross benefit of free-riding and already the threat of being punished can lessen the temptation to free-ride^12,15,16^. Second, especially when punishment is costly to impose, punishing free-riding sends a signal to the free-riders about what is and what is not desired behaviour, and this can foster a norm of cooperation that helps towards creating and maintaining public goods^11,17,18^.

For such enforcement of group cooperation to work effectively, humans need to direct their costly punishment indiscriminately and exclusively at free-riders. Whenever humans punish some free-riders more than others, or altogether condition punishment on other factors than poor contribution, peer punishment may lose its effectiveness to deter free-riding^14,19,20^. We conjecture this to happen when group members differ along demographic, cultural, or ideological lines. The reason is that people tend to trust and cooperate well with similar others, whereas they distrust more and cooperate less with dissimilar others^21–25^. Indeed, compared to the uniform groups typically studied in public good provision experiments, groups with more pluriform structures are marked by lower social cohesion and higher propensity for internal conflicts^18,26,27^.

There are at least two accounts of why peer punishment may lose its effectiveness or even become counter-productive in pluriform groups. First, individuals may hold ‘double standards’ for people with different socio-demographic backgrounds, and respond with more leniency to free-riding by similar others than by dissimilar others^28–32^. Second, individuals may adopt an ‘us versus them’ attitude^33^, and punishment opportunities may be used to further demarcate and reinforce subgroup boundaries between the similar ‘us’ and the dissimilar ‘them’ within the larger group, and, ultimately, improve the wealth and/or status of similar others relative to dissimilar others^34,35^. Alone and in combination, these processes would produce subgroup-based discrimination in punishment, with dissimilar others being punished more than similar others. Such discriminatory punishment, in turn, may undermine people’s responsiveness to punishment, or even worse, their overall willingness to cooperate. Accordingly, peer punishment would thwart rather than facilitate group cooperation, and hamper the creation and maintenance of collectively beneficial public goods.

Here, we examined these possibilities in a series of public good provision experiments with both repeated interactions (Experiment 1) and one-off encounters (Experiments 2 and 3). To operationalize the different group structures, we created uniform groups in which all participants shared the same academic background (i.e., psychology or pedagogy) and pluriform groups in which half the members shared the same academic background (e.g., psychology) and the other half shared another academic background (e.g., pedagogy). Throughout the experiments, individual group members were identified to each other with an arbitrary number and, importantly, their academic background (see “Methods” section and Supplementary Information). Participants could impose punishments (at a self-to-other cost ratio of 1:3^7^) on any other member of their own group (Experiment 1) or on members of other pluriform and uniform groups (Experiments 2 and 3). This setup allowed us to see who punishes whom and why, and with what consequences for public good provision.

## Methods summary and results

### Experiment 1

In Experiment 1, participants (*N* = 144) were faced with a fully incentivized, multi-round public goods game (PGG) in groups of four (*k* = 36 groups; 18 pluriform groups and 18 uniform groups). In the PGG, participants received an endowment of 20 monetary units (MU) that they could either keep to themselves or contribute to a group account (i.e., a binary decision). Contributions were multiplied by 1.6 and divided equally among the four group members. Because contributing the endowment of 20 MU resulted in a group return of 32 MU (20 × 1.6) and an individual return of 8 MU (32/4), contributing was an act of cooperation. Keeping the endowment to oneself, by contrast, was an act of free-riding, because on top of the kept 20 MU, one would then still receive a return of 8 MU from each contributing group member. Keeping the endowment, therefore, was always in the material self-interest of any participant. Group members made their contribution decisions simultaneously and were subsequently informed about each other’s choices. To allow cooperation to evolve over time, we repeated this PGG in two blocks of 20 rounds each, while keeping the group composition constant^14,16^.

In one of two blocks (order counterbalanced across groups), participants were given the opportunity to punish their fellow group members. Specifically, at the end of every round, participants had 15 additional MU at their disposal, which they could employ to assign up to five decrement points (DP) to individual group members^7,8,14,16^. Each DP assigned reduced the final earnings of the punished group member by three MU and cost the punishing group member one MU. After participants had made their punishment decisions, they were only informed about the total number of DP each group member received and not about who had imposed these DP. This was done to exclude the possibility of both direct retaliation^14,19,36^ and reputation formation through punishment^34,37,38^.

In public good games, it may not always be clear whether non-contributors are intentionally free-riding^39–42^. Consider, for instance, situations in which people are not able to contribute due to a lack of resources rather than a reluctance to cooperate, which is not necessarily known to others. Accordingly, in 15% of the rounds within each block (i.e., 3 rounds, randomly selected by the computer for each participant), participants received no endowment and, hence, could not contribute to the group account (see Supplementary Information). Who did not have an endowment on any given round was, however, strictly private and not shared. We note that because of this design feature, participants in both the pluriform and the uniform groups could attribute another group member’s non-contribution to selfish free-riding or, alternatively, to the exogenously created inability to contribute. Whereas this induced noise about others’ intentions may demotivate participants from punishing non-contributors, and give them the benefit of the doubt, we expected—akin to the ‘double standards’ people may hold in the pluriform groups—that such more benign attributions would be more likely in case similar rather than dissimilar others did not contribute to the public good.

### Cooperation dynamics

**Figure 1:**
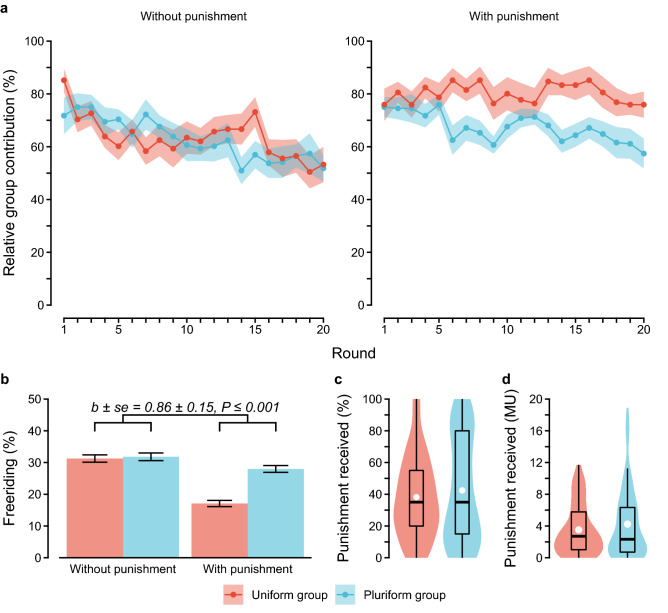
Effectiveness of punishment. (**a**) Mean relative group contributions over rounds as a function of punishment and group structure (100% implies that all members with an endowment in the group contributed to the public good). Bands around the mean indicate ± *se*. (**b**) Mean (± *se*) frequency of free-riding as a function of punishment and group structure. (**c**) Violin plot of the average frequency of receiving punishment and a (**d**) violin plot of the average costs of receiving punishment (per round), as a function of group structure. White dots indicate the means.

Consistent with prior research^7,9,10^, we found that group cooperation deteriorated when punishment was absent. We observed this pattern in both the pluriform and the uniform groups (Fig. 1a). Also consistent with prior research^6,7,12,13^, peer punishment enforced group cooperation in the uniform groups. Averaged across the twenty rounds, the mean relative contribution of uniform groups (i.e., when accounting for whether or not group members were endowed) was 16.62 percentage points higher with than without punishment (80.09% versus 63.47%). This difference reached 22.69 percentage points in the final round. In the pluriform groups, by contrast, punishment hardly changed average group cooperation. The mean relative contribution of pluriform groups differed only by 4.47 percentage points when punishment was present rather than absent (67.18% versus 62.71%).

A mixed-effects regression modelling the total group contribution as a function of punishment (0 = absent, 1 = present) and group structure (0 = uniform, 1 = pluriform) confirmed these observations (punishment × group structure interaction; *b* ± *se* =  − 8.28 ± 1.87, *P* ≤ 0.001; Table S1, column 1). Likewise, free-riding (i.e., when a participant was endowed but did not contribute) was as frequent in the pluriform groups as in the uniform groups when punishment was absent. However, punishment reduced free-riding in the uniform groups, but less so in the pluriform groups (Fig. 1b; mixed-effects logistic regression; Table S2). Consequently, especially when punishment was possible, pluriform groups ended up less wealthy than uniform groups (punishment × group structure interaction; mixed-effects regression, *b* ± *se* =  − 8.81 ± 2.88, *P* = 0.002; Table S1, column 2).

### Peer punishment

On average, participants received punishments from others as frequent in pluriform groups as in uniform groups (Fig. 1c; mixed-effects logistic regression, *b* ± *se* = 0.25 ± 0.56, *P* = 0.657, Table S3, column 1). Moreover, the average costs of receiving punishments from others did not differ between the pluriform and the uniform groups (Fig. 1d; mixed-effects Poisson regression, *b* ± *se* = 0.02 ± 0.51, *P* = 0.975; Table S3, column 2). Across both pluriform and uniform groups, participants mainly directed their punishments at non-contributors rather than contributors (mixed-effects logistic regression, *b* ± *se* =  − 1.71 ± 0.08, *P* ≤ 0.001; Table S4, column 1), and they incurred more costs to punish them (mixed-effects poisson regression, *b* ± *se* =  − 1.01 ± 0.04, *P* ≤ 0.001; Table S5, column 1). However, the difference in punishment of non-contributors and contributors was overall smaller in the pluriform compared to the uniform groups, both for the frequency of punishment (group structure × target contributed interaction; mixed-effects logistic regression, *b* ± *se* = 0.93 ± 0.16, *P* ≤ 0.001; Table S4, column 2) and for the expenditure on punishment (group structure × target contributed interaction; mixed-effects Poisson regression, *b* ± *se* = 0.63 ± 0.07, *P* ≤ 0.001; Table S5, column 2). Thus, in pluriform groups, punishment was conditioned less on whether someone was a contributor or non-contributor.

**Figure 2:**
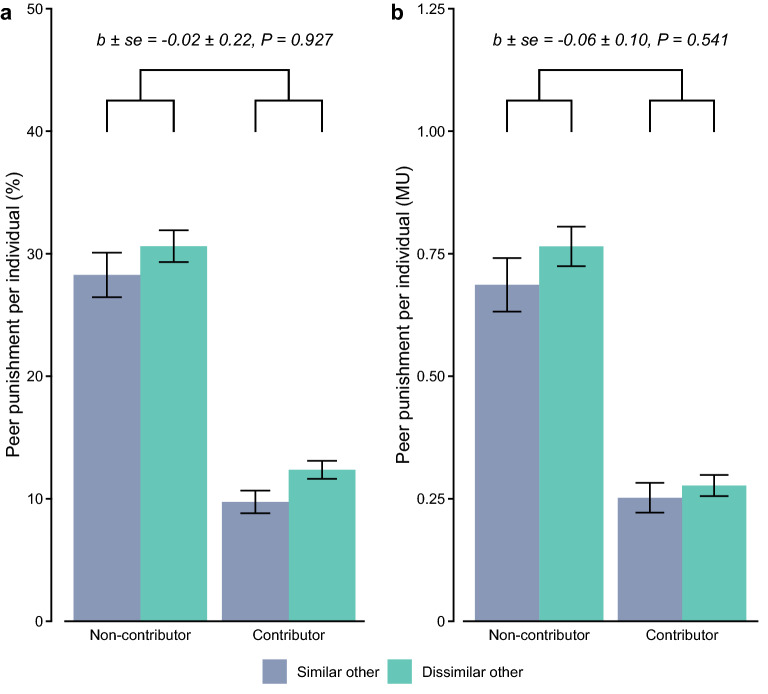
Punishment of non-contributors and contributors in the pluriform group. (**a**) Mean (± *se*) frequency of peer punishment and (**b**) mean (± *se*) expenditure on peer punishment (per individual), as a function of target’s contribution and target’s subgroup.

In the pluriform groups, we further observed that participants, on average, punished dissimilar others more than similar others, both in terms of frequency of punishment (mixed-effects logistic regression, *b* ± *se* = 0.29 ± 0.10, *P* = 0.005; Table S6, column 1) and in terms of expenditure on punishment (mixed-effects poisson regression, *b* ± *se* = 0.11 ± 0.05, *P* = 0.020; Table S6, column 3). Importantly, such discriminatory punishment was unaffected by whether someone had contributed or not (frequency: Fig. 2a, mixed-effects logistic regression; expenditure: Fig. 2b, mixed-effects poisson regression; Table S6, columns 2 and 4). Independently of the target’s behaviour, participants punished a dissimilar other, on average, 2.50 percentage points more often than a similar other (20.21% versus 17.71%) and they spent, on average, 0.048 MU more on punishing a dissimilar other than a similar other (0.487 versus 0.439 MU). Accordingly, in the pluriform groups, punishment was partially conditioned on the academic background of potential targets, irrespective of whether they had contributed to the public good or not.

Results are inconsistent with a ‘double standards’ account that holds that individuals punish free-riding by dissimilar others more than free-riding by similar others^28–32^. Instead, we found that individuals overall punished dissimilar others more than similar others, regardless of their contributing behaviour. Rather, results are consistent with the account that individuals punish dissimilar others more to further demarcate and reinforce subgroup boundaries between ‘us’ versus ‘them’ within the larger group, and, ultimately, improve their relative wealth and status. Participants, indeed, felt more affiliated with others from the same academic background (*M* = 3.82, *SD* = 0.79) compared to others from the other academic background (*M* = 1.33, *SD* = 1.13; mixed-effects regression, *b* ± *se* =  − 2.48 ± 0.09, *P* ≤ 0.001; see Supplementary Information), and the larger this difference was, the more discriminatory punishment towards dissimilar others participants in the pluriform groups exhibited (frequency: mixed-effects logistic regression,* b* ± *se* = 0.19 ± 0.09, *P* = 0.026; expenditure: mixed-effects poisson regression,* b* ± *se* = 0.10 ± 0.04, *P* = 0.010; see Supplementary Information). Notably, we find these results while participants had a second-party punishment perspective (i.e., their earnings depended in large part on the contributing behaviour of their targets of punishment) and because group cooperation deteriorated as a result, discriminatory punishers thus seemed to essentially undermine their own group’s wealth with their punitive behaviour.

### Experiment 2

In Experiment 1, reputation formation through punishment was ruled out by experimental design. Perhaps, however, the emergence of double standards requires a possibility to build reputation through punishment. When individuals punish poor contribution by dissimilar others more than poor contribution by similar others, and thus apply a double standard for free-riding, they may gain a positive reputation among similar others and, by contrast, a deterrent reputation among dissimilar others, as prior work on punishment in intergroup settings suggests^38,43^.

To examine whether reputation concerns alter the nature of discriminatory punishment in pluriform groups, participants in Experiment 2 (*N* = 276) first faced a linear one-shot PGG in pluriform groups of six (“Methods” section and Supplementary Information). Next, they (i.e., the six-person group) served as third parties with individual punishment capacity, overseeing public good provision by another pluriform group of six, composed of three members similar to the participant (i.e., with the same academic background) and three members dissimilar to the participant (i.e., with a different academic background). For all possible contributions in the PGG, participants specified how many decrement points (DP) they would assign to members of this group if they opted for the respective contribution-level (“Methods” section and Supplementary Information). Participants specified their binding punishment strategies once for contributions made by similar others and once for contributions made by dissimilar others (order counterbalanced across participants). Because participants were instructed that each member of the other pluriform group was fully informed about how many DP they—anonymously yet identifiable with an arbitrary number and their academic background—assigned to them, their punishment decisions could reflect on their reputation in the eyes of the similar and dissimilar others.

### Third-party punishment

**Figure 3:**
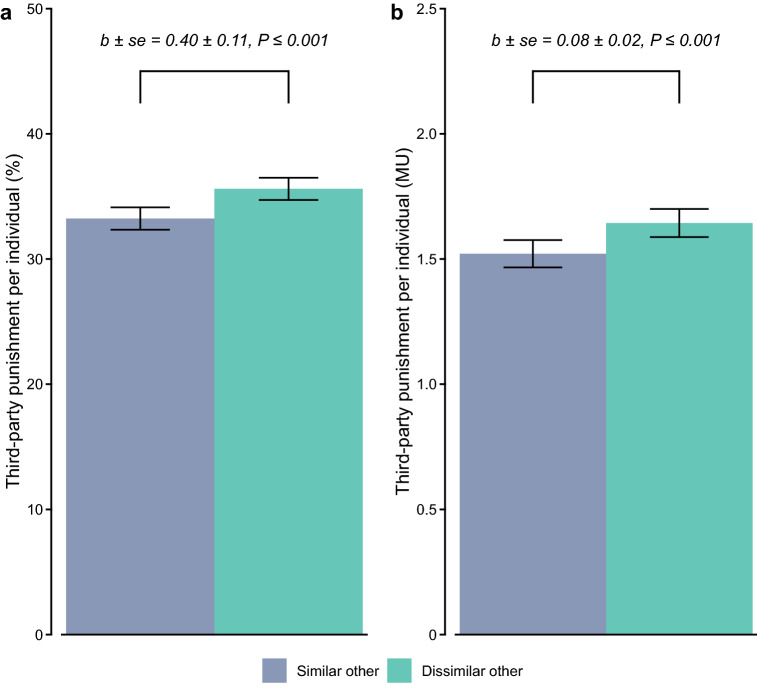
Discriminatory punishment in the pluriform group. (**a**) Mean (± *se*) frequency of third-party punishment and (**b**) mean (± *se*) expenditure on third-party punishment (per individual), as a function of target’s subgroup.

Before participants specified their punishment strategies, they themselves made a contribution decision first. We took their own contributions as a reference point and coded comparatively lower contributions by (dis)similar others as free-riding, and contributions equal or above own contribution as cooperation (see Supplementary Information)^14^. As third parties, participants punished free-riding more than cooperation (mixed-effects logistic regression, *b* ± *se* = 1.615 ± 0.22, *P* ≤ 0.001; Table S7, column 1), and they also incurred more costs to punish free-riding than to punish cooperation (mixed-effects Poisson regression, *b* ± *se* = 0.147 ± 0.04, *P* ≤ 0.001; Table S7, column 3). As in Experiment 1, participants punished dissimilar rather than similar others more frequently (Fig. 3a; mixed-effects logistic regression; Table S7, column 1) and they incurred more costs to punish them (Fig. 3b; mixed-effects Poisson regression; Table S7, column 3). On average, participants punished a dissimilar other 2.38 percentage points more often than a similar other (35.61% versus 33.23%), and they spent, on average, 0.123 MU more on punishing a dissimilar other than a similar other (1.644 versus 1.521 MU). Crucially, and complementing Experiment 1, this differential treatment of dissimilar and similar others was again unaffected by, nor dependent on whether the person was free-riding or cooperating (target’s subgroup × target contributed interaction; frequency: mixed-effects logistic regression, *b* ± *se* =  − 0.07 ± 0.21, *P* = 0.743; expenditure: mixed-effects poisson regression, *b* ± *se* =  − 0.02 ± 0.05, *P* = 0.621; Table S7, columns 2 and 4) (for extensions and robustness checks, see “Methods” section and Supplementary Information; Table S7).

### Experiment 3

The observed patterns of discriminatory punishment in Experiment 2 re-affirm the possibility that people use punishment to further demarcate and reinforce subgroup boundaries between similar and dissimilar others within the larger group. Experiment 3 was designed to further understand when and how punishment opportunities are used to discriminate between the similar ‘us’ and the dissimilar ‘them’. Participants (*N* = 179) were again third parties and could, this time, not only punish individuals in a pluriform group (similar to Experiment 2), but also individuals in two uniform groups—one composed of members similar to the participant, and one composed of members dissimilar to the participant (see “Methods” section and Supplementary Information). Participants specified their binding punishment strategies for all possible contributions by similar or dissimilar others within each of the three group compositions (pluriform; uniform similar; uniform dissimilar), resulting in four separate punishment strategies.

If punishment is used to demarcate and reinforce subgroup boundaries, and create a comparative wealth advantage for similar others, we should see differential punishment of dissimilar versus similar others. However, predominantly when third parties oversee a pluriform group in which intergroup comparisons are the most salient, rather than when they oversee uniform groups, regardless of whether these uniform groups consist of members with whom they share background characteristics or not. If, in contrast, discriminatory punishment is driven by other factors, like a general distaste for dissimilar others, we should see more punishment of dissimilar others, regardless of whether groups are uniform or pluriform^38^.

### Third-party punishment across uniform versus pluriform groups

**Figure 4:**
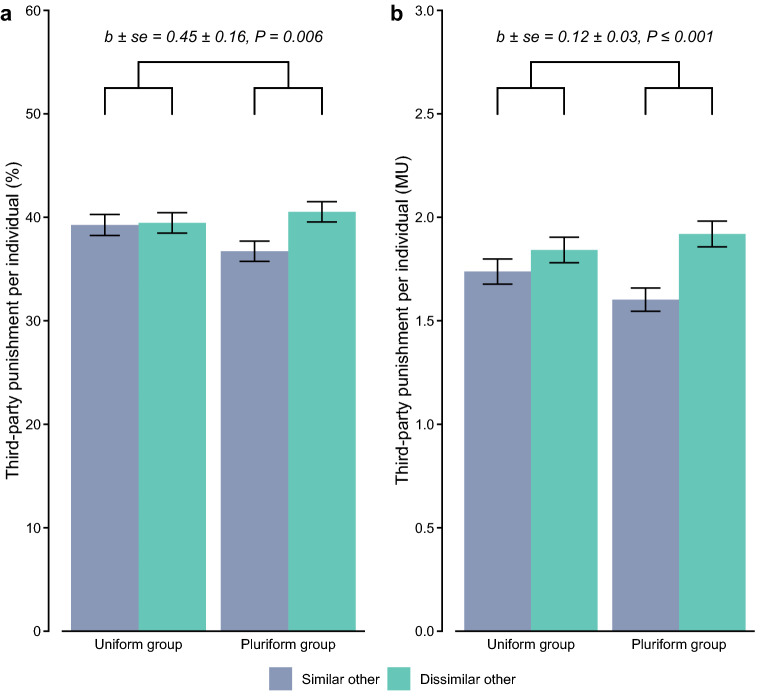
Discriminatory punishment in the pluriform and uniform groups. (**a**) Mean (± *se*) frequency of third-party punishment and (**b**) mean (± *se*) expenditure on third-party punishment (per individual), as a function of group structure and target’s subgroup.

Replicating our earlier findings, participants punished dissimilar rather than similar others in the pluriform group more frequently (40.53% versus 36.72%; mixed-effects logistic regression, *b* ± *se* = 0.52 ± 0.12, *P* ≤ 0.001; Table S8, column 4) and they incurred more costs to punish them (1.919 versus 1.602 MU; mixed-effects Poisson regression, *b* ± *se* = 0.18 ± 0.02, *P* ≤ 0.001; Table S9, column 4). In line with the demarcation account, when similar and dissimilar others were each in separate uniform groups, third parties were less inclined to condition punishment on others’ academic background (target’s subgroup × group structure interaction; frequency: Fig. 4a; mixed-effects logistic regression; Table S8, column 2; expenditure: Fig. 4b; mixed-effects Poisson regression; Table S9, column 2). In fact, dissimilar others in the uniform group were punished as frequently as similar others in the uniform group (39.46% versus 39.26%; mixed-effects logistic regression, *b* ± *se* = 0.03 ± 0.12, *P* = 0.814; Table S8, column 3), but they did incur more costs on punishing them (1.842 versus 1.738 MU; mixed-effects Poisson regression, *b* ± *se* = 0.06 ± 0.02, *P* = 0.014; Table S9, column 3). Thus, these results again speak to the possibility that punishment was used to further demarcate and reinforce the subgroup boundaries between the similar ‘us’ and dissimilar ‘them’ within the pluriform group, but not to discriminate between separate uniform groups of similar and dissimilar others.

## Discussion

Here, we provide evidence that peer punishment fails to enforce cooperation in pluriform groups. The effectiveness of peer punishment has mainly been investigated in uniform groups, and it was found that costly punishment can indeed successfully mitigate free-riding and allows groups to maintain high levels of public good provision^6,7,11–13,15,44^. We replicated these positive effects of punishment, even in a setting in which participants could not perfectly tell whether poor contribution reflected intentional free-riding or an inability to contribute. These positive effects emerged, however, only in our uniform groups in which members shared the same socio-demographic background. In our pluriform groups, by contrast, members differed in their backgrounds, and we observed that dissimilar others were punished more than similar others. Of note is that participants were unable to identify which group members punished and they could, therefore, not tell whether socio-demographics were a decisive factor in the punishment decisions. Importantly, peer punishment nevertheless failed to induce a sustained willingness to cooperate and thus lost its effectiveness in deterring free-riding. As a consequence, group cooperation deteriorated in pluriform groups.

The introduction of a pluriform group structure did not change the overall frequency of and expenditure on punishment. Moreover, costly punishment was still predominantly directed at non-contributors. Yet, across all three experiments, this key boundary condition for effective peer punishment was often violated in pluriform groups, because participants partially conditioned their costly punishment also on others’ socio-demographic characteristics—those with a different academic background were punished more than those with a similar background. Such discriminatory punishment was unaffected by, nor dependent on whether these individuals were free-riding or cooperating and emerged predominantly within pluriform groups, rather than between separate uniform groups of similar and dissimilar others. Although these results resonate with research on punishment in intergroup settings where members from distinct groups interact with each other^30–32,43^, no prior research, to our knowledge, has investigated the emergence, underlying process, and potential consequences of discriminatory punishment in intragroup settings, i.e., in pluriform groups where members with different socio-demographic characteristics share collectively beneficial public goods.

In contemporary societies, group members differing in socio-demographic characteristics is rule rather than exception. Whereas earlier work on punishment and public good provision largely ignored pluriform group structures, our findings suggest that such structures can turn peer punishment into a double-edged sword: On the one hand costly punishment is used to prevent exploitation of collectively beneficial public goods, but on the other hand it is also partially used to further demarcate and reinforce subgroup boundaries between the similar ‘us’ and dissimilar ‘them’ within the pluriform group. In other words, punishment behaviour can be driven by both deterrent and competitive motives^34^. Further demarcating and reinforcing the subgroup boundaries through discriminatory punishment may thus reflect a strategy to, ultimately, improve the relative wealth and/or status of similar others compared to dissimilar others within the pluriform group^34,35^, albeit at the expense of group cooperation and overall wealth (including one’s own).

Our experiments demonstrate that pluriform group structures give rise to discriminatory punishment, and that this, in turn, counteracts the ability of peer punishment to help groups with establishing public goods. Notably, although a substantial proportion of the participants engaged in discriminatory punishment (of dissimilar others), it is also important to highlight that not all participants were discriminatory punishers (for further details and results, see Supplementary Information; Figs. S29, S31, S33). Also noteworthy, however, is that we found these results among psychology and pedagogy students, who form natural subgroups within an overarching social collective (i.e., their faculty) but were in fact not much different from each other, nor had a history of conflict or competition. It stands to reason that in pluriform groups with larger subgroup-differences and/or a history of between-subgroup conflict or competition, stronger discriminatory punishment and deterioration of group cooperation may be observed^45–47^. Moreover, we cannot exclude the possibility that similar detrimental effects may emerge for other cooperation-enforcing institutions as well, like reward^16,48^, gossip^49^, or centralized authorities^44,50,51^ (i.e., leaders, governments), because these institutions can be prone to subgroup-based discrimination too. Thus, our findings challenge the understanding of the institutions that effectively enforce group cooperation, as people may use them to demarcate and reinforce subgroup boundaries within the larger group, thereby hampering the creation and maintenance of collectively beneficial public goods.

## Methods

### Research ethics and participants

The experiments were approved by the Psychology Research Ethics Board of Leiden University, all methods were performed in accordance with the relevant guidelines and regulations, and written informed consent was obtained from all the study participants upon arrival in the laboratory. Participants were recruited among first-year students in the study programmes Psychology and Pedagogical Science at Leiden University and offered a participation fee of €6.50 (or two participant credits) for participating in a one-hour experiment on “group decision making”. The experiments did not involve any deception and participants received an additional payment, on top of their participation fee, based on their actual earnings in the experiment.

### Experimental procedures

Participants were seated in individual cubicles, each containing a personal computer that was used to present the instructions and register their decisions. The instructions were phrased in neutral language throughout. The experiments always began by informing participants that they would engage in a group decision-making task together with (fellow) psychology and pedagogy students and an assessment of the extent to which they feel affiliated with other students from each of these study programmes. Experimental instructions and all posed questions (as well as the full models underlying the reported results) are in the Supplementary Information.

In Experiment 1, participants (77 psychology and 67 pedagogy students) were randomly allocated to a uniform or pluriform group of four, and received instructions and comprehension questions that explained the pay-off structure in a round, the random allocation of endowments across rounds, and the additional payment they could earn (e.g., the MU earned in one randomly selected round per block were converted to euros at a rate of 1 MU = €0.80 and paid out in cash). Before the first block of rounds, participants were not yet informed that a second block would follow and participants only received the instructions about assigning decrement points (DP) in the relevant block.

In Experiments 2 and 3, participants were randomly assigned to either a give-some treatment (capturing the problem of providing a public good) or a take-some treatment (capturing the problem of preserving a common resource)^52^. For brevity, we describe the procedure of the give-some treatment here (and the procedure of the take-some treatment only in the Supplementary Information). Note, however, that the reported results collapse across treatments and that treatment had no significant effects in any of our models (see Supplementary Information). The participants in Experiment 2 (147 psychology and 129 pedagogy students) were confronted with a linear one-shot PGG and a subsequent third-party punishment game (TPG) in pluriform groups of six (with three students from each study programme). The participants in Experiment 3 (90 psychology and 89 pedagogy students), by contrast, were confronted with the linear one-shot PGG and the TPG twice: In uniform groups of three and in pluriform groups of six. In both experiments, we posed some questions throughout the experiment to explore how participants perceived the other psychology and pedagogy students in the group(s) (Supplementary Information). The punishment decisions were elicited with the strategy method^52,53^ and each participant’s additional payment was calculated and paid out in cash 2 weeks after data collection. In addition to the money, participants also received a personal feedback sheet that provided complete information about how their additional payment was achieved (Supplementary Information).

In the PGG of Experiments 2 and 3, each group member received an endowment of 100 monetary units (MU; worth €5 in Experiment 2 and €2.50 in Experiment 3), which they could keep for themselves or contribute (in steps of 10 MU) to a group account. Since participants in Experiment 3 performed the PGG twice, they also received the endowment twice. If participants contributed MU, these MU were multiplied by 1.5 and divided equally among the members of their group. Before participants made their contribution decision(s), they first received instructions about the TPG. In the TPG, each group member was given the opportunity to assign up to 10 DP to each member of another group by indicating for all possible contributions in the PGG, how many DP they assign to the psychology versus pedagogy students if they opted for the respective contribution-level. Each assigned DP reduced the final earnings of each punished other by three MU and cost the punisher one MU (from the additional MU for punishment they received, like in Experiment 1). The instructions also informed participants that their group could be punished by members of yet another group (see Supplementary Information).

## Supplementary Information

Supplementary Information.

## Data Availability

The data and analyses codes (in R) are available via the Open Science framework at https://osf.io/x4uq9.
