# Supplementary material for: Discriminatory punishment undermines the enforcement of group cooperation
Source: Sci Rep. 2023 Apr 13;13:6061. doi: 10.1038/s41598-023-33167-2 (PMC10101972; doi:10.1038/s41598-023-33167-2)
Supplement: Supplementary file 1 — Supplementary Information. [file 41598_2023_33167_MOESM1_ESM.pdf]

*Supplementary Information for*

# Discriminatory punishment undermines the enforcement of group cooperation

Welmer E. Molenmaker*, Jörg Gross, Erik W. de Kwaadsteniet,
Eric van Dijk, and Carsten, K. W. de Dreu

*Correspondence author: w.e.molenmaker@fsw.leidenuniv.nl

This file includes:

1. Supplementary Methods
    1.1. Participants
    1.2. Experimental Design, Procedures, Materials, and Instructions
        1.2.1. Experiment 1
        1.2.2. Experiment 2
        1.2.3. Experiment 3
    1.3. Statistical Procedures
2. Supplementary Results
    2.1. Experiment 1
        2.1.1. Extended Results
        2.1.2. Additional and Exploratory Results
    2.2. Experiment 2
        2.2.1. Extended Results
        2.2.2. Additional and Exploratory Results
    2.3. Experiment 3
        2.3.1. Extended Results
        2.3.2. Additional and Exploratory Results
    2.4. Sensitivity Analyses
3. Supplementary References

# 1. Supplementary Methods

## 1.1. Participants

For our experiments, first-year students from the study programmes in Psychology and Pedagogical Science were recruited. There are two reasons why we recruited psychology and pedagogy students. First, both study programmes are part of the Faculty of Social and Behavioural Sciences of Leiden University and are housed in the same building. Psychology and pedagogy students, therefore, are part of the same overarching collective and use the same facilities (e.g., class rooms, study areas, laboratories, library facilities, and restaurant areas). Hence, they are part of a real-life pluriform group, which is exactly the group structure we aim to study. Second, psychology and pedagogy students strongly resemble each other in terms of demographics, personality type, and study interest, which can help to rule out or reduce the problem of hidden variables that may influence the dynamics over and beyond the pluriform group structure. If under these conditions, group composition (i.e., uniform versus pluriform group structure) would nevertheless have an effect on punishment, and group cooperation and wealth, this would provide compelling evidence for our reasoning.

## 1.2. Experimental Design, Procedures, Materials, and Instructions

Here, we document the experimental design, procedures, materials, and instructions of our experiments. Experiment 1 was conducted in English. We shortly describe the experimental design and procedure of this first experiment, and provide screenshots of the original materials and instructions. Experiments 2 and 3 were conducted in Dutch and both consisted of a give-some treatment and a take-some treatment. For Experiments 2 and 3, we therefore provide a comprehensive and detailed description of the experimental design, procedures, and materials.

### 1.2.1. Experiment 1

*Participants and Experimental Design*

Experiment 1 was conducted in the behavioural laboratory, located in the building of the Faculty of Social and Behavioural Sciences of Leiden University. A total of 144 first-year psychology students ($n$ = 76) and pedagogy students ($n$ = 68) from this university participated (124 women, 19 men, and 1 other; $M_{age}$ = 21.16, $SD_{age}$ = 3.56 years). The sample size was determined based on feasibility concerns rather than a priori power calculations (see *Supplementary Results* for a sensitivity analysis). Given the number of first-year students in the study programmes Psychology and Pedagogical Science, and the time available in the laboratory, we aimed to create 20 pluriform groups, 10 uniform groups of psychology students, and 10 uniform groups of pedagogy students (requiring 80 psychology students and 80 pedagogy students).

During recruitment, participants indicated their study programme and based on availability, they were scheduled for an experimental session with either a pluriform group (18 groups), a uniform group of psychology students (10 groups), or a uniform group of pedagogy students (8 groups). In each session, we could run a maximum of two 4-person groups simultaneously. Whether sessions were with pluriform and/or uniform groups was alternated over time.

After completing the experiment, two participants (each in a uniform group) indicated that they were enrolled in another study programme than Psychology or Pedagogical Science. They were recruited at lectures and workgroups of these study programmes and throughout the whole experiment, we addressed them as student in the programme they had indicated during recruitment. Because excluding the data of these participants from our statistical analyses did not alter the pattern of results, we decided to retain their data and the data of their group members.

*Experimental Procedure*

Upon arrival in the laboratory, participants were seated in individual cubicles, each containing a personal computer that was used to present the instructions and register their decisions. The experiment began by informing participants that they would engage in a group decision-making task in which they would interact with fellow students from the study programmes Psychology and Pedagogical Science. We assessed the extent to which participants felt affiliated with other psychology and pedagogy students, and students from the Faculty of Social and Behavioural Sciences in general, on a 6-point Likert scale ranging from 1 (*completely disagree*) to 6 (*completely agree*) (*Figure S1-S3*; items adapted from[1,2]; $\alpha_{own} = 0.86$, $\alpha_{other} = 0.91$, $\alpha_{general} = 0.89$).

Next, participants received some general instructions about the experiment (*Figures S4 and S5*). This was followed by more detailed instructions and comprehension questions about the multi-round public goods game (PGG) they faced in the first block (*Figures S6, S7, S9, and S10*), which was either without punishment (*Figure S8*) or with punishment (*Figures S18, S19, and S20*). After the comprehension questions, the first block started. Each round, participants first made their contribution decision (*Figures S11 and S12*) and then received feedback about the contribution decisions of each group member (*Figure S13*). If applicable for this block, participants made their punishment decisions right after (*Figure S22*) and then received feedback about the punishments that each group member received (*Figure S23*). Finally, participants received an overview of the round (*Figure S14 or S24*) before moving to the next round. After 20 rounds, the first block was finished and we assessed participants beliefs about the frequency of free-riding by the other group members in the first block (*Figure S15*).

Then, participants proceeded to the next block (*Figure S16*) and learned that this second block of interactions was with punishment (*Figures S17-S21*) or without punishment (*Figures S17,*

*S8, and S21*). Each round, and similar to the first block, participants first made their contribution decision (*Figures S11 and S12*) and then received feedback about the contribution decisions of each group member (*Figure S13*). Again, if applicable for this block, participants made their punishment decisions right after (*Figure S22*) and then received feedback about the punishments each group member received (*Figure S23*). Finally, participants received an overview of the round (*Figure S24 or S14*) before moving to the next round. After 20 rounds, the second block was finished and we assessed participants beliefs about the frequency of free-riding by the other group members in the second block (*Figure S15*) and the PGG was thereafter finished (*Figure S16*). Finally, participants completed the social value orientation slider measure (*Figure S25*)[3], and we asked their demographics together with questions probing their experience with behavioural experiments (*Figure S26*).

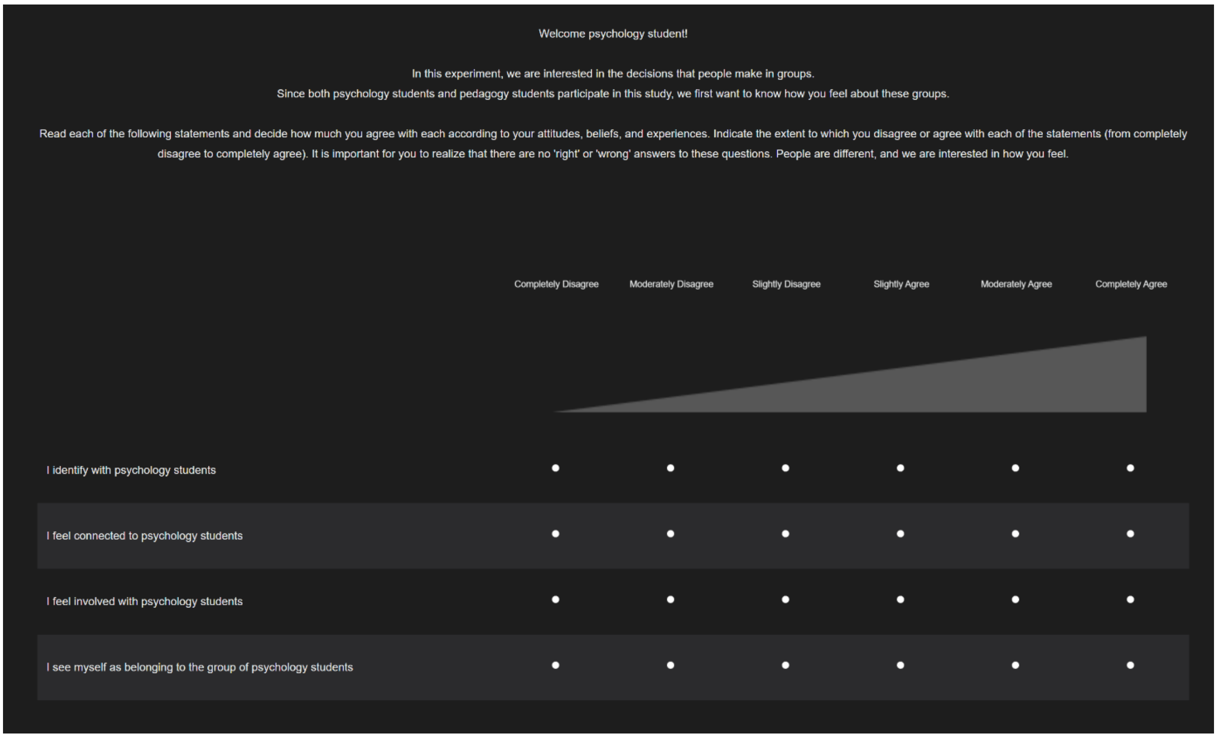


**Figure S1. First assessment of felt affiliation.** Example of psychology student.

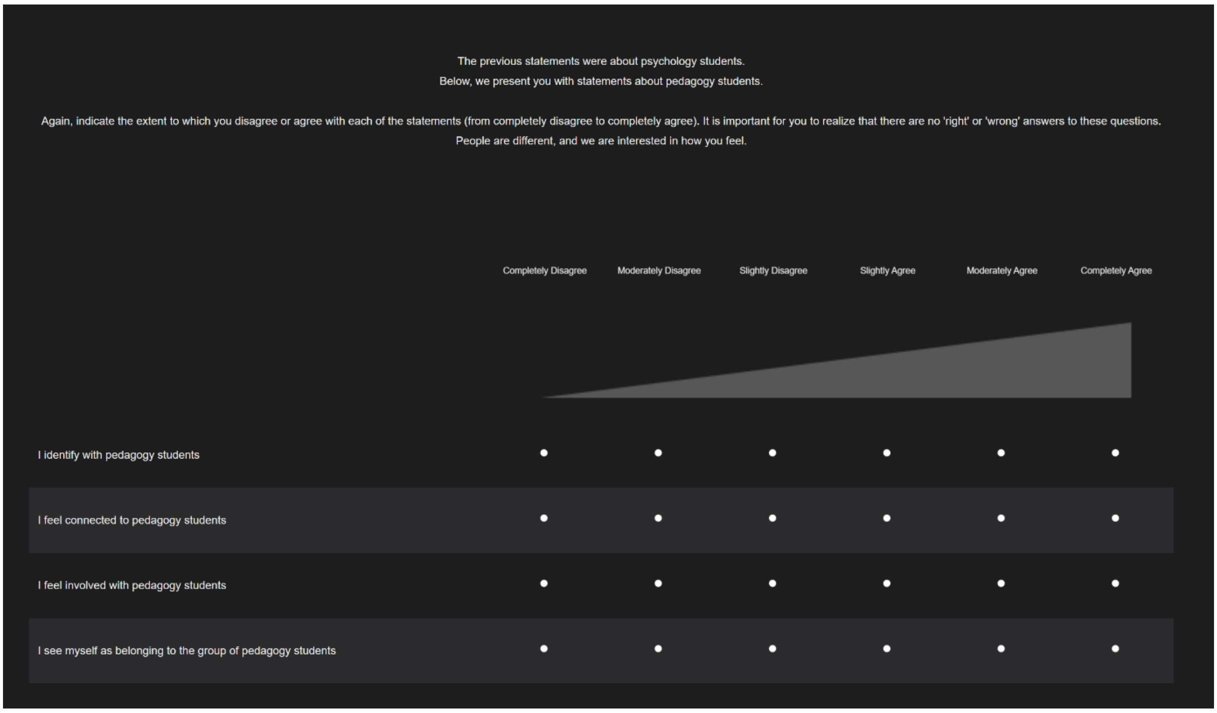


**Figure S2. Second assessment of felt affiliation.** Example of psychology student.

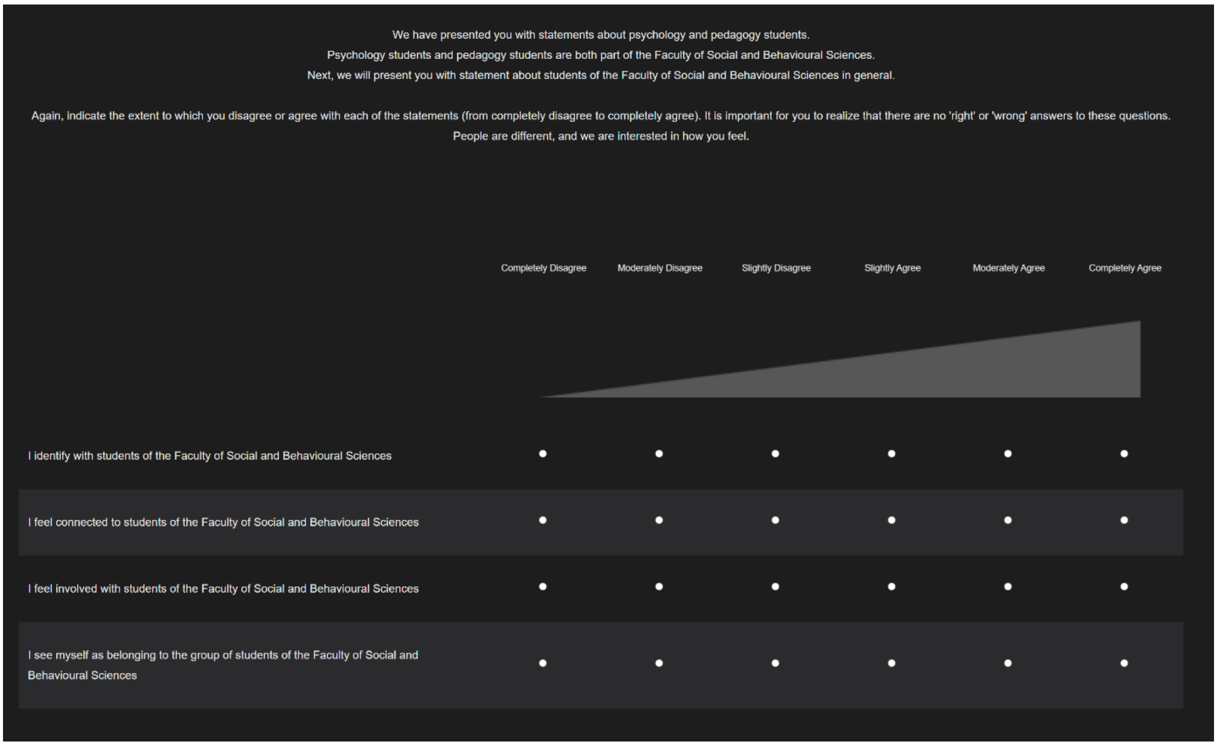


**Figure S3. Third assessment of felt affiliation.** Example of psychology student.

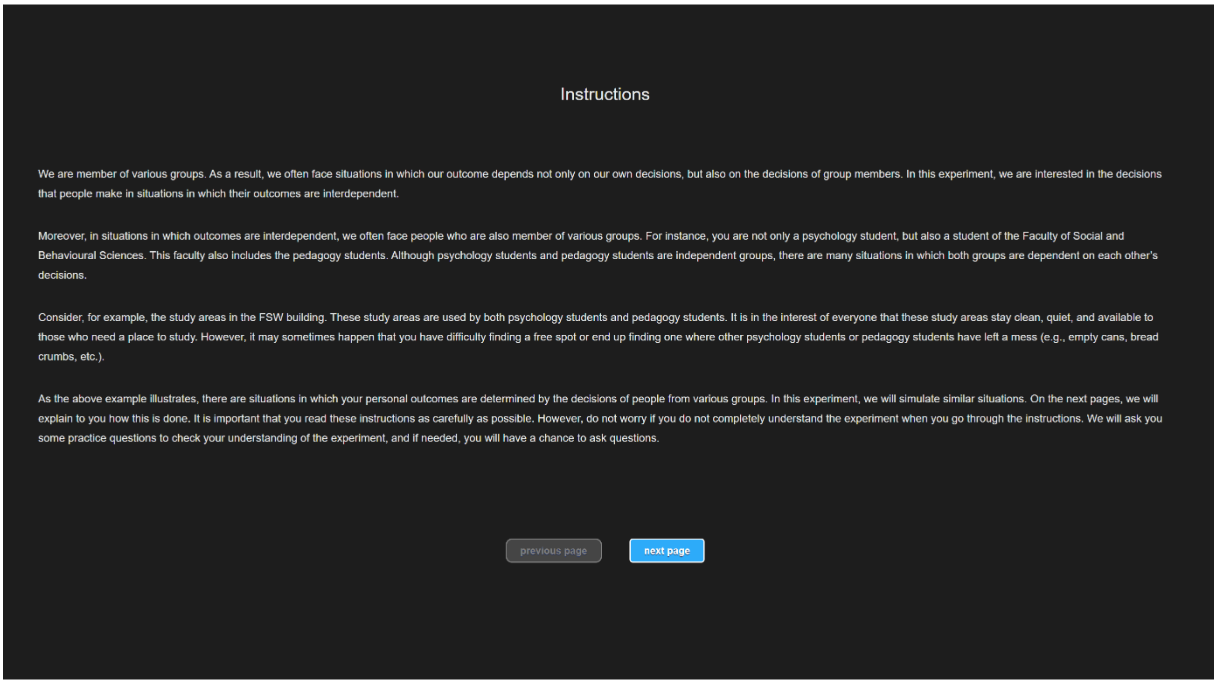


**Figure S4. First instruction page.**

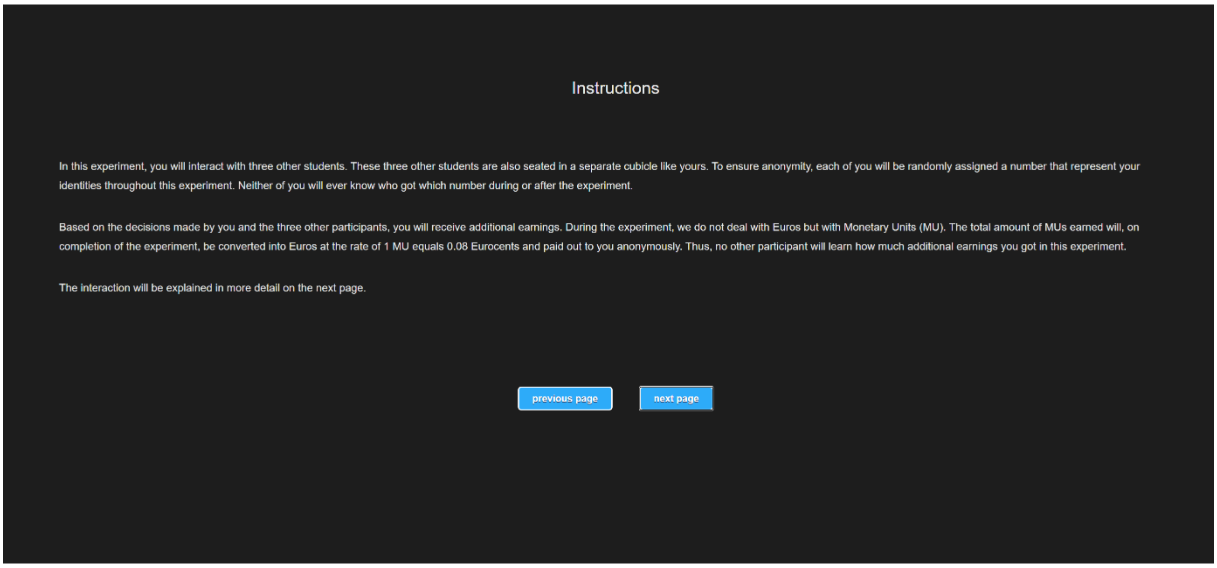


**Figure S5. Second instruction page.**

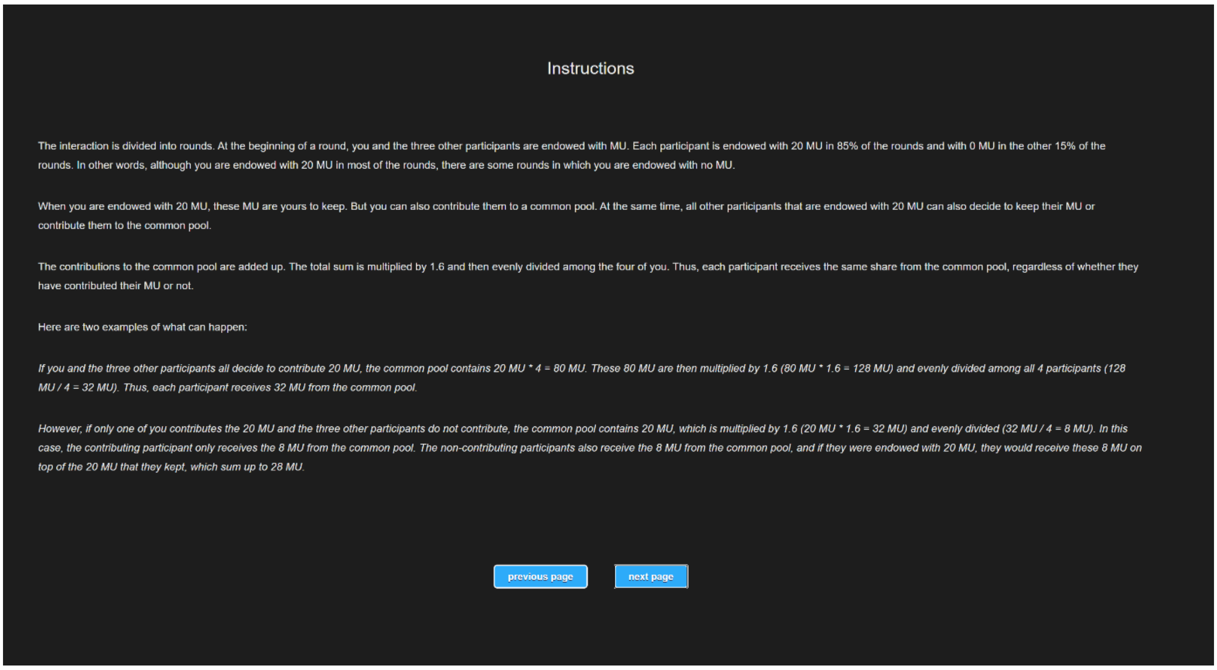


**Figure S6. Third instruction page.**

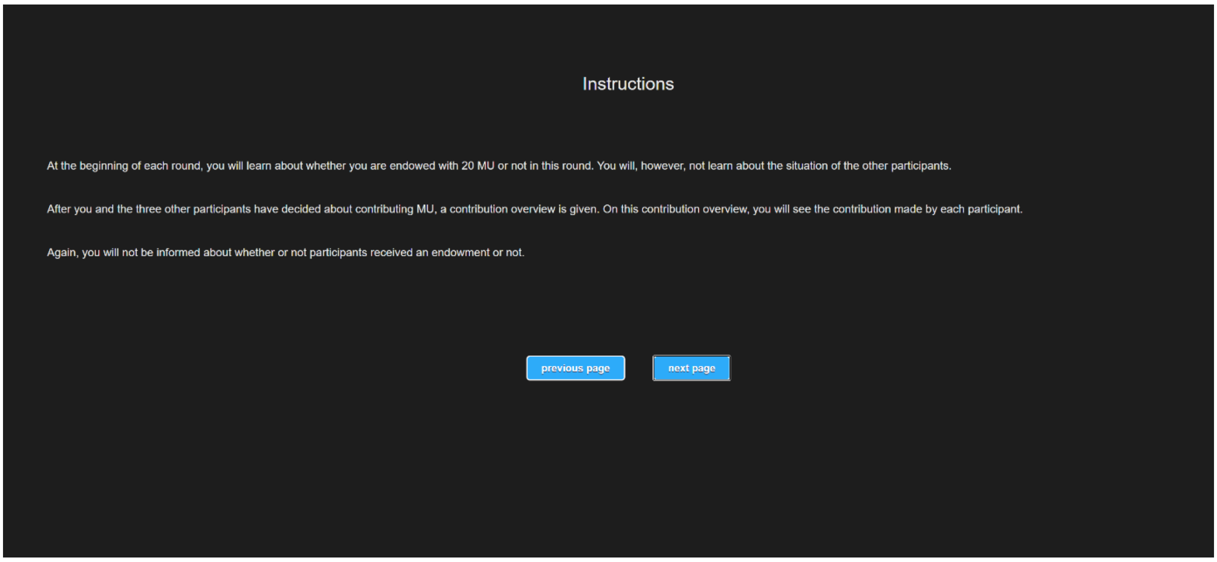


**Figure S7. Fourth instruction page.**

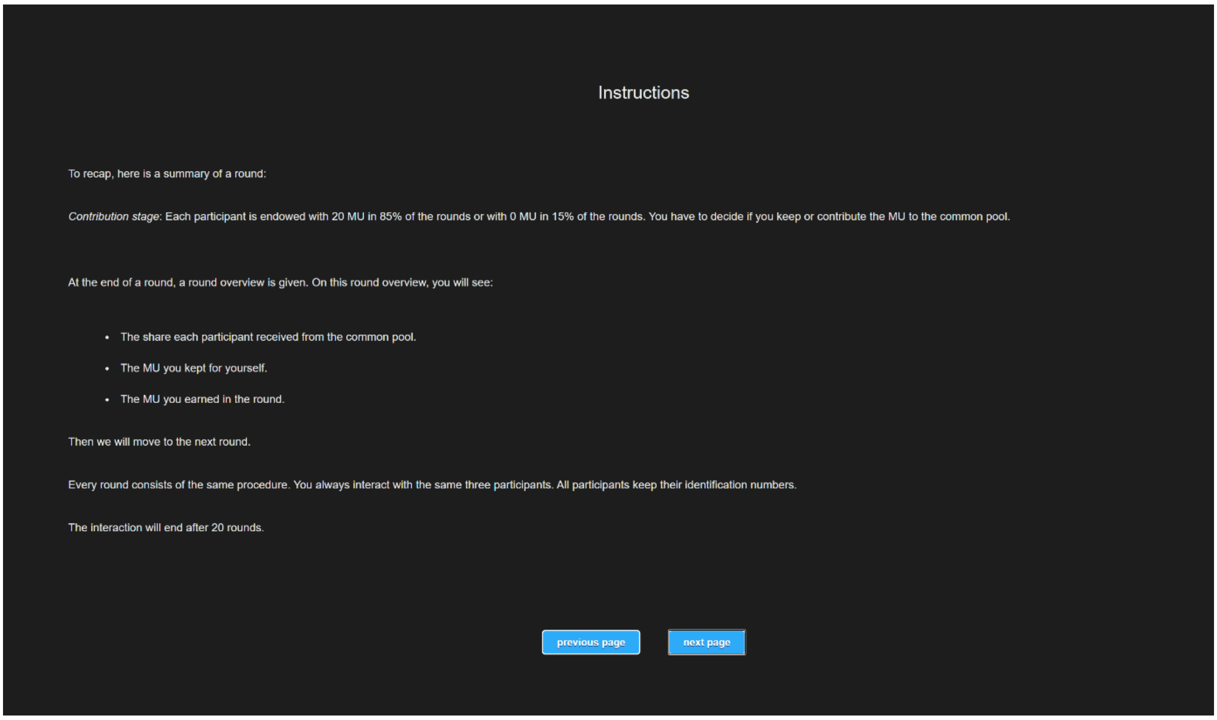


**Figure S8. Recap page.** Without punishment.

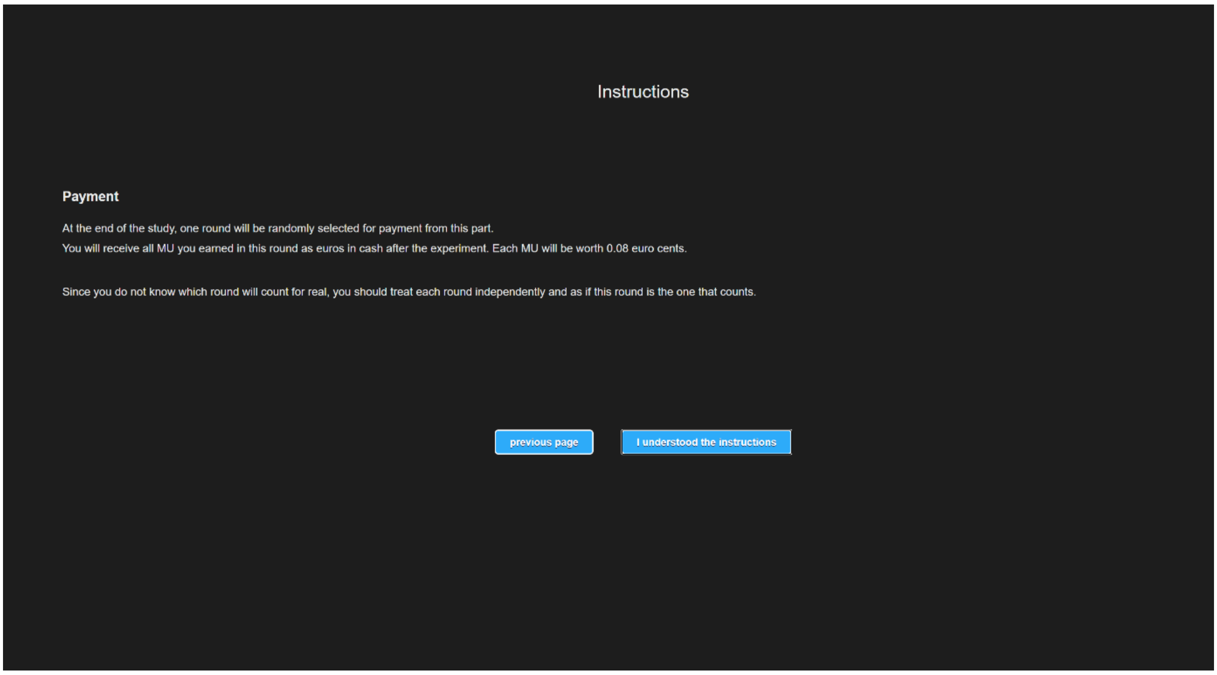


**Figure S9. Payment instruction page.**

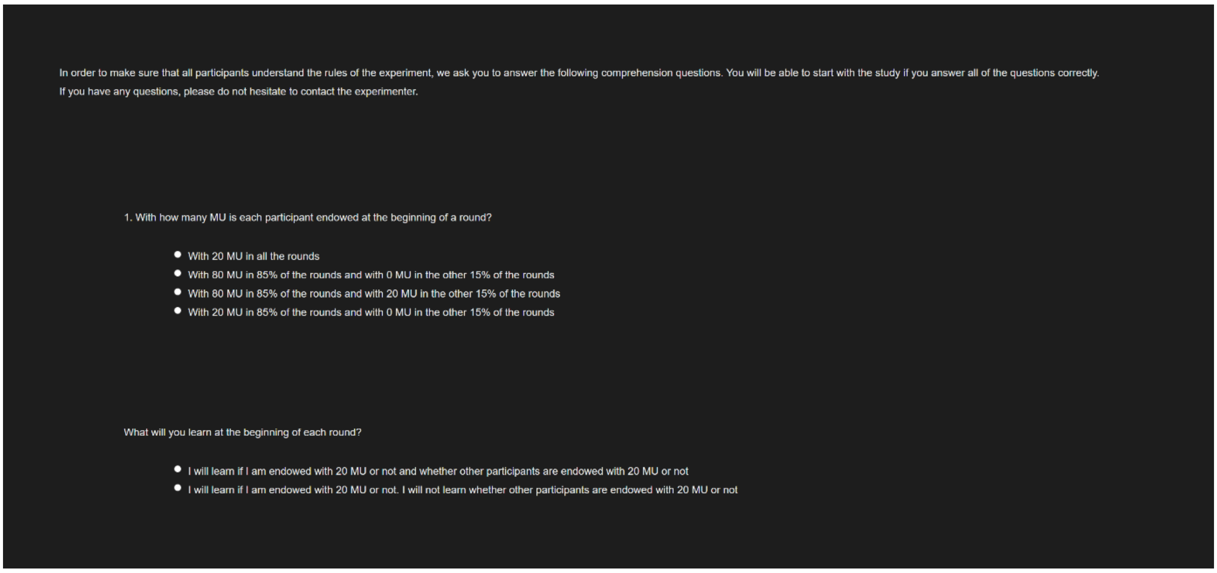


**Figure S10. Comprehension questions.**

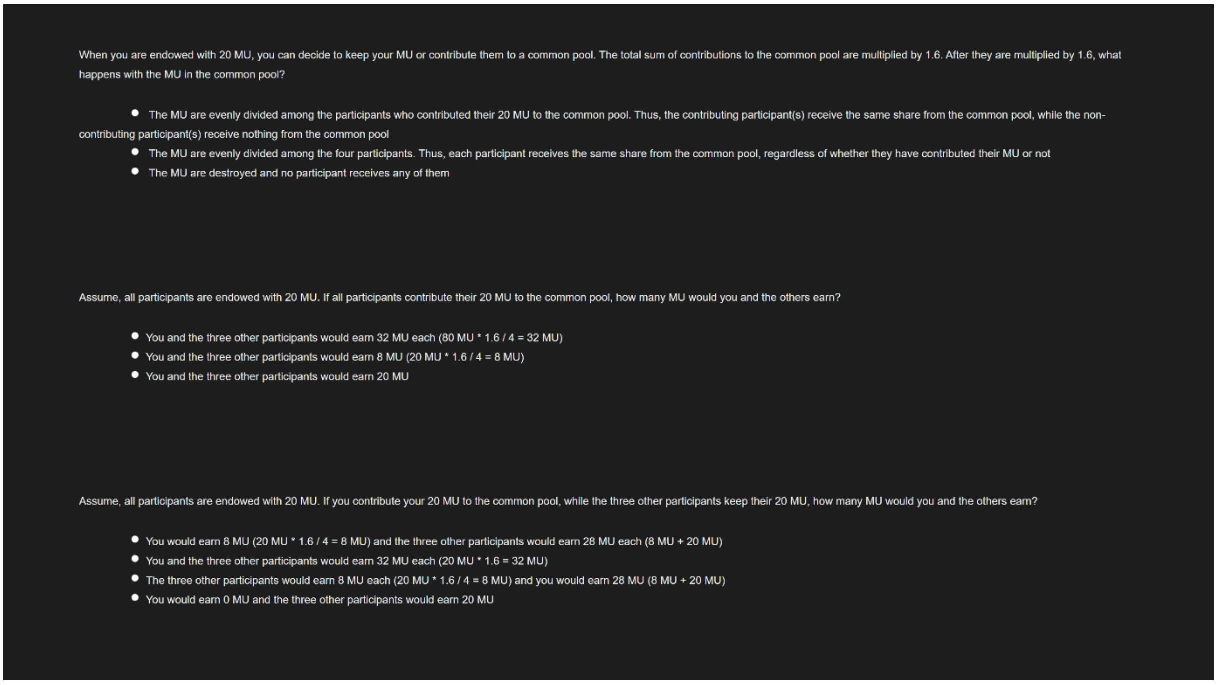

When you are endowed with 20 MU, you can decide to keep your MU or contribute them to a common pool. The total sum of contributions to the common pool are multiplied by 1.6. After they are multiplied by 1.6, what happens with the MU in the common pool?

- The MU are evenly divided among the participants who contributed their 20 MU to the common pool. Thus, the contributing participant(s) receive the same share from the common pool, while the non-contributing participant(s) receive nothing from the common pool
- The MU are evenly divided among the four participants. Thus, each participant receives the same share from the common pool, regardless of whether they have contributed their MU or not
- The MU are destroyed and no participant receives any of them

Assume, all participants are endowed with 20 MU. If all participants contribute their 20 MU to the common pool, how many MU would you and the others earn?

- You and the three other participants would earn 32 MU each (80 MU * 1.6 / 4 = 32 MU)
- You and the three other participants would earn 8 MU (20 MU * 1.6 / 4 = 8 MU)
- You and the three other participants would earn 20 MU

Assume, all participants are endowed with 20 MU. If you contribute your 20 MU to the common pool, while the three other participants keep their 20 MU, how many MU would you and the others earn?

- You would earn 8 MU (20 MU * 1.6 / 4 = 8 MU) and the three other participants would earn 28 MU each (8 MU + 20 MU)
- You and the three other participants would earn 32 MU each (20 MU * 1.6 = 32 MU)
- The three other participants would earn 8 MU each (20 MU * 1.6 / 4 = 8 MU) and you would earn 28 MU (8 MU + 20 MU)
- You would earn 0 MU and the three other participants would earn 20 MU

**Figure S10. Comprehension questions (continued).**

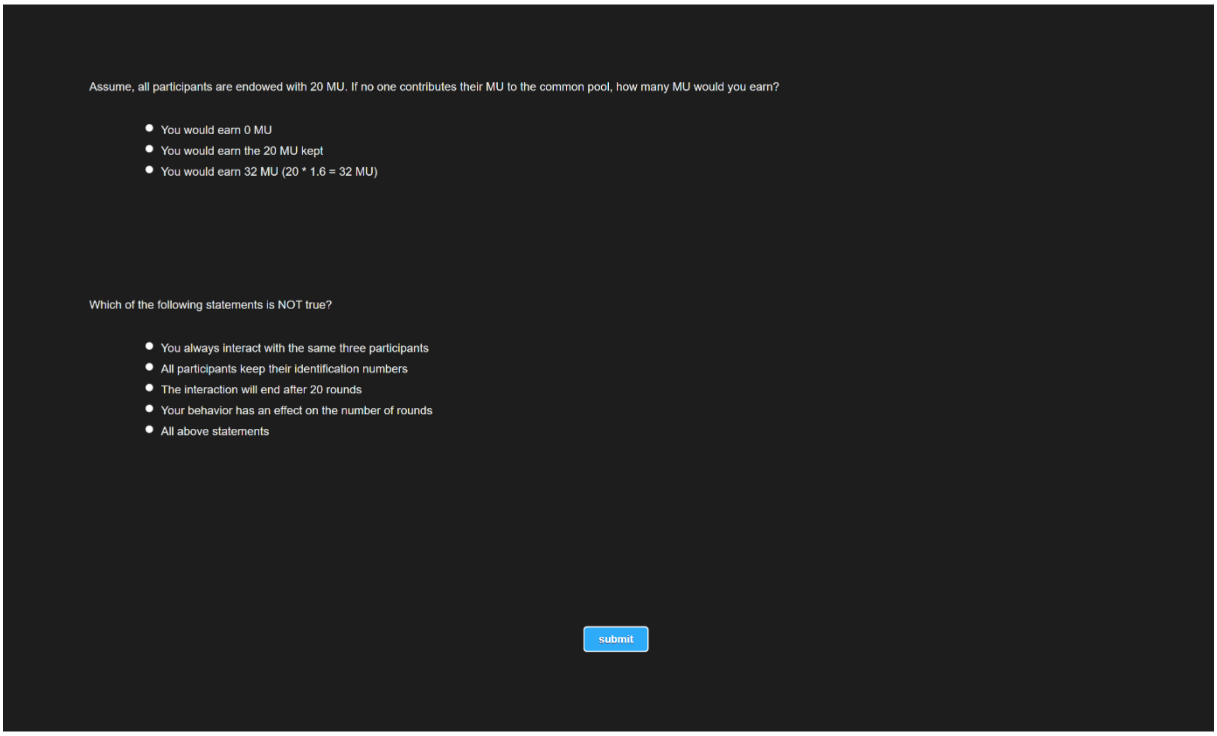

Assume, all participants are endowed with 20 MU. If no one contributes their MU to the common pool, how many MU would you earn?

- You would earn 0 MU
- You would earn the 20 MU kept
- You would earn 32 MU (20 * 1.6 = 32 MU)

Which of the following statements is NOT true?

- You always interact with the same three participants
- All participants keep their identification numbers
- The interaction will end after 20 rounds
- Your behavior has an effect on the number of rounds
- All above statements

submit

**Figure S10. Comprehension questions (continued).**

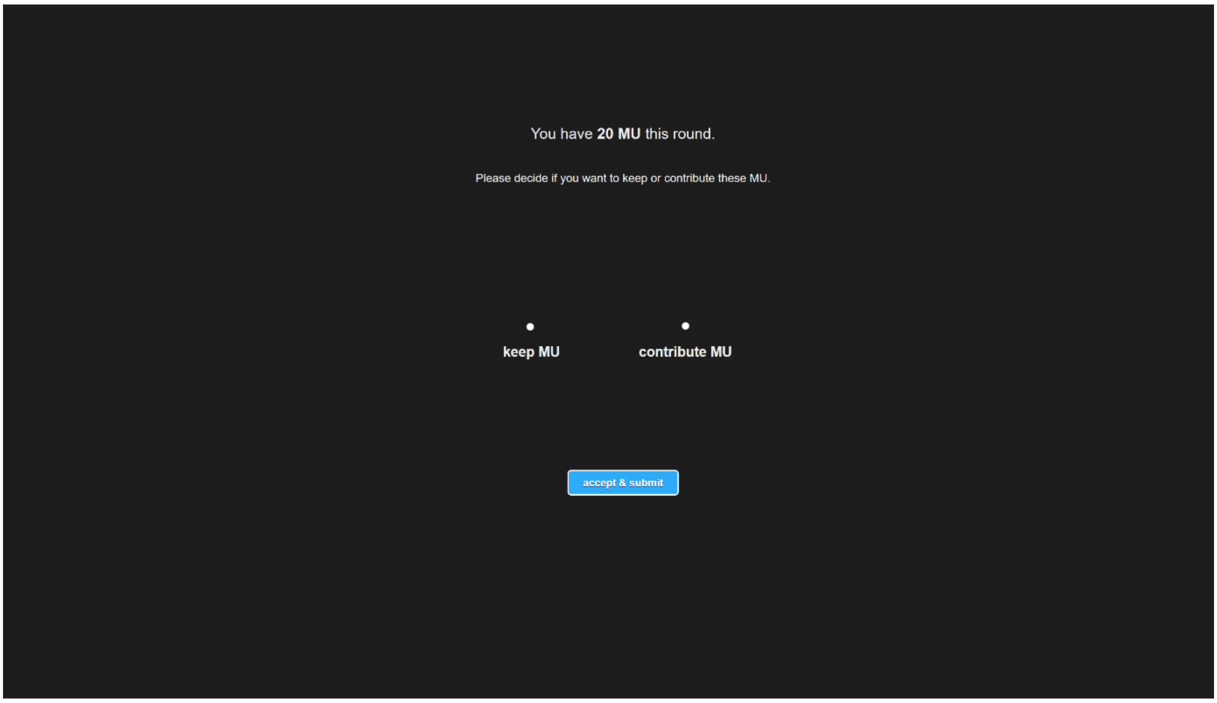


**Figure S11. Contribution stage.** Example of an endowed participant.

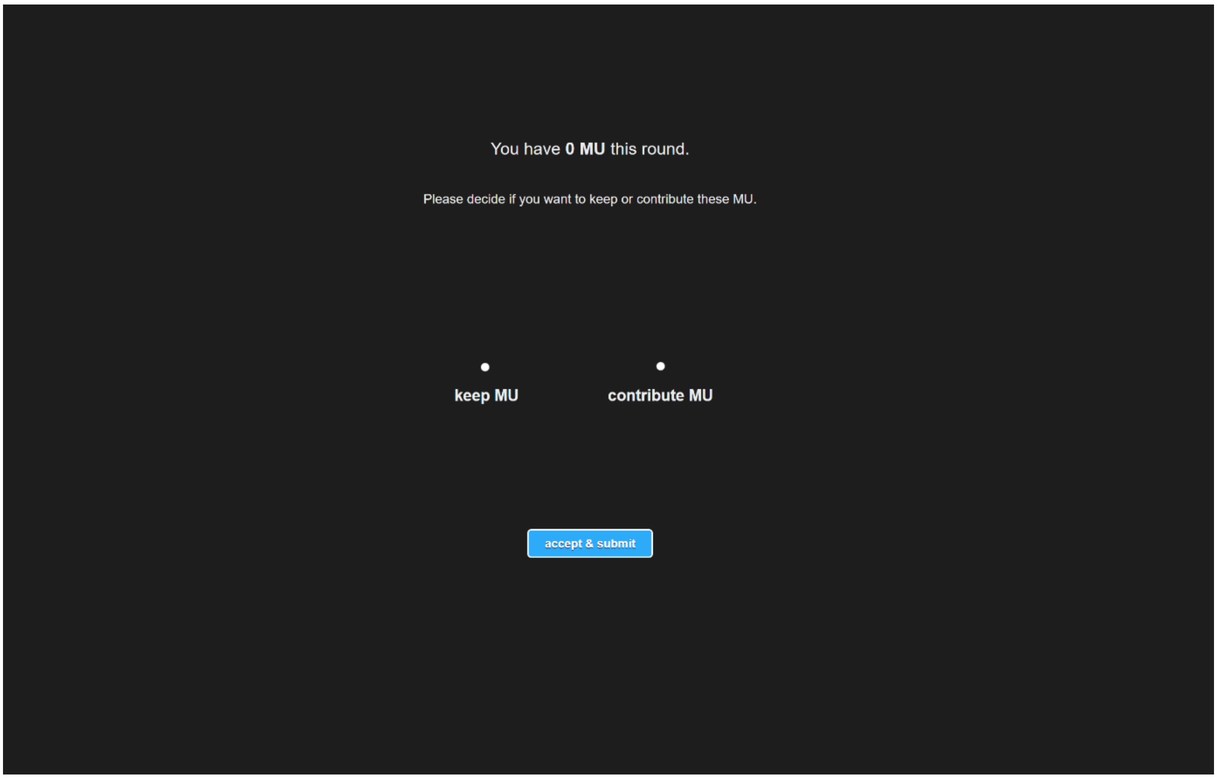


**Figure S12. Contribution stage.** Example of a not endowed participant.

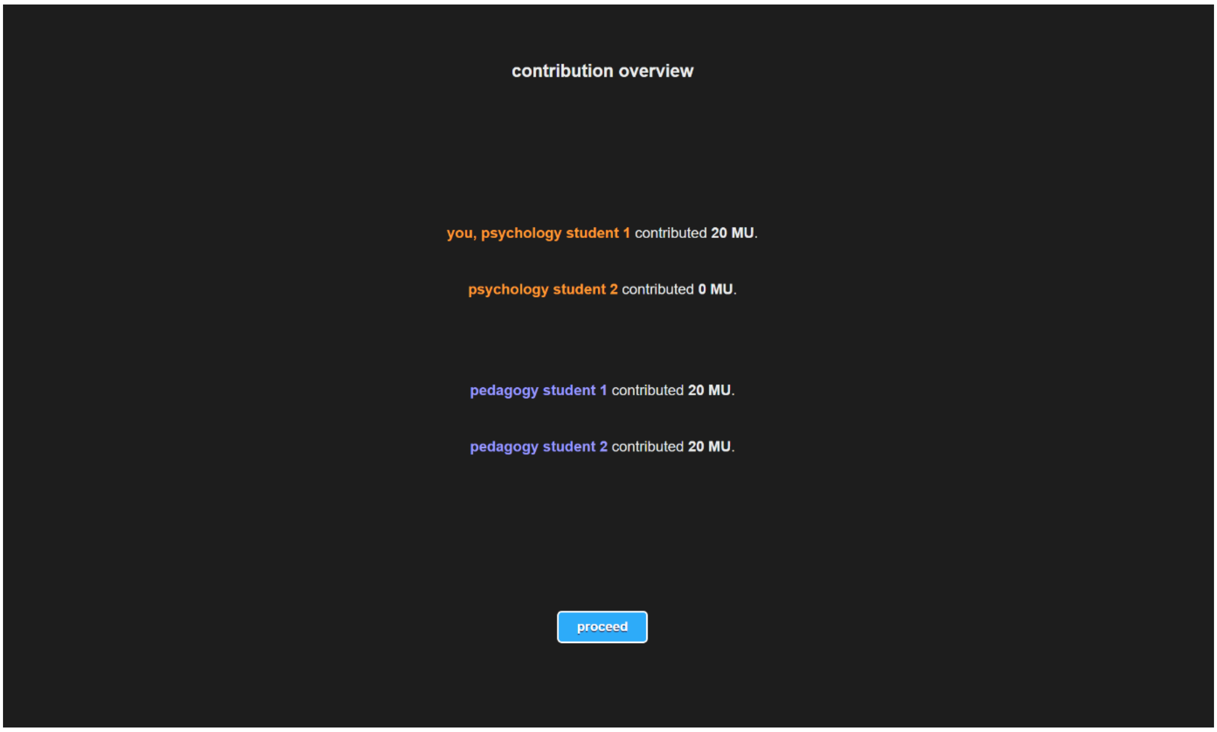


**Figure S13. Contribution feedback.** Example of psychology student.

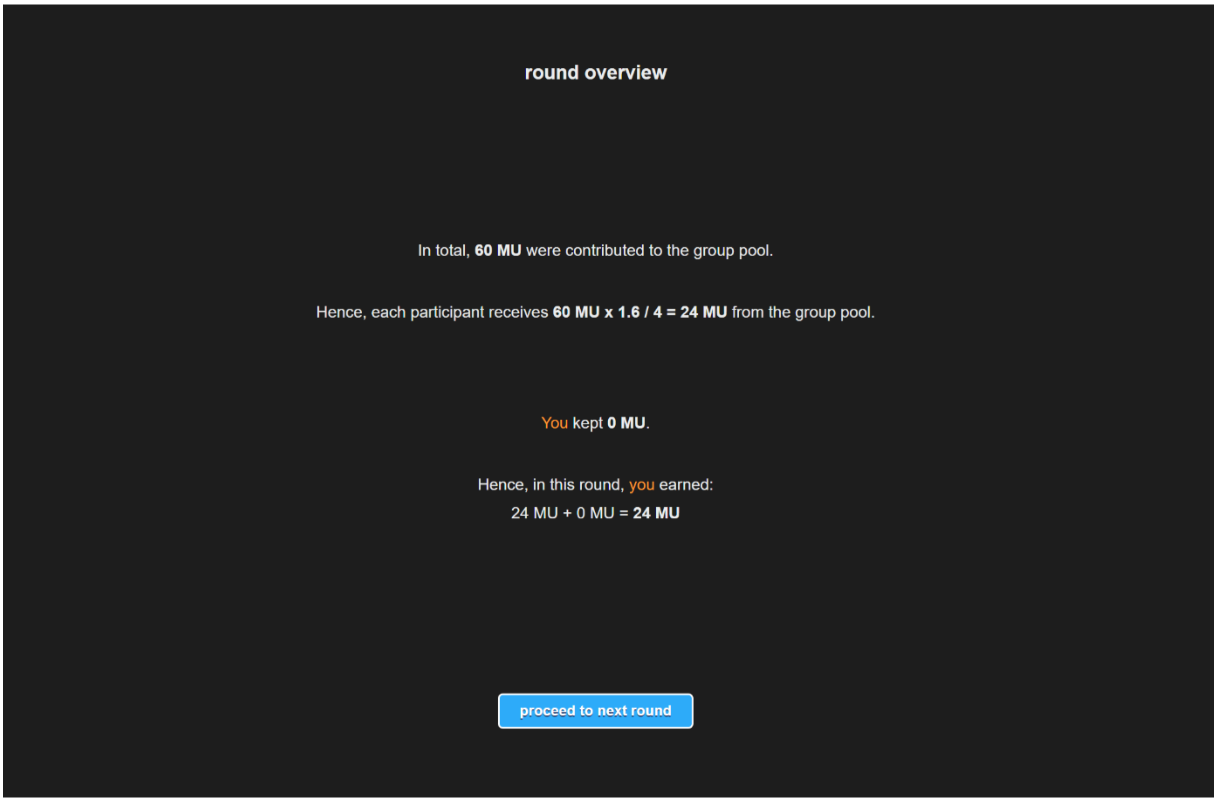


**Figure S14. Round feedback.** Without punishment.

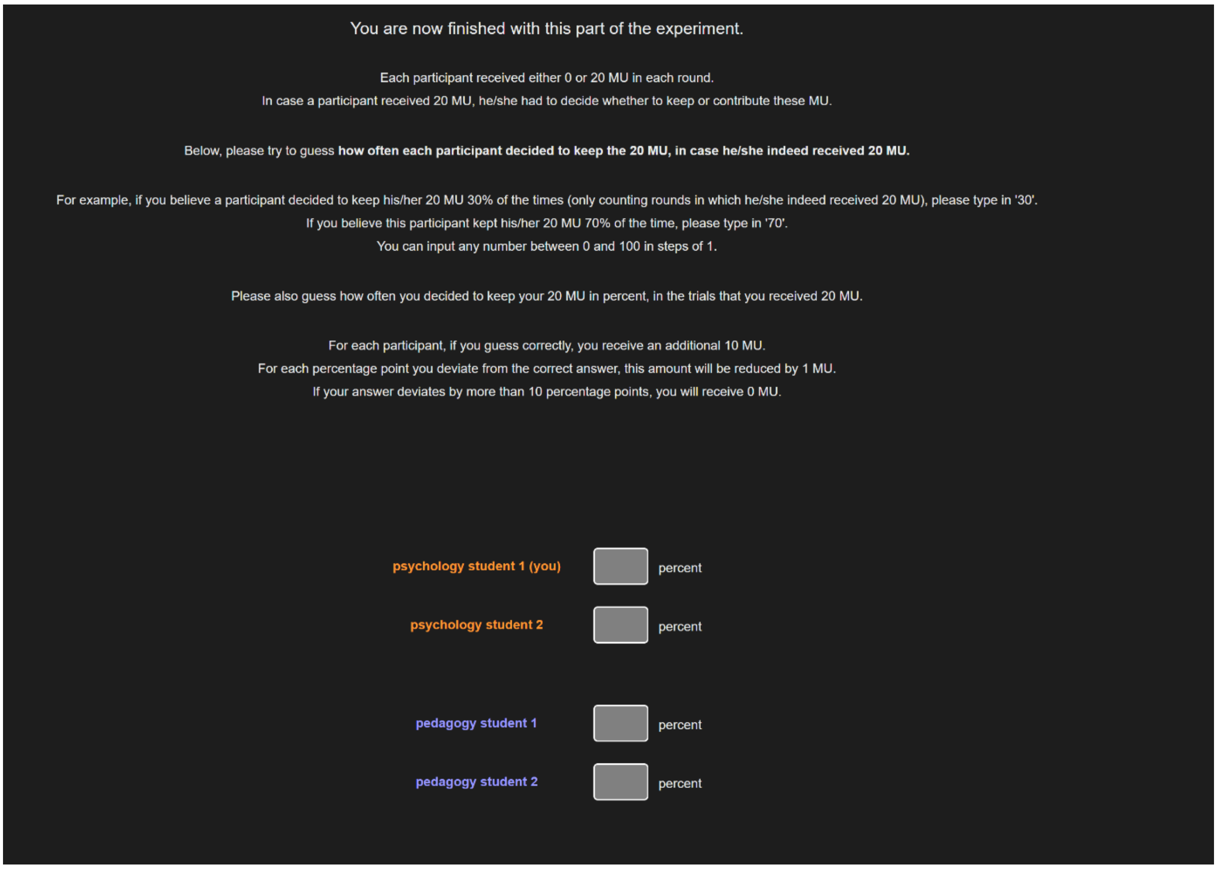


**Figure S15. Beliefs assessment.** Example of psychology student.

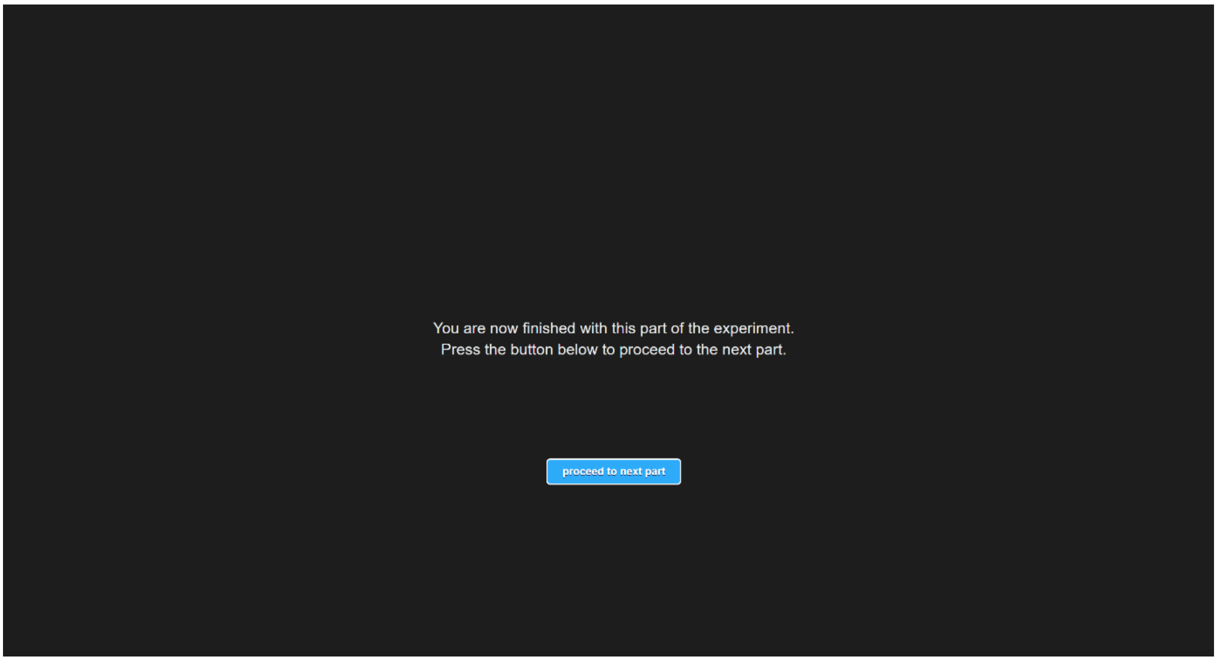


**Figure S16. Transition page.**

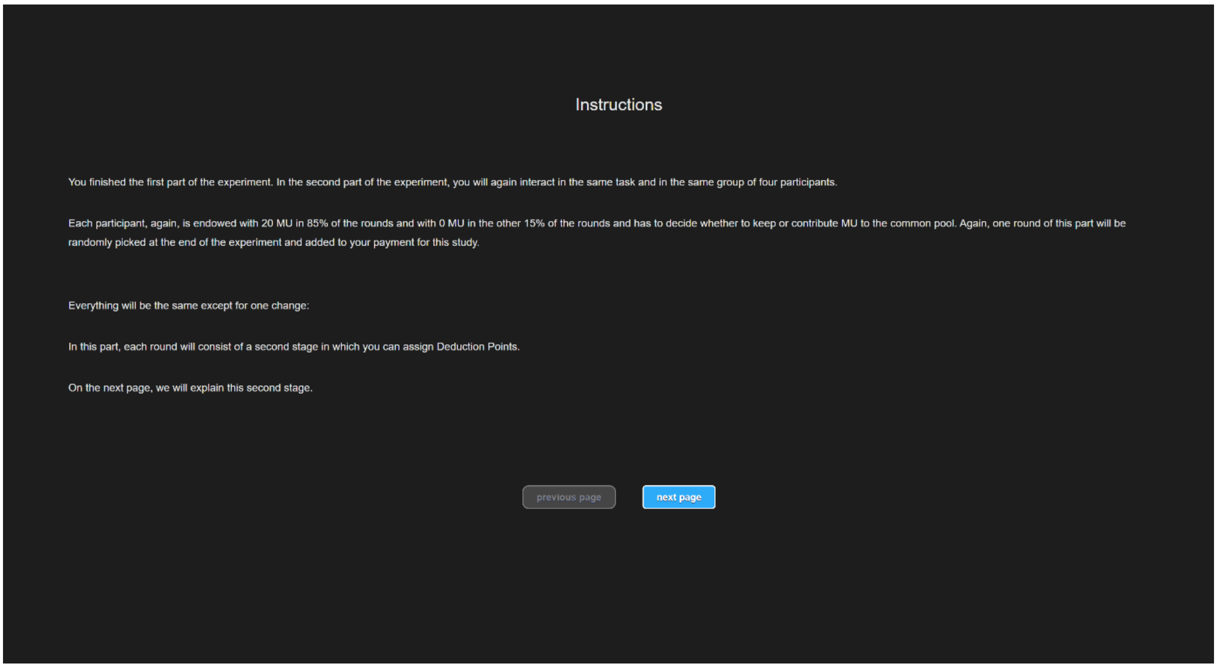


**Figure S17. Second block instruction page.** Example of second block with punishment.

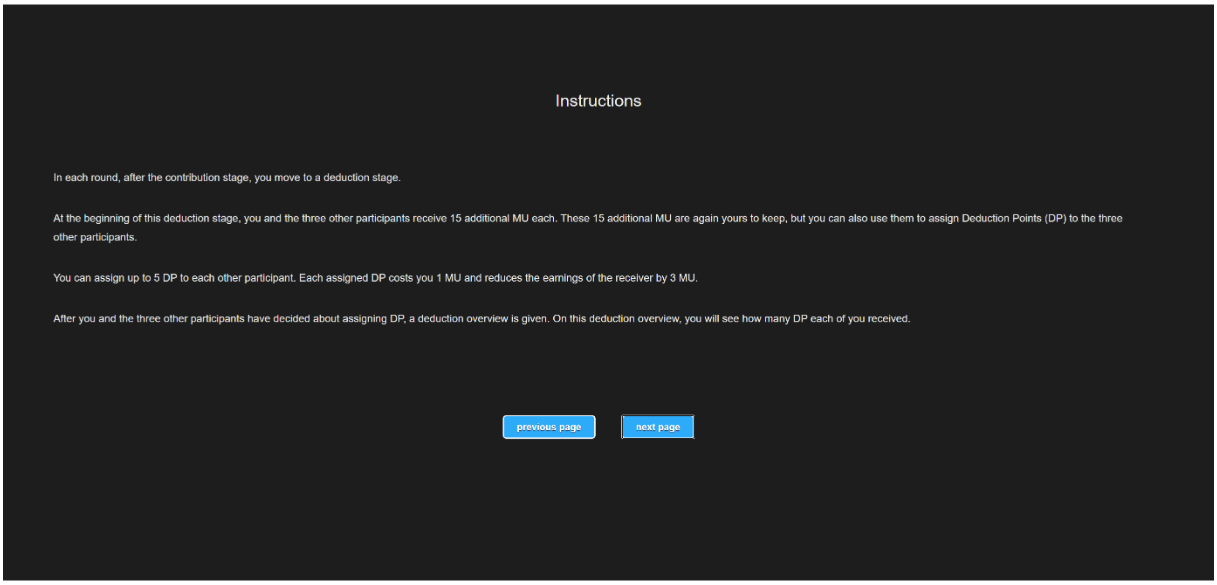


**Figure S18. Punishment instruction page.**

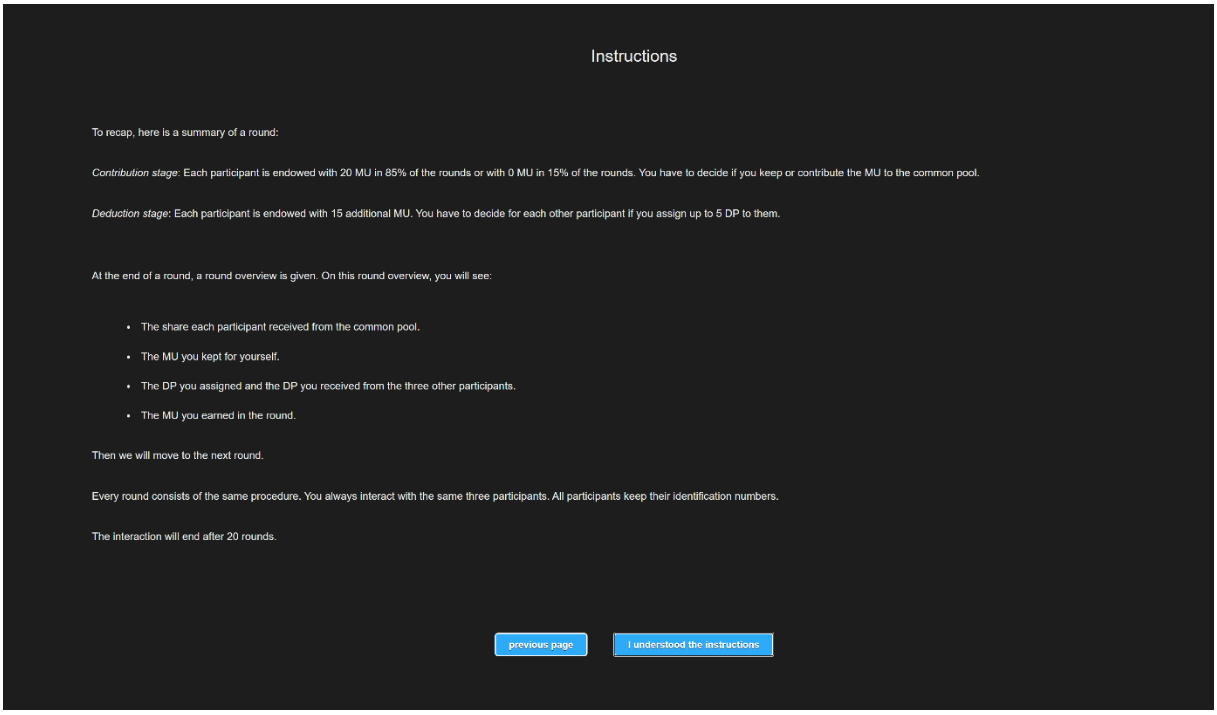


**Figure S19. Recap page.** With punishment.

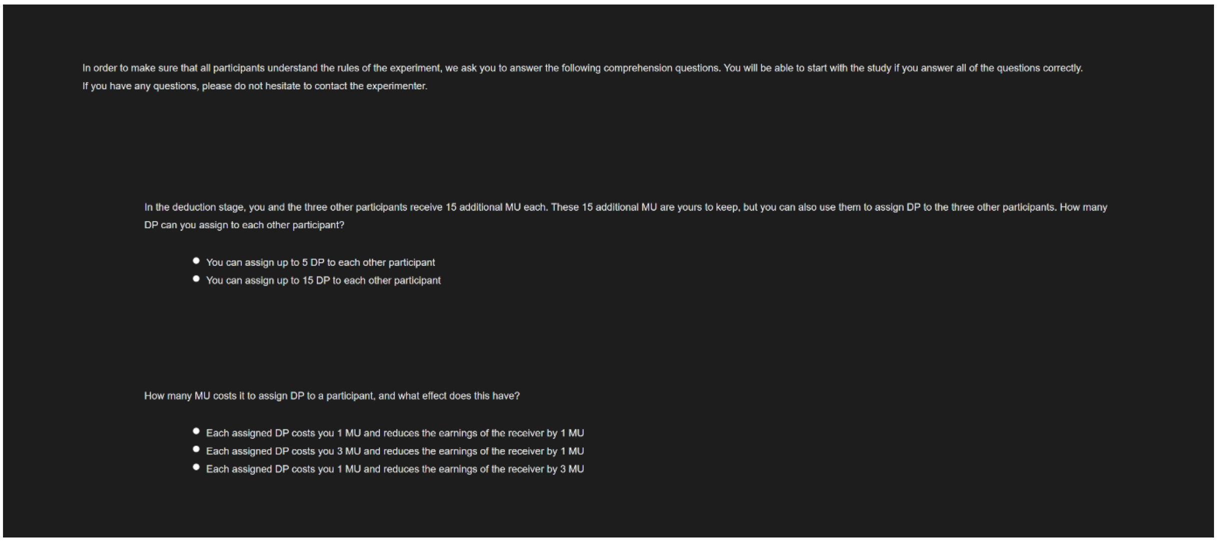


**Figure S20. Comprehension questions punishment.**

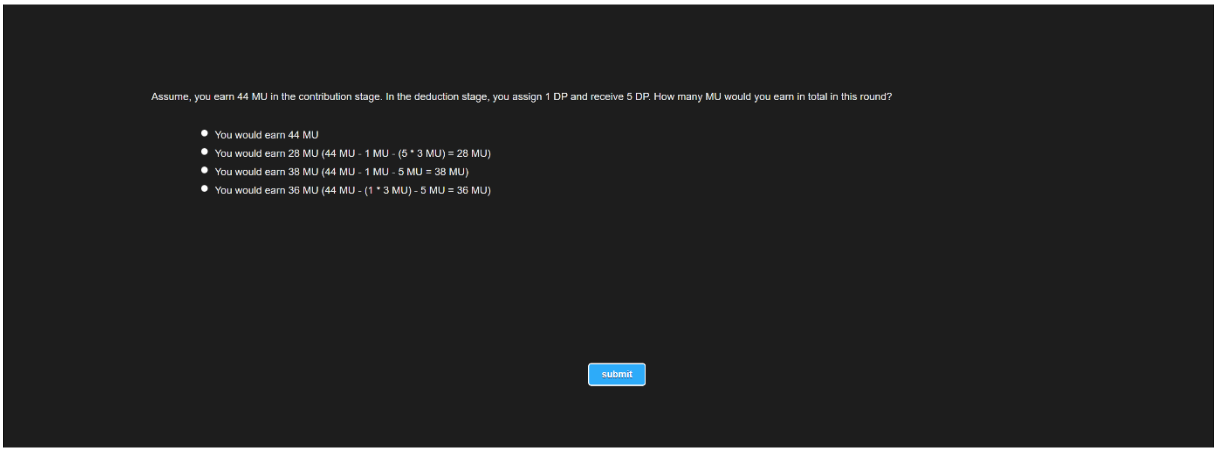


**Figure S20. Comprehension questions punishment (continued).**

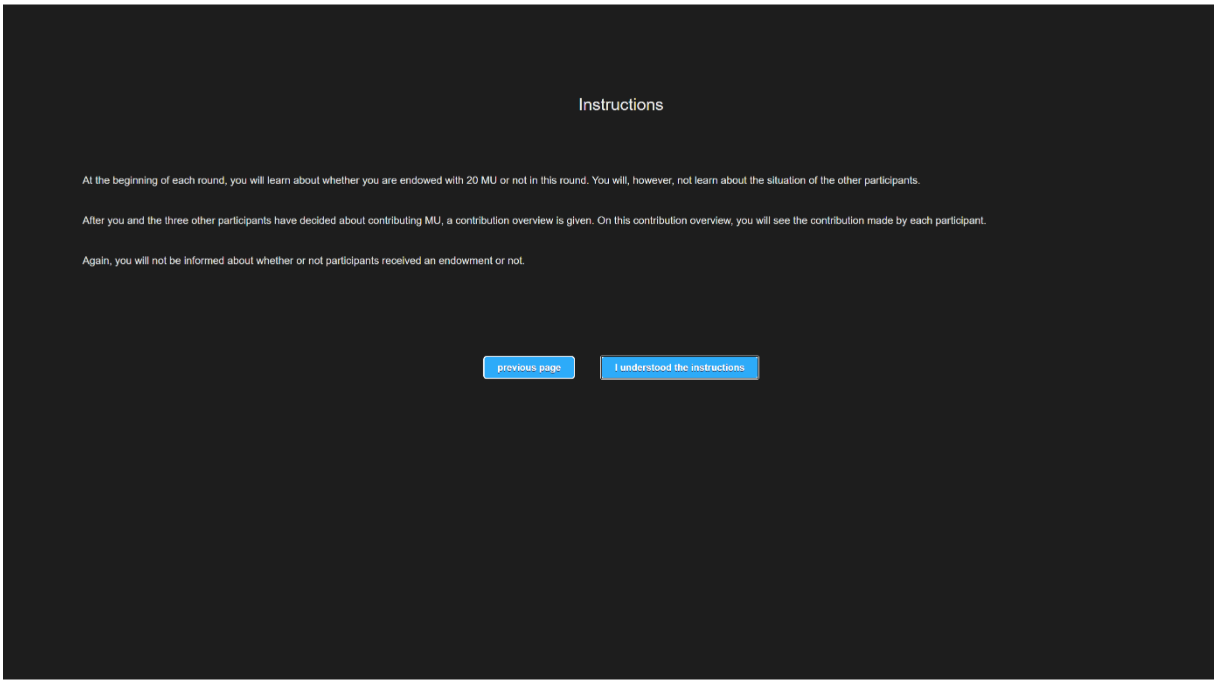


**Figure S21. Final second block instruction page.**

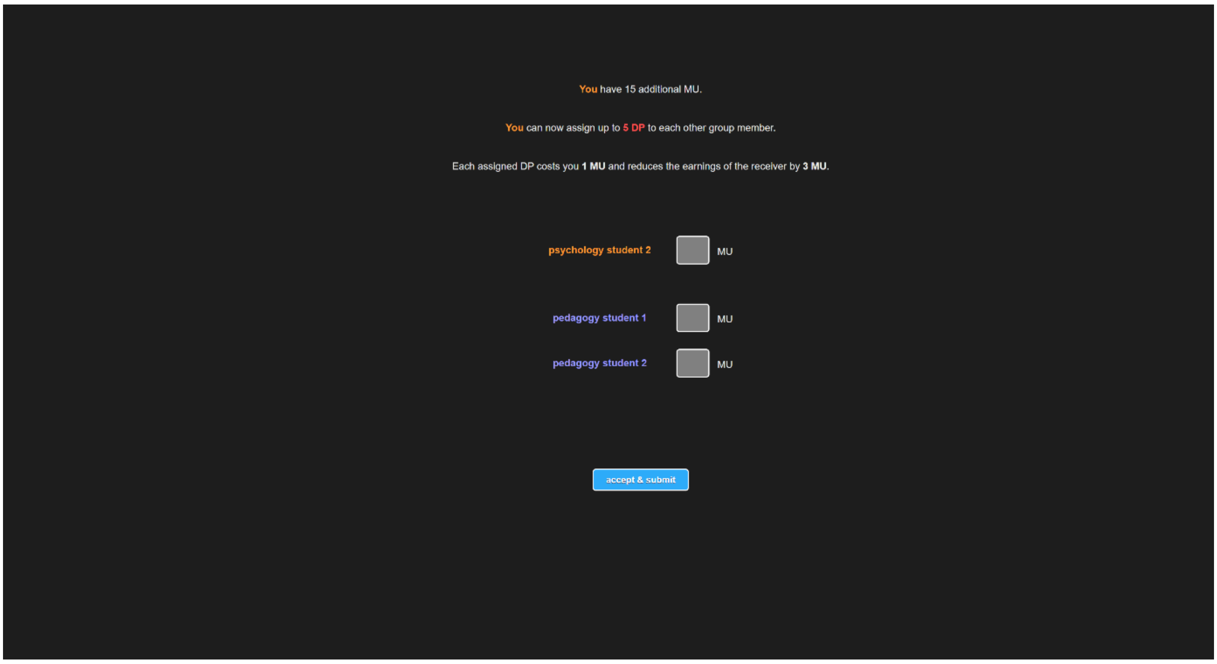


**Figure S22. Punishment stage.** Example of psychology student.

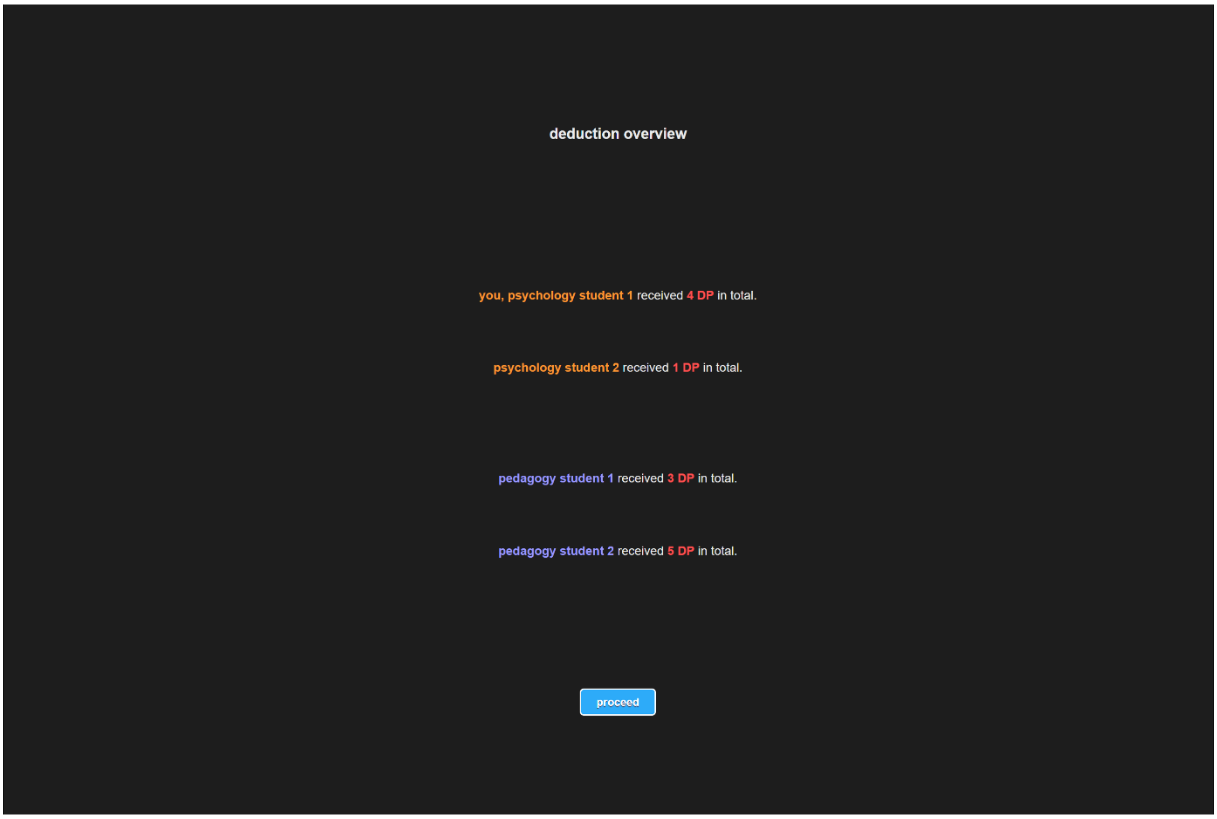


**Figure S23. Punishment feedback.** Example of psychology student.

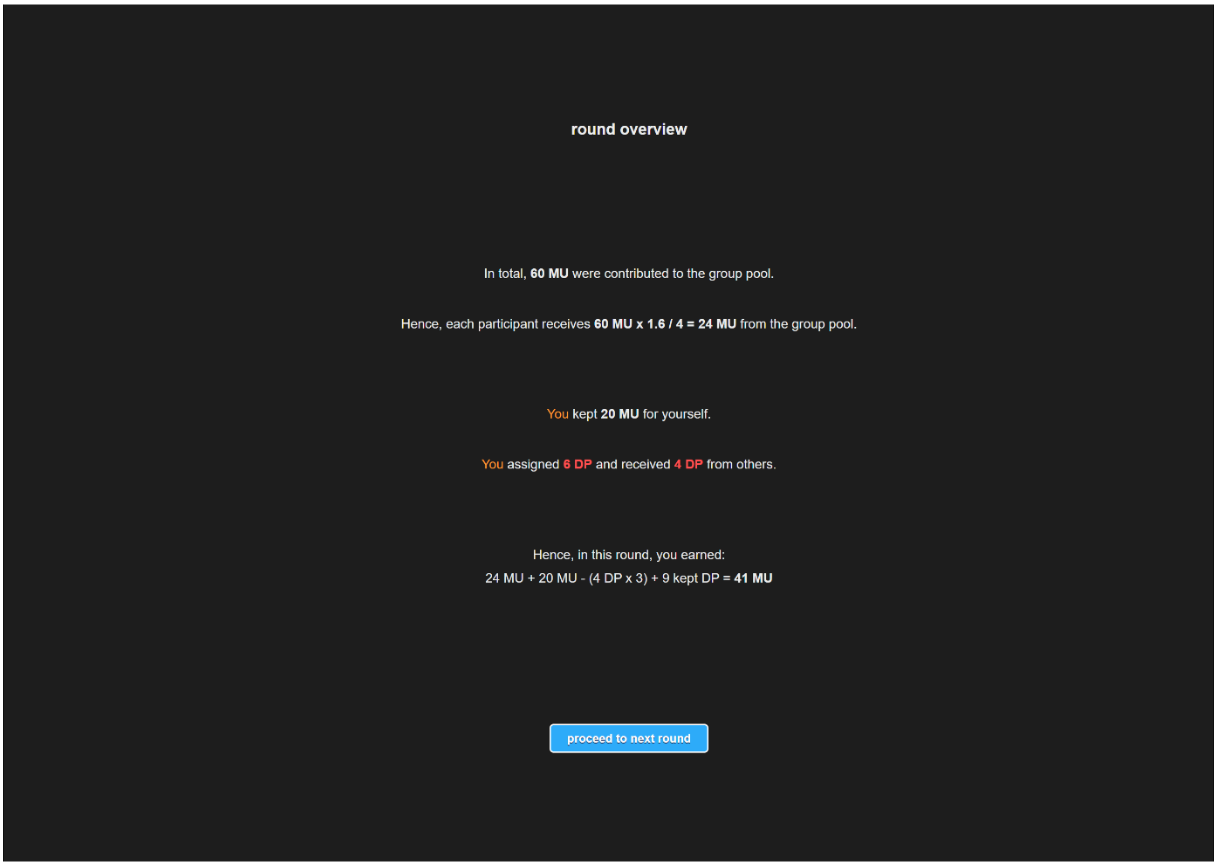


**Figure S24. Round feedback.** With punishment.

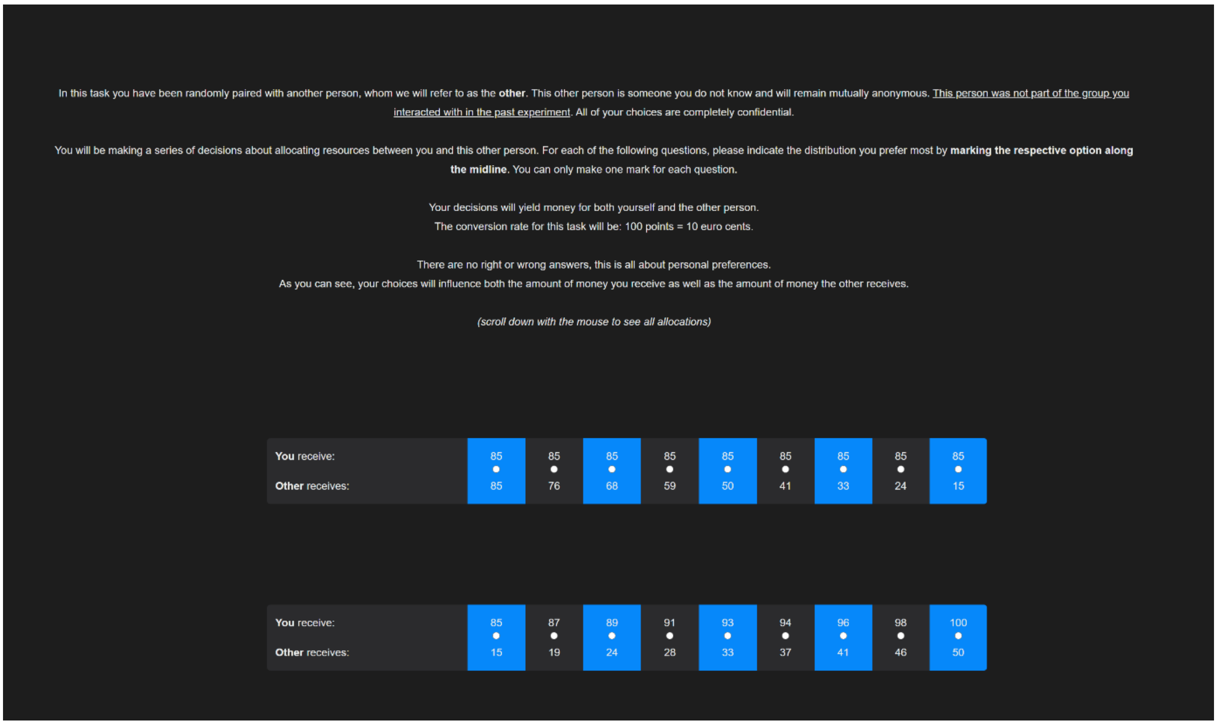


**Figure S25. Assessment of SVO.**

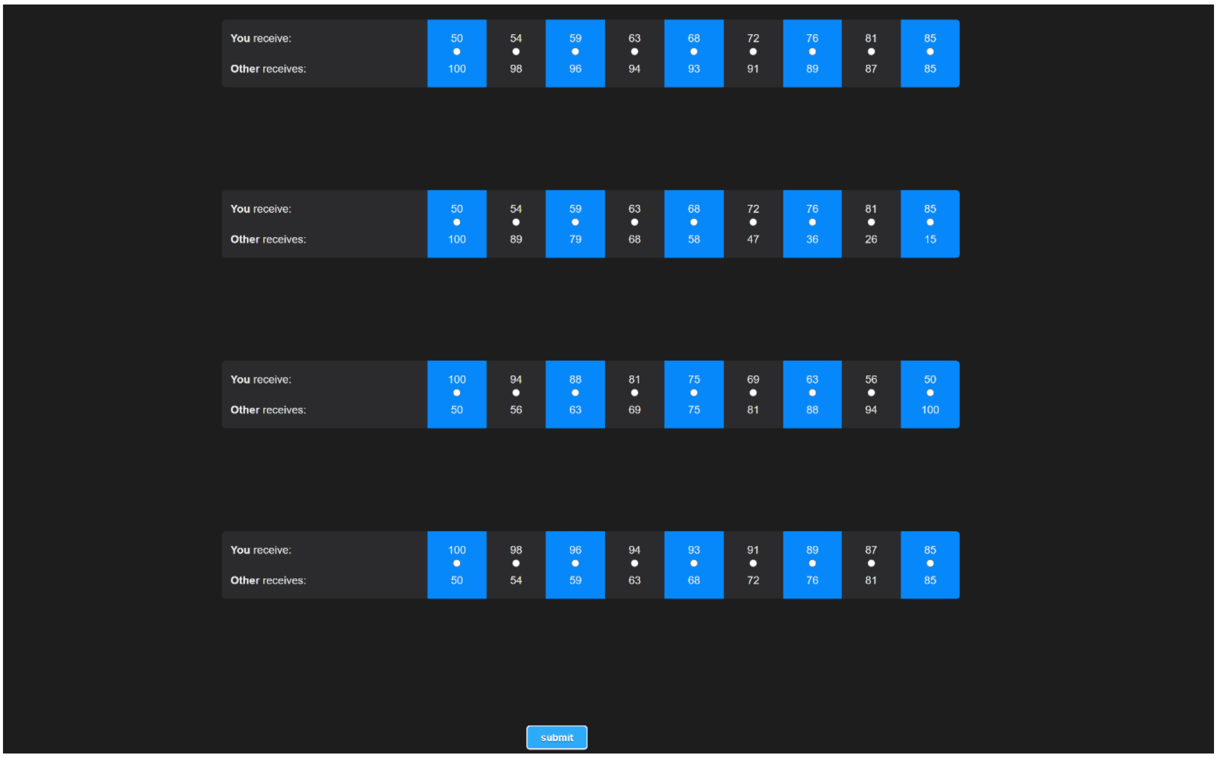


**Figure S25. Assessment of SVO (continued).**

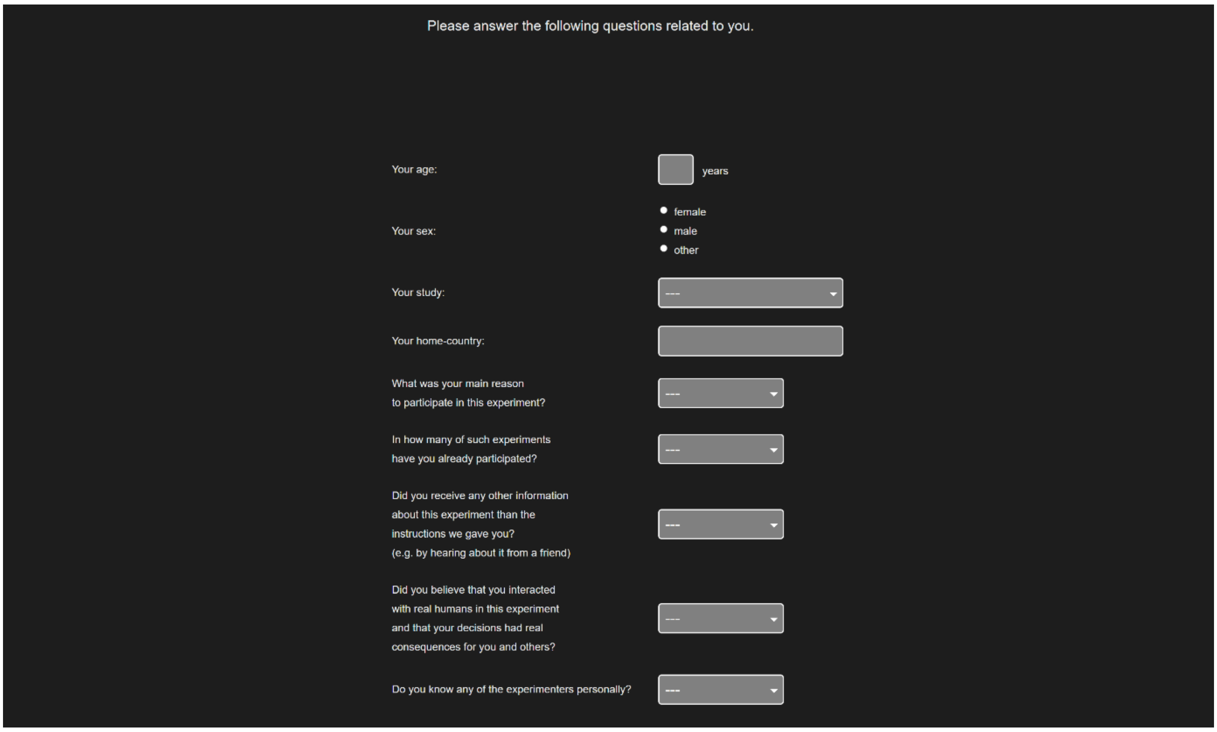


**Figure S26. Assessment of demographics and other questions.**

### 1.2.2. Experiment 2

*Participants and Experimental Design*

Experiment 2 was conducted in the behavioural laboratory, located in the building of the Faculty of Social and Behavioural Sciences of Leiden University. A total of 276 first-year psychology students ($n$ = 147) and pedagogy students ($n$ = 129) from this university participated (232 women and 44 men; $M_{age}$ = 19.14, $SD_{age}$ = 2.15 years). The sample size was determined based on feasibility concerns rather than a priori power calculations (see *Supplementary Results* for a sensitivity analysis). Given the number of first-year students in the study programmes Psychology and Pedagogical Science, and the time available in the laboratory, we aimed to create 52 pluriform groups (requiring 156 psychology students and 156 pedagogy students).

To examine punishment behaviour among both freshmen and relatively more established psychology and pedagogy students, the data was collected both at the start of the first semester and during the second semester of the academic year (we aimed to create 26 groups in each semester). Participants were allowed to take part in the experiment only once, either in the first semester ($n$ = 175) or the second semester ($n$ = 101), and they were randomly assigned to either the give-some treatment ($n$ = 138) or the take-some treatment ($n$ = 138), while keeping the distribution of psychology students and pedagogy students equal across treatments. We initially recruited 278 participants, but later had to exclude 2 participants because their decisions were not recorded correctly due to a technical error.

Throughout the instructions, it was noted several times that the interactions with the other psychology and pedagogy students were not live, but that they would specify binding decision schemas for the interactions (i.e., we used the so-called strategy method). An advantage of the strategy method is that we collected information about punishment in response to all potential decisions that participants could make, which increased the statistical power of our results and

allowed us to observe the complete conditional strategy of participants. After the data of all participants in the experiment were collected, it was randomly determined who interacted with whom, and each participant's outcome was calculated based on their actual decisions and punishment strategies. The total amount of Monetary Units (MU) they earned was converted to euros at the following rates: 10 MU = € 0.50. Participants could earn between €0 and €14.25. They earned, on average, €7.75. Two weeks after the experiment, participants could collect their additional payments in cash. In addition to the money, participants also received a personal feedback sheet that provided complete information about how their additional payment was calculated.

Experiment 2 consisted of the following stages: A public goods game stage (S1), and a third-party punishment game stage (S2). At S1, participants faced a linear one-shot PGG, which was either presented as give-some or take-some game, depending on the treatment participants were in. Participants performed the PGG in a pluriform group with two students from their own study programme and three students from the other study programme, i.e., a 6-person group with 3 psychology students and 3 pedagogy students. At S2, participants performed a third-party punishment game (TPG) in response to the contribution decisions (in the give-some treatment) or consumption decisions (in the take-some treatment) by members of another 6-person group. That is, as third parties with individual punishment capacity, they oversaw public good provision by another pluriform group.

*Experimental Procedure*

Upon arrival in the laboratory, participants were seated in individual cubicles, each containing a personal computer that was used to present the instructions and register their decisions. The experiment always began by informing participants that they would engage in a group decision-making task in which they would interact with fellow students from the study programmes

Psychology and Pedagogical Science, and an assessment of the extent to which they felt affiliated with other students from each of these study programmes (see *Materials* below).

The instructions explained to participants that the group decision making task consisted of a stage in which they had to decide to what extent they served their own interest or the interest of a group (S1), and a stage in which they could decrease the outcomes of persons in another group (S2). Specifically, participants learned that in S1 they were part of a 6-person group with students from both the study programmes Psychology and Pedagogical Science.

In the give-some treatment, participants learned that in S1 each person in the 6-person group was endowed with 100 MU and could give between 0 to 100 MU (in steps of 10 MU) to a group account. The MU given to the group account would be multiplied by 1.5 and divided equally among the entire 6-person group, and the MU kept for oneself would be transferred to the participant's private account. We refer to the MU given to the group account as contributions, and to the MU kept for oneself as non-contributions. In the take-some treatment, participants learned that in S1 each person in the 6-person group could take between 0 to 100 MU (in steps of 10 MU) from a group account of 600 MU. The MU taken from the group account would be transferred to the participant's private account, and the MU left in the group account would be multiplied by 1.5 and divided equally among the entire 6-person group. We refer to the MU taken from the group account as consumptions, and to the MU left in the group account as non-consumptions.

Note that across the two treatments, the two versions of the PGG had the same underlying outcome structure and were thus structurally equivalent[4]. In both treatments, the cost of cooperation was higher than the individual return, because each contribution (give-some treatment) or non-consumption (take-some treatment) of 10 MU resulted in a group return of 15 MU (10 x 1.5) and an individual return of 2.5 MU (15 / 6). Therefore, it was always in the

material self-interest of any participant to free-ride on the other group members' cooperation by non-contributing/consuming all MU.

It was further explained that participants could increase either the joint outcome of their 6-person group by contributing MU to the group account (in the give-some treatment) or non-consuming MU from the group account (in the take-some treatment), or their individual outcome by non-contributing MU to the group account (in the give-some treatment) or consuming MU from the group account (in the take-some treatment). Examples were given of possible scenarios in S1 (e.g., when one group member would free-ride, when none of the group members would cooperate). Following the detailed instructions about S1, the participants received comprehension questions to test their understanding of S1 (comparable to the comprehension questions of Experiment 1), with feedback on the correct answer after each question.

We then repeated that each 6-person group would consist of 3 psychology students and 3 pedagogy students, and emphasized the interdependence among the two subgroups within the larger group. Next, we assessed participants general trust toward psychology and pedagogy students, and how threatened they felt by psychology and pedagogy students (see *Materials* below).

Before participants made their contribution/consumption decision in S1, they were first instructed about S2. Participants learned that each group member was endowed with an additional 60 MU, which they could use to assign decrement points (DP) to members of another 6-person group (10 MU per person). For all possible contributions/consumptions in S1, participants could assign between 0 to 10 DP. Each DP reduced the final earnings of each punished target by three MU and would cost the punisher one MU. Thus, the self-to-other cost ratio of assigning a DP to someone was 1:3. The MU not used to assign DP would be transferred

to the participant's private account. Participants learned that they had to specify their response strategy twice: Once for contributions/consumptions made by psychology students and once for contributions/consumptions made by pedagogy students. Examples were given of possible scenarios in S2 (e.g., when multiple members of the other group would opt for a contribution/consumption for which the participant assigned DP).

While participants were third parties with individual punishment capacity, overseeing the contribution/consumption decisions of members in another pluriform group, yet another pluriform group would oversee the contribution/consumption decisions of their own pluriform group. That is, participants learned that, just as they (group A) could assign DP to members of another 6-person group (group B), members of yet another 6-person group (group C) could assign DP to them and their fellow group members. Thus, participants learned that psychology and pedagogy students in another group could decrease their outcome from S1.

Finally, we reminded the participants that the 6-person groups would be randomly formed after all participants had taken part in the experiment, and that each participant's outcome was calculated based on their actual decisions in S1 and S2. Importantly, there was a closed envelope present in each cubicle, which contained an example of the feedback sheet that participants would receive when collecting their additional payment in cash (*Figure S27*), and at this stage of the instructions, participants were asked to examine the feedback sheet to get an idea of what information would be provided. Following the detailed instructions about S2, the participants received comprehension questions to test their understanding of the entire experimental procedures (including S1 and S2), with feedback on the correct answer after each question.

After the instructions of S1 and S2, participants first made their contribution/consumption decision (S1) and then specified their response strategies towards the other group (S2). In S1,

participants indicated how many MU they contributed to the group account (give-some treatment) or consumed from the group account (take-some treatment) by selecting one of the eleven possible choices (0 to 100 MU, in steps of 10 MU). In S2, the eleven possible choices in S1 were listed and participants indicated for each how many DP they would like to assign if the others would opt for that particular contribution/consumption by typing in a number of DP (0 to 10). After typing in a number, the costs in MU of assigning that number of DP for the participant and the receiver were both shown. Participants specified their assignment of DP once for the 3 psychology students and once for the 3 pedagogy students. To control for sequence effects, whether they first specified their response strategy for psychology or pedagogy students was counterbalanced between participants.

Next, it was explained that there would be a chance that 6-person groups consisting of 3 psychology students and 3 pedagogy students could not be created, and participants were asked whether and how they would want to change their response strategies if the composition would be either 4 psychology students and 2 pedagogy students (i.e., majority of psychology students) or the other way around (i.e., majority of pedagogy students). Participants were shown the response strategies they had specified before and could change them for each of the two alternative compositions (order counterbalanced between participants). Finally, we assessed participants' general positive and negative perceptions of psychology and pedagogy students (see *Materials* below). We also included an assessment of social value orientation, but due to a technical error we had to drop this measure. At the end of the experiment, participants were thoroughly debriefed, were given instructions about how to collect their additional payments, and were thanked for their participation.

*Materials*

To assess the extent to which participants felt affiliated with other students from the study programmes Psychology and Pedagogical Science, they rated the applicability of four statements on a 7-point Likert scale ranging from 1 (*disagree*) to 7 (*agree*), twice: Once about psychology students and once about pedagogy students ("I identify with psychology/pedagogy students," "I feel connected to psychology/pedagogy students," "I feel involved with psychology/pedagogy students," and "I see myself as belonging to the group of psychology/pedagogy students;" adapted from[1,2]; $\alpha_{own} = 0.85$, $\alpha_{other} = 0.84$).

To assess the extent to which participants generally trust other students from the study programmes Psychology and Pedagogical Science, they rated the applicability of eight statements on a 7-point Likert scale ranging from 1 (*disagree*) to 7 (*agree*), twice: Once about psychology students and once about pedagogy students ("I believe that psychology/pedagogy students tend to keep/take many MU for themselves," "I believe that psychology/pedagogy students tend to think about their self-interest," "I believe that psychology/pedagogy students tend to put self-interest above group interest," "I believe that psychology/pedagogy students tend to give/leave few MU to/in the group account," "I believe that psychology/pedagogy students can be trusted to put their self-interest aside," "I believe that psychology/pedagogy students can be trusted to think about the interest of the group," "I believe that psychology/pedagogy students can be trusted to do something good for the group," "I believe that psychology/pedagogy students can be trusted to contribute many MU to the group account/consume few MU from the group account;" adapted from[5,6]; $\alpha_{own} = 0.92$, $\alpha_{other} = 0.91$).

To assess the extent to which participants felt threatened by other students from the study programmes Psychology and Pedagogical Science, they rated the applicability of two statements on a 7-point Likert scale ranging from 1 (*disagree*) to 7 (*agree*), twice: Once about

psychology students and once about pedagogy students (“When I think about psychology/pedagogy students giving few MU to the group account/taking many MU from the group account, I feel threatened,” “When I think about psychology/pedagogy students giving few MU to the group account/taking many MU from the group account, I feel attacked;” adapted from[1]; $\alpha_{own} = 0.86$, $\alpha_{other} = 0.85$).

To assess participants’ general positive perceptions of other students from the study programmes Psychology and Pedagogical Science, they rated the applicability of four statements on a 7-point Likert scale ranging from 1 (*disagree*) to 7 (*agree*), twice: Once about psychology students and once about pedagogy students (“I generally find psychology/pedagogy students generous,” “I generally find psychology/pedagogy students helpful,” “I generally find psychology/pedagogy students bounteous,” “I generally find psychology/pedagogy students social;” $\alpha_{own} = 0.78$, $\alpha_{other} = 0.79$).

To assess participants’ general negative perceptions of other students from the study programmes Psychology and Pedagogical Science, they rated the applicability of four statements on a 7-point Likert scale ranging from 1 (*disagree*) to 7 (*agree*), twice: Once about psychology students and once about pedagogy students (“I generally find psychology/pedagogy students greedy,” “I generally find psychology/pedagogy students covetous,” “I generally find psychology/pedagogy students stingy,” “I generally find psychology/pedagogy students selfish;” $\alpha_{own} = 0.90$, $\alpha_{other} = 0.90$).

ID code: ____________

Punten: ________ [1 punt = 0,05] Euro's: ____________

FASE 1

| Groep ID: | ID codes: | Gepakt/gegeven: | Opbrengst fase 1: |
|---|---|---|---|
| PSY-student 1 | | | |
| PSY-student 2 | | | |
| PSY-student 3 | | | |
| PEDA-student 1 | | | |
| PEDA-student 2 | | | |
| PEDA-student 3 | | | |

Inhoud gezamenlijke pot: ________ [x 1,5=] ________

ONTVANGEN VERLAGINGSPUNTEN

| Groep ID: | ID codes: | Aantal verlagingspunten: |
|---|---|---|
| PSY-student 1 | | |
| PSY-student 2 | | |
| PSY-student 3 | | |
| PEDA-student 1 | | |
| PEDA-student 2 | | |
| PEDA-student 3 | | |

Totaal aantal punten ontvangen: ________ [x 3 =] ________

TOEGEWEZEN VERLAGINGSPUNTEN

| Groep ID: | ID codes: | Aantal verlagingspunten: |
|---|---|---|
| PSY-student 1 | | |
| PSY-student 2 | | |
| PSY-student 3 | | |
| PEDA-student 1 | | |
| PEDA-student 2 | | |
| PEDA-student 3 | | |

Totaal aantal punten toegewezen: 60 punten - ________

________ - ________ + ________ = ________

**Figure S27. Example of the feedback sheet.**

### 1.2.3. Experiment 3

*Participants and Experimental Design*

Experiment 3 was conducted in the behavioural laboratory, located in the building of the Faculty of Social and Behavioural Sciences of Leiden University. A total of 179 first-year psychology students ($n$ = 90) and pedagogy students ($n$ = 89) from this university participated (150 women and 29 men; $M_{age}$ = 19.06, $SD_{age}$ = 2.30 years). The sample size was determined based on feasibility concerns rather than a priori power calculations (see *Supplementary Results* for a sensitivity analysis). Given the number of first-year students in the study programmes Psychology and Pedagogical Science, and the time available in the laboratory, we aimed to create 32 pluriform groups (requiring 96 psychology students and 96 pedagogy students). The data were collected in the first semester of the academic year. Participants were randomly assigned to either the give-some treatment ($n$ = 89) or the take-some treatment ($n$ = 90), while keeping the distribution of psychology students and pedagogy students equal across treatments.

The research approach was similar to Experiment 2. We again used the strategy method and randomly determined who interacted with whom after the data of all participants in the experiment was collected. The total amount of MU participants earned was converted to euros at the following rates: 10 MU = € 0.25. Participants could earn between €0 euros and €14.25. They earned, on average, €7.81. Two weeks after the experiment, participants could collect their additional payments in cash. In addition to the money, and similar to Experiment 2, participants also received a personal feedback sheet that provided complete information about how their additional payment was calculated.

Experiment 3 consisted of the following stages: A public goods game stage (S1), and a third-party punishment game stage (S2). At S1, participants faced two linear one-shot PGG, which were either presented as give-some or take-some game, depending on the treatment participants

were in. First, participants performed a PGG in a uniform group with two students from their own study programme, i.e., a 3-person group with either psychology or pedagogy students. Second, participants performed a PGG in a pluriform group with two students from their own study programme and three students from the other study programme, i.e., a 6-person group with 3 psychology students and 3 pedagogy students. At S2, participants performed a TPG in response to the contribution decisions (in the give-some treatment) or consumption decisions (in the take-some treatment) by members of two other 3-person groups (one with psychology students and one with pedagogy students) and members of one other 6-person group. That is, as third parties with individual punishment capacity, they oversaw public good provision by two other uniform groups and one other pluriform group.

*Experimental Procedure*

The experimental procedure was similar to the procedure of Experiment 2, except for the number of PGG and TPG they faced. The instructions explained to participants that the group decision making task consisted of a stage in which they had to decide to what extent they served their own interest or the interest of the two groups (S1), and a stage in which they could decrease the outcomes of persons in other groups (S2). Specifically, participants learned that in S1 they would be a member of two different groups: A 3-person group with students from their own study programme (either psychology or pedagogy students), and a 6-person group with other students from both the study programmes Psychology and Pedagogical Science.

In the give-some treatment, participants learned that each person would be endowed with 100 MU and could contribute between 0 to 100 MU (in steps of 10 MU) to the group account of their 3-person group. The MU contributed to this group account would be multiplied by 1.5 and divided equally among the entire 3-person group, and the MU not contributed to this group would be transferred to the participant's private account. In addition, participants learned that

each person would be endowed with another 100 MU and could contribute between 0 to 100 MU (in steps of 10 MU) to a group account of their 6-person group. The MU contributed to this group account good would be multiplied by 1.5 and divided equally among the entire 6-person group, and the MU not contributed to this group account would be transferred to the person's private account.

In the take-some treatment, participants learned that each person could consume between 0 to 100 MU (in steps of 10 MU) from the group account of 300 MU of their 3-person group. The MU consumed from this group account would be transferred to the participant's private account, and the MU not consumed from this group account would be multiplied by 1.5 and divided equally among the entire 3-person group. In addition, participants learned that each person could also consume between 0 to 100 MU (in steps of 10 MU) from a group account of 600 MU of their 6-person group. The MU consumed from this group account would be transferred to the participant's private account, and the MU not consumed from this group account would be multiplied by 1.5 and divided equally among the entire 6-person group.

Note that the two contribution/consumption decisions were presented as independent decisions, involving different group accounts and different group members. Note also that across the two treatments, the two versions of the two PGG had the same underlying outcome structures and were thus structurally equivalent [4]. However, because the group size differed across the two PGG that participants faced (i.e., a 3-person versus a 6-person group), their underlying outcome structures were comparable but not exactly the same.

Similar to Experiment 2, participants were first instructed about S2 before they made their contribution/consumption decisions in S1. Participants learned that each group member was endowed with an additional 120 MU and could use these MU to assign decrement points (DP) to members of three other groups (10 MU per person). More specifically, it was explained that

they had to do this for members of (i) a 3-person group with psychology students, (ii) a 3-person group with pedagogy students, and (iii) a 6-person group with 3 psychology students and 3 pedagogy students. For all possible contributions/consumptions in S1, participants could assign between 0 and 10 DP to each member of the other group if they would opt for that particular contribution/consumption. Each DP reduced the final earnings of each punished target by three MU and cost the punisher one MU. Thus, the self-to-other cost ratio of assigning a DP to someone was 1:3. The MU not used to assign DP would be transferred to the participant's private account. Participants learned that they had to specify their four response strategies: Once for contributions/consumptions by psychology students in the 3-person group, once for contributions/consumptions by pedagogy students in the 3-person group, once for contributions/consumptions by psychology students in the 6-person group, and once for contributions/consumptions by pedagogy students in the 6-person group.

While participants were third parties with individual punishment capacity, overseeing the contribution/consumption decisions of members in two other uniform groups and one other pluriform group, other uniform and pluriform groups would oversee the contribution/consumption decisions of their own uniform and pluriform groups. That is, participants learned that, just as they (group A) could assign DP to members of two other 3-person groups (groups B), members of two other 3-person groups (groups C) could assign DP to them and their fellow group members. Thus, participants learned that psychology and pedagogy students in other 3 and 6-person groups could decrease their outcome from S1.

Similar to Experiment 2, participants were asked to examine the feedback sheet that they would receive when collecting their additional payment in case (*Figure S28*). Also similar to Experiment 2, participants first made their contribution/consumption decisions (S1) and then specified their response strategies (S2). In S1, participants always indicated first how many MU they contributed to the group account (give-some treatment) or consumed from the group

account (take-some treatment) by selecting one of the eleven possible choices (0 to 100 MU, in steps of 10 MU). Participants always indicated their contribution/consumption first for the 3-person group and then for the 6-person group. In S2, the eleven possible choices in S1 were listed and participants indicated for each how many DP they assigned if the others opted for that particular contribution/consumption by typing in a number of DP (0 to 10). After typing in a number, the costs in MU of assigning that number of DP for the participant and the receiver were both shown. Although participants always indicated their assignment of DP first for the 3-person groups and then for the 6-person group, whether they first specified their response strategy for psychology or pedagogy students was counterbalanced between participants.

*Materials*

We used the same measures as in Experiment 2 (see *Supplementary Methods*) to assess the extent to which participants (a) felt affiliated with other psychology and pedagogy students ($\alpha_{own}$ = 0.84, $\alpha_{other}$ = 0.85), (b) generally trusted other psychology and pedagogy students ($\alpha_{own}$ = 0.88, $\alpha_{other}$ = 0.91), and (c) felt threatened by other psychology and pedagogy students ($\alpha_{own}$ = 0.90, $\alpha_{other}$ = 0.89). Finally, we assessed (d) participants' positive perceptions ($\alpha_{own}$ = 0.75, $\alpha_{other}$ = 0.82) and (e) negative perceptions ($\alpha_{own}$ = 0.90, $\alpha_{other}$ = 0.89) of other psychology and pedagogy students.

ID code: ..............................

Punten: .................... [1 punt = 0,025] Euro's: ....................

FASE 1: **3-persoonsgroep**

| Groep ID: | ID codes: | Gepakt/gegeven: | Opbrengst fase 1: |
|---|---|---|---|
| PED/PSY student 1 | | | |
| PED/PSY student 2 | | | |
| PED/PSY-student 3 | | | |

Inhoud gezamenlijke pot: .................... [x 1,5=] ....................

ONTVANGEN VERLAGINGSPUNTEN

| Groep ID: | ID codes: | Aantal verlagingspunten: |
|---|---|---|
| PSY-student 1 | | |
| PSY-student 2 | | |
| PSY-student 3 | | |

| Groep ID: | ID codes: | Aantal verlagingspunten: |
|---|---|---|
| PEDA-student 1 | | |
| PEDA-student 2 | | |
| PEDA-student 3 | | |

Totaal aantal punten ontvangen: .................... [x 3 =] ....................

TOEGEWEZEN VERLAGINGSPUNTEN

| Groep ID: | ID codes: | Aantal verlagingspunten: |
|---|---|---|
| PSY-student 1 | | |
| PSY-student 2 | | |
| PSY-student 3 | | |
| PEDA-student 1 | | |
| PEDA-student 2 | | |
| PEDA-student 3 | | |

Totaal aantal punten toegewezen: 60 punten - ....................

.................... - .................... + .................... = ....................

**Figure S28. Example of the feedback sheet (page 1).**

| FASE 1: **6-persoonsgroep** | | | |
|---|---|---|---|
| Groep ID: | ID codes: | Gepakt/gegeven: | Opbrengst fase 1: |
| PSY-student 1 | | | |
| PSY-student 2 | | | |
| PSY-student 3 | | | |
| PEDA-student 1 | | | |
| PEDA-student 2 | | | |
| PEDA-student 3 | | | |
| Inhoud gezamenlijke pot: | | [x 1,5=] | |

| ONTVANGEN VERLAGINGSPUNTEN | | |
|---|---|---|
| Groep ID: | ID codes: | Aantal verlagingspunten: |
| PSY-student 1 | | |
| PSY-student 2 | | |
| PSY-student 3 | | |
| PEDA-student 1 | | |
| PEDA-student 2 | | |
| PEDA-student 3 | | |
| Totaal aantal punten ontvangen: | | [x 3 =] |

| TOEGEWEZEN VERLAGINGSPUNTEN | | |
|---|---|---|
| Groep ID: | ID codes: | Aantal verlagingspunten: |
| PSY-student 1 | | |
| PSY-student 2 | | |
| PSY-student 3 | | |
| PEDA-student 1 | | |
| PEDA-student 2 | | |
| PEDA-student 3 | | |
| Totaal aantal punten toegewezen: | 60 punten | - |

| | | | | | | |
|---|---|---|---|---|---|---|
| | - | | + | | = | |
| Overall totaal: | | | + | | = | |

**Figure S28. Example of the feedback sheet (page 2).**

### 1.3. Statistical Procedures

Here, we describe the statistical modelling strategy for the results reported in the main manuscript. The data of our three experiments were hierarchically structured, because each observation was nested in participants and, in Experiment 1, groups. To account for the dependency of observations, we fitted mixed-effects regression models using the lme4 package in R. To derive *p*-values, we applied the Satterthwaite's method[7], and we used a two-sided *p*-threshold of 5% to determine significance in all models.

*Experiment 1*

To analyse the total group contribution and the total group wealth, we specified separate linear mixed-effects regression models (fitted by maximum likelihood), with a random-effect for groups. To analyse free-riding, the frequency of receiving punishments, and the frequency of punishment, we specified separate generalized linear mixed-effects logistic regression models (fitted by maximum likelihood using the Laplace approximation[8]), with two random-effect intercepts for groups and participants. To analyse the costs of receiving punishments and the expenditure on punishment, we specified separate generalized linear mixed-effects Poisson (logit) regression models (fitted by maximum likelihood using the Laplace approximation[8]), with two random-effect intercepts for participants and groups. In all these models, we included fixed-effect predictors for round and block order to control for their effects.

*Experiments 2 and 3*

To analyse the frequency of third-party punishment, we specified generalized linear mixed-effects logistic regression models (fitted by maximum likelihood using the Laplace approximation[8]), with a random-effect intercept for participants. To analyse the expenditure on third-party punishment, we specified generalized linear mixed-effects Poisson (logit) regression

models (fitted by maximum likelihood using the Laplace approximation[8]), with a random-effect intercept for participants.

For all possible choices in the PGG (by dissimilar others versus similar others), participants made a punishment decision. To determine whether a specific contribution (give-some treatment) or consumption (take-some treatment) can be considered an act of free-riding or cooperation, we took participants own contribution (*consumption*) in the PGG as reference point and coded comparatively lower contributions (*higher consumptions*) by others as free-riding, and contributions equal or above (*consumptions equal or below*) this point as cooperation (for a similar procedure, see[9]). For example, if a participant in the give-some treatment contributed 60 MU in the PGG, we coded a contribution of 0 to 50 MU by the target as free-riding and a contribution of 60 to 100 MU as cooperation. Likewise, if a participant in the take-some treatment consumed 40 MU in the PGG, we coded a consumption of 50 to 100 MU by the target as free-riding and a consumption of 0 to 40 MU as cooperation. To control for the effects of the different contribution-levels (*consumption-levels*) regardless of whether this is coded as free-riding or cooperation, we first reverse-recoded the different consumption-levels in the take-some treatment (to match them with the different contribution-levels in the give-some treatment) and then included a fixed-effect predictor for target's possible contributions/non-consumptions in all our models.

In addition, we also included fixed-effect predictors in all our models that coded whether participants either made decisions in the give-some or take-some treatment, decided about punishing dissimilar and similar others in different orders, and/or were either freshmen or relatively more established students (only in the models for Experiment 2).

## 2. Supplementary Results

For each experiment, we first provide the full models underlying the results reported in the main manuscript and then the additional and/or exploratory analyses that were not the main focus of this research. Finally, we provide sensitivity power analyses for our three experiments.

### 2.1. Experiment 1

#### 2.1.1. Extended Results

*Total group contribution and wealth*

The total contributions of groups were higher with than without punishment in the uniform groups, but significantly less so in the pluriform groups (Table S1, column 1; punishment × group structure interaction coefficient and punishment coefficient). In a similar vein, the total earnings of groups were higher with than without punishment in the uniform groups, but significantly less so in the pluriform groups (Table S1, column 2; punishment × group structure interaction coefficient and punishment coefficient).

*Free-riding*

On the individual-level, free-riding (i.e., when a participant was endowed but did not contribute) was less frequent with than without punishment in the uniform groups, but significantly less so in the pluriform groups (Table S2; punishment × group structure interaction coefficient and punishment coefficient).

**Table S1. Group contribution and wealth as a function of punishment × group structure**

| | (1) | (2) |
|---|---|---|
| Intercept | 47.127*** (4.502) | 96.983*** (4.767) |
| Punishment (0 = absent, 1= present) | 11.333*** (1.325) | 48.044*** (2.037) |
| Group structure (0 = uniform, 1= pluriform) | -0.444 (5.150) | -0.267 (5.395) |
| Round (0 = round 1) | -0.559*** (0.081) | -0.315* (0.125) |
| Block order (0 = without first, 1 = with first) | 2.361 (5.064) | -0.383 (5.199) |
| Punishment × Group structure | -8.278*** (1.873) | -8.811** (2.881) |
| Random intercept variance (group level) | 222.909 | 224.613 |
| Residual | 315.815 | 747.047 |

This table shows the results from the model estimating total group contribution as a function of punishment × group structure (column 1), and the model estimating group wealth as a function of punishment × group structure (column 2). SEs shown in parentheses. *** $P \leq 0.001$, ** $P \leq 0.01$, * $P \leq 0.05$.

**Table S2. Free-riding as a function of punishment × group structure**

| | |
|---|---|
| Intercept | -1.827*** (0.499) |
| Punishment (0 = absent, 1= present) | -1.135*** (0.109) |
| Group structure (0 = uniform, 1= pluriform) | 0.053 (0.570) |
| Round (0 = round 1) | 0.042*** (0.006) |
| Block order (0 = without first, 1 = with first) | -0.138 (0.566) |
| Punishment × Group structure | 0.862*** (0.147) |
| Random intercept variance (individual level) | 2.000 |
| Random intercept variance (group level) | 2.273 |

This table shows the results from the model estimating free-riding (0 = no, 1 = yes) as a function of punishment × group structure. SEs shown in parentheses. *** $P \leq 0.001$, ** $P \leq 0.01$, * $P \leq 0.05$.

*Frequency and costs of receiving punishments*

The differential effects of punishment across the uniform and pluriform groups reported above cannot be explained by the overall frequency and costs of receiving punishments. Participants received punishments from others as frequent in pluriform groups as in uniform groups (Table S3, column 1; group structure coefficient), and the average costs of receiving punishments were also the same (Table S3, column 2; group structure coefficient).

**Table S3. Frequency and costs of punishments received as a function of group structure**

| | (1) | (2) |
|---|---|---|
| Intercept | -0.751 (0.494) | 0.611 (0.440) |
| Group structure (0 = uniform, 1= pluriform) | 0.249 (0.562) | 0.016 (0.505) |
| Round (0 = round 1) | -0.026** (0.008) | -0.013*** (0.002) |
| Block order (0 = without first, 1 = with first) | 0.556 (0.562) | 0.196 (0.505) |
| Random intercept variance (individual level) | 0.275 | 0.339 |
| Random intercept variance (group level) | 2.658 | 2.182 |

This table shows the results from the model estimating the frequency of punishments received (0 = no, 1 = yes) as a function of group structure (column 1), and the model estimating the costs of punishments received as a function of group structure (column 2). SEs shown in parentheses. *** $P \leq 0.001$, ** $P \leq 0.01$, * $P \leq 0.05$.

*Frequency of and expenditure on punishment across uniform and pluriform groups*

Participants punished as frequent in pluriform groups as in uniform groups (Table S4, column 1; group structure coefficient), and mainly directed their punishments at non-contributors rather than contributors (Table S4, column 1; target contributed coefficient). However, the difference in punishment of non-contributors and contributors was overall smaller in the pluriform compared to the uniform groups (Table S4, column 2; group structure × target contributed interaction coefficient; Table S4, columns 3 & 4; target contributed coefficients). Moreover, participants punished more frequently when they themselves had contributed in the current round (Table S4, column 1; source contributed coefficient) and when they had received punishment themselves in the previous round (Table S4, column 1; punishment received t-1 coefficient).

Likewise, participants incurred similar costs to punish in the pluriform group as in the uniform groups (Table S5, column 1; group structure coefficient), and they incurred more costs to punish non-contributors than to punish contributors (Table S5, column 1; target contributed coefficient). The difference in the incurred costs to punish non-contributors and contributors, however, was overall smaller in the pluriform than in the uniform groups (Table S5, column 2; group structure × target contributed interaction coefficient; Table S5, columns 3 & 4; target contributed coefficients). Participants incurred more costs to punish when they themselves had contributed in the current round (Table S5, column 1; source contributed coefficient), and when they had received punishment themselves in the previous round (Table S5, column 1; punishment received t-1 coefficient).

**Table S4. Frequency of punishment as a function of group structure**

| | (1) | (2) | (3) | (4) |
|---|---|---|---|---|
| Intercept | -1.618*** (0.412) | -1.424*** (0.424) | -1.638*** (0.476) | -1.897*** (0.507) |
| Group structure (0 = uniform, 1= pluriform) | -0.295 (0.462) | -0.690 (0.480) | | |
| Target contributed (0 = no, 1 = yes) | -1.710*** (0.079) | -2.163*** (0.114) | -2.155*** (0.114) | 1.254*** (0.111) |
| Source contributed (0 = no, 1 = yes) | 0.577*** (0.083) | 0.597*** (0.083) | 0.624*** (0.122) | 0.558*** (0.115) |
| Punishment received t-1 (mean centred) | 0.082*** (0.017) | 0.086*** (0.017) | 0.087*** (0.023) | 0.086*** (0.026) |
| Round (0 = round 1) | -0.038*** (0.006) | -0.036*** (0.006) | -0.027** (0.009) | -0.045*** (0.009) |
| Block order (0 = without first, 1 = with first) | 0.250 (0.462) | 0.198 (0.474) | 0.462 (0.639) | -0.091 (0.698) |
| Group structure × Target contributed | | 0.925*** (0.158) | | |
| Random intercept variance (individual level) | 4.074 | 4.131 | 3.778 | 4.551 |
| Random intercept variance (group level) | 0.767 | 0.858 | 0.767 | 0.892 |

This table shows the results from the models estimating the frequency of punishment (0 = no, 1 = yes) as a function of group structure (column 1), as a function of group structure × target contributed (column 2), when only including the uniform groups (column 3), and when only including the pluriform groups (column 4). SEs shown in parentheses. *** $P \leq 0.001$, ** $P \leq 0.01$, * $P \leq 0.05$.

**Table S5. Expenditure on punishment as a function of group structure**

| | (1) | (2) | (3) | (4) |
|---|---|---|---|---|
| Intercept | -1.638*** (0.403) | -1.524*** (0.410) | -1.659*** (0.427) | -1.955*** (0.509) |
| Group structure (0 = uniform, 1= pluriform) | -0.314 (0.461) | -0.575 (0.469) | | |
| Target contributed (0 = no, 1 = yes) | -1.008*** (0.037) | -1.307*** (0.051) | -1.303*** (0.051) | -0.675*** (0.052) |
| Source contributed (0 = no, 1 = yes) | 0.427*** (0.040) | 0.443*** (0.040) | 0.425*** (0.059) | 0.463*** (0.054) |
| Punishment received t-1 (mean centred) | 0.042*** (0.007) | 0.045*** (0.007) | 0.041*** (0.097) | 0.049*** (0.010) |
| Round (0 = round 1) | -0.015*** (0.003) | -0.013*** (0.003) | -0.017*** (0.004) | -0.010* (0.004) |
| Block order (0 = without first, 1 = with first) | 0.045 (0.460) | 0.010 (0.468) | 0.508 (0.588) | -0.569 (0.716) |
| Group structure × Target contributed | | 0.625*** (0.073) | | |
| Random intercept variance (individual level) | 3.237 | 3.267 | 2.370 | 4.350 |
| Random intercept variance (group level) | 0.999 | 1.059 | 0.884 | 1.082 |

This table shows the results from the models estimating the expenditure on punishment as a function of group structure (column 1), as a function of group structure × target contributed (column 2), when only including the uniform groups (column 3), and when only including the pluriform groups (column 4). SEs shown in parentheses. *** $P \leq 0.001$, ** $P \leq 0.01$, * $P \leq 0.05$.

*Frequency of and expenditure on punishment in pluriform groups*

Participants punished dissimilar others more frequently than similar others (Table S6, column 1; target's subgroup coefficient), and such discriminatory punishment was unaffected by whether someone had contributed or not (Table S6, column 2; target's subgroup × target contributed interaction coefficient). In a similar vein, participants incurred more costs to punish dissimilar others than similar others (Table S6, column 3; target's subgroup coefficient), and such discriminatory punishment was unaffected by whether someone had contributed or not (Table S6, column 4; target's subgroup × target contributed interaction coefficient).

**Table S6. Frequency of and expenditure on punishment as a function of target's subgroup**

| | (1) | (2) | (3) | (4) |
|---|---|---|---|---|
| Intercept | -2.209*** (0.513) | -2.093*** (0.517) | -2.024*** (0.509) | -2.039*** (0.510) |
| Target's subgroup (0 = similar, 1 = dissimilar) | 0.293** (0.104) | 0.301* (0.134) | 0.111* (0.048) | 0.132* (0.059) |
| Target contributed (0 = no, 1 = yes) | -1.266*** (0.111) | -1.252*** (0.189) | -0.677*** (0.052) | -0.633*** (0.088) |
| Source contributed (0 = no, 1 = yes) | 0.562*** (0.116) | 0.562*** (0.116) | 0.463*** (0.054) | 0.463*** (0.054) |
| Punishment received t-1 (group mean centred) | 0.086*** (0.026) | 0.086*** (0.026) | 0.049*** (0.010) | 0.048*** (0.010) |
| Round (0 = round 1) | -0.045*** (0.009) | -0.045*** (0.009) | -0.010* (0.004) | -0.010* (0.004) |
| Block order (0 = without first, 1 = with first) | -0.090 (0.699) | -0.090 (0.699) | -0.569 (0.715) | -0.570 (0.716) |
| Target's subgroup × Target contributed | | -0.020 (0.220) | | -0.063 (0.10) |
| Random intercept variance (individual level) | 4.582 | 4.583 | 4.352 | 4.355 |
| Random intercept variance (group level) | 0.893 | 0.893 | 1.081 | 1.081 |

This table shows the results from the models estimating the frequency of punishment (0 = no, 1 = yes) as a function of target's subgroup (column 1), and as a function of target's subgroup × target contributed (column 2); the results from the models estimating the expenditure on punishment as a function of target's subgroup (column 3), and as a function of target's subgroup × target contributed (column 4). SEs shown in parentheses. *** $P \leq 0.001$, ** $P \leq 0.01$, * $P \leq 0.05$.

### 2.1.2. Additional and Exploratory Results

*Discriminatory punishers*

To see how many participants punished dissimilar others more than the similar other, and thus engaged in discriminatory punishment, we calculated a difference score for each participant of both their average frequency of punishment and their average expenditure on punishment of similar versus dissimilar others. More specifically, we first calculated, per participant, their average frequency with which they punished similar others across rounds, as well as their average frequency with which they punished dissimilar others across rounds. In a similar vein, we first calculated average expenditure on punishment of similar others and average expenditure on punishment of dissimilar others. For both measures of punishment, we then subtracted, again per participant, their average punishment of similar others from their average punishment of dissimilar others.

These difference scores capture discriminatory punishment (i.e., positive value = they punished the dissimilar others more than the similar other; negative value = they punished the similar other more than the dissimilar others; zero = they punished the similar and dissimilar others equally) and thus allows us to identify whether or not participants, on average, were discriminatory punishers. Figure S29 shows that the majority of participants were indeed discriminatory punishers (62.5% in terms of frequency and 68.1% in terms of expenditure), some were more punitive towards similar others, but most towards dissimilar others.

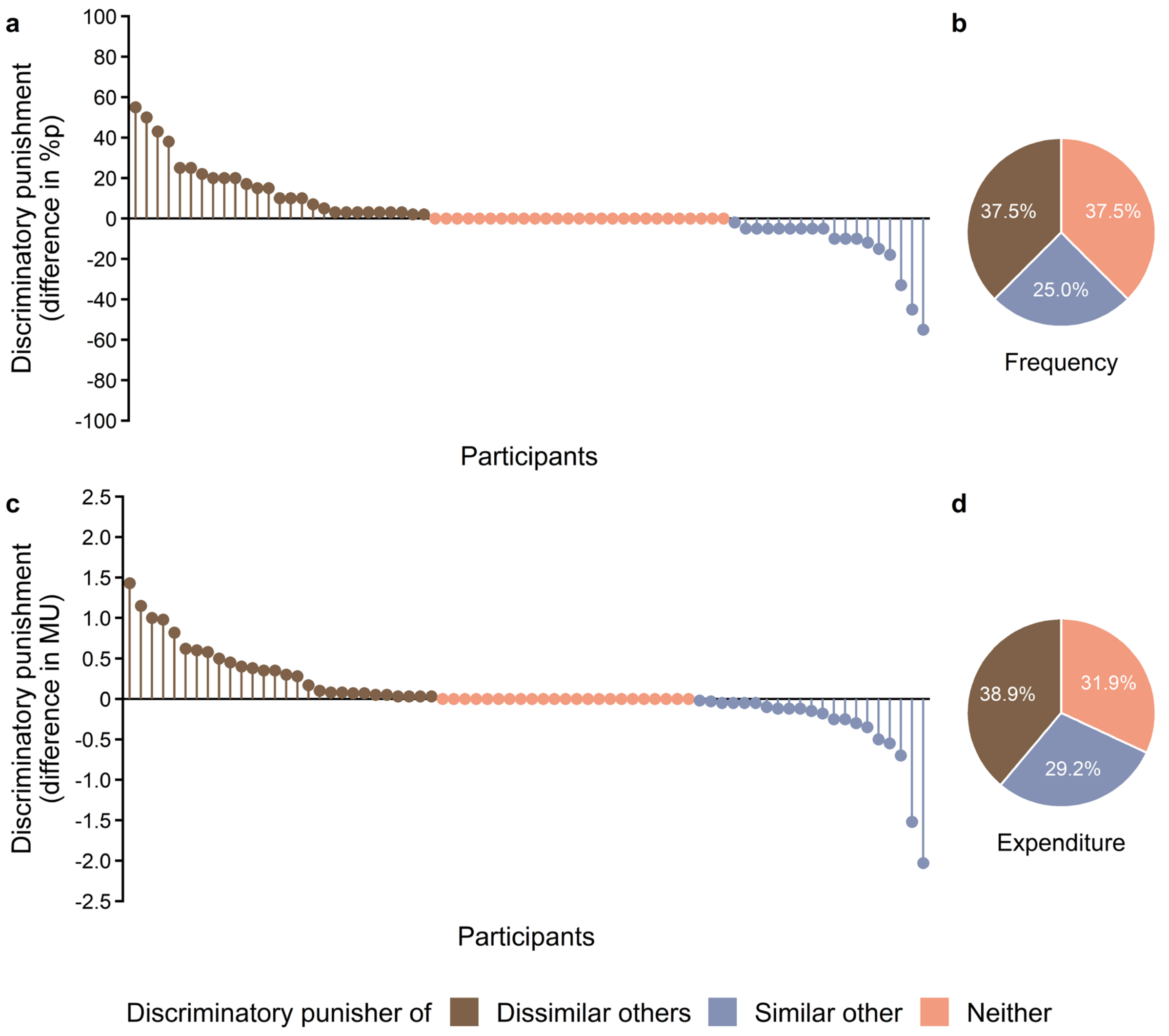


**Figure S29. Discriminatory punishers. (a)** The difference in average frequency of punishment between the dissimilar others and the similar other per participant (i.e., each bar is one participant). **(b)** The proportion of discriminatory punishers in terms of frequency of punishment. **(c)** The difference in the average expenditure on punishment between the dissimilar others and the similar other per participant (i.e., each bar is one participant). **(d)** The proportion of discriminatory punishers in terms of expenditure on punishment.

*Felt affiliation*

For each participant, the experiment always started with an assessment of their felt affiliation with other psychology and pedagogy students, and students from the Faculty of Social and Behavioural Sciences in general. This allowed us to test whether participants felt more affiliated with similar others (i.e., students from their own study programme; e.g., psychology students) than dissimilar others (i.e., students from the other study programme; e.g., pedagogy students) and others from the overarching group in general (i.e., students from the Faculty of Social and Behavioural Sciences in general). We specified a linear mixed-effects regression model (fitted by maximum likelihood), with a random-effect intercept for participants, and two fixed-effect contrasts for dissimilar others (= 1; similar other = 0) and others in general (= 1; similar other = 0). This model showed that participants felt more affiliated with similar others ($M = 3.82$, $SD = 0.79$) than dissimilar others ($M = 1.33$, $SD = 1.13$; $b \pm se = -2.48 \pm 0.09$, $P \leq 0.001$) and others in general ($M = 3.35$, $SD = 0.99$; $b \pm se = -0.47 \pm 0.09$, $P \leq 0.001$).

Next, we calculated a difference score for each participant, capturing their relative affiliation with similar over dissimilar others (i.e., positive value = they felt more affiliated with similar others than with dissimilar others; negative value = they felt less affiliated with similar others than with dissimilar others), and we explored whether this difference in felt affiliation was associated with discriminatory punishment in the pluriform groups. That is, we added the difference score (mean centred) and its interaction with target's subgroup as fixed-effect predictors to the initial models we ran on frequency of punishment and expenditure on punishment in the pluriform groups (for the initial models, see Table S6, columns 1 & 3). These new models both yielded a significant difference score × target's subgroup interaction coefficient (frequency: $b \pm se = 0.19 \pm 0.09$, $P = 0.026$; expenditure: $b \pm se = 0.10 \pm 0.04$, $P = 0.010$). This indicates that participants that felt more affiliated with similar rather than

dissimilar others, also exhibited more discriminatory punishment, both in terms of frequency of punishment and expenditure on punishment.

*Beliefs*

After each block, we assessed participants beliefs about the frequency of free-riding by the other group members in that specific block. This allowed us to test to what extent participants perceived their group members as free-riders, depending on the availability of punishment (absent versus present), the structure of the group (uniform versus pluriform), and the others' subgroup (similar versus dissimilar).

First, for each participant, we calculated the average expected percentage of free-riding in the block with punishment and in the block without punishment. We specified linear mixed-effects regression models (fitted by maximum likelihood), with a random-effect intercept for participants. In the first model, we included two fixed-effect predictors for punishment (0 = absent; 1 = present) and group structure (0 = uniform; 1 = pluriform), as well as a fixed-effect predictor for block order (0 = without punishment first; 1 = with punishment first) to control for its effects. In the second model, we also included a fixed-effect predictor for the punishment × group structure interaction. These models yielded a significant punishment coefficient, indicating that participants believed that their group members were free-riding less frequently with punishment ($M_{\%} = 36.78$, $SE_{\%} = 1.33$) than without punishment ($M_{\%} = 41.80$, $SE_{\%} = 1.33$), $b \pm se = -5.02 \pm 1.88$, $P = 0.008$. The group structure coefficient ($b \pm se = 4.73 \pm 4.45$, $P = 0.290$) and the punishment × group structure interaction coefficient ($b \pm se = 4.76 \pm 3.73$, $P = 0.204$) were both non-significant.

Second, for each participant in the pluriform group, we calculated the average expected percentage of free-riding by the one similar other and two dissimilar others in their pluriform group. We specified linear mixed-effects regression models (fitted by maximum likelihood),

with a random-effect intercept for participants. In the first model, we included two fixed-effect predictors for punishment (0 = absent; 1 = present) and target's subgroup (0 = similar; 1 = dissimilar), as well as a fixed-effect predictor for block order (0 = without punishment first; 1 = with punishment first) to control for its effects. In the second model, we also included a fixed-effect predictor for the punishment × target's subgroup interaction. The coefficients of punishment ($b \pm se$ = -2.40 ± 2.08, $P$ = 0.250), target's subgroup ($b \pm se$ = 1.00 ± 2.08, $P$ = 0.630), and the punishment × target's subgroup interaction ($b \pm se$ = 1.46 ± 4.15, $P$ = 0.726) were all non-significant.

Combined, these analyses suggest that our introduction of a pluriform group structure did not impact participants' beliefs about the frequency of free-riding by others in their group. Thus, although we observed discriminatory punishment in the pluriform groups, such subgroup-based discrimination may not be rooted in different beliefs about group members.

*Social value orientation*

For each participant, the experiment ended with an assessment of their social value orientation (SVO), which allowed us to check whether social preferences were comparable across uniform and pluriform groups. Figure S30 shows, for each group, the average deviation of group members' SVO score from the pre-determined boundary between the categories prosocial and individualistic in the SVO task (SVO score = 22.45)[3]. As can be seen, the majority of the groups were, on average, prosocially rather than individualistically oriented. More importantly, SVO scores were similar in uniform and pluriform groups. A linear regression model showed that participants' SVO scores were not significantly different between uniform groups ($M$ = 25.48, $SD$ = 18.48) and pluriform groups ($M$ = 25.05, $SD$ = 18.65), $b \pm se$ = -0.42 ± 2.57, $P$ = 0.869.

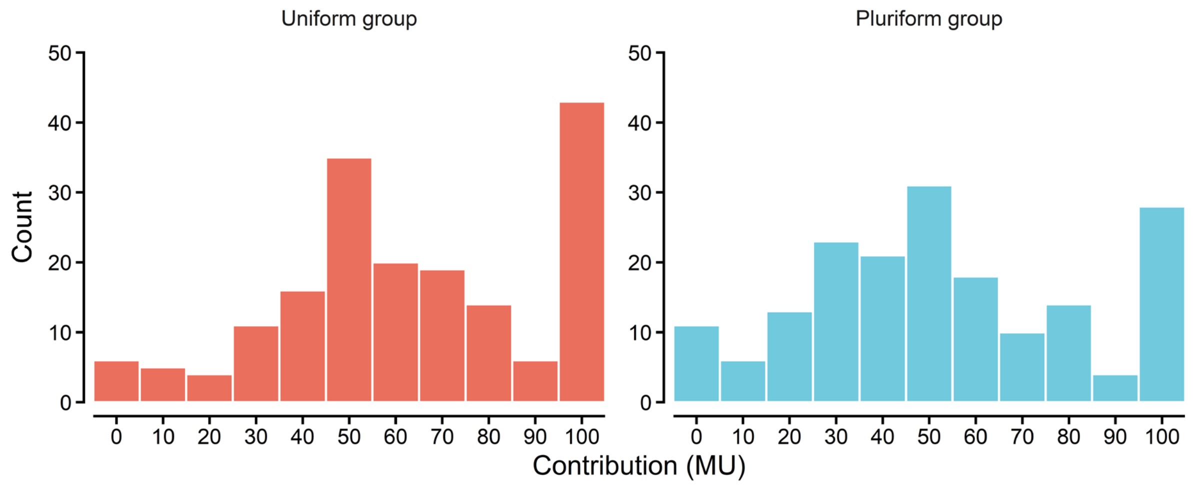


**Figure S30. Average SVO score within uniform and pluriform groups.** For each uniform or pluriform group (i.e., each bar is one group), the average deviation of group members' SVO score from the boundary between prosociality (i.e., SVO score ≥ 22.45) and individualism (i.e., SVO score < 22.45). Error bars indicate the standard error of the mean deviation.

Next, we explored whether discriminatory punishment emerged even when controlling for SVO, and whether SVO was associated with the emergence of discriminatory punishment. Therefore, we first added the SVO score (mean centred) and, secondly, also its interaction with target's subgroup as fixed-effect predictors to the initial models we ran on frequency of punishment and expenditure on punishment in the pluriform groups (for the initial models, see Table S6, columns 1 & 3). These additional models, first of all, showed that the target's subgroup coefficient remained significant (frequency: $b \pm se = 0.29 \pm 0.10$, $P = 0.005$; expenditure: $b \pm se = 0.11 \pm 0.05$, $P = 0.020$) when controlling for SVO score (frequency: $b \pm se = -0.02 \pm 0.02$, $P = 0.343$; expenditure: $b \pm se = -0.01 \pm 0.02$, $P = 0.441$). Secondly, the new model for the frequency of punishment yielded a non-significant SVO score × target's subgroup interaction coefficient ($b \pm se = 0.01 \pm 0.01$, $P = 0.112$). In a similar vein, the new model for the expenditure on punishment also yielded a non-significant SVO score × target's subgroup

interaction coefficient ($b \pm se = 0.01 \pm 0.003$, $P = 0.057$), but did suggest a trend that especially those with high SVO scores may, on average, spend more on punishing a dissimilar other than a similar other. However, including the SVO score × target's subgroup interaction coefficient in the models did not alter the significance of the target's subgroup coefficient (frequency: $b \pm se = 0.32 \pm 0.11$, $P = 0.003$; expenditure: $b \pm se = 0.15 \pm 0.05$, $P = 0.004$), indicating that we observed discriminatory punishment, irrespective of participants' SVO.

## 2.2. Experiment 2

### 2.2.1. Extended Results

*Frequency of and expenditure on TP punishment*

Like in Experiment 1, we again found that participants mainly directed their punishments at free-riders rather than cooperators (Table S7, column 1; target a free-rider coefficient), and incurred more costs to punish these free-riders (Table S7, column 3; target a free-rider coefficient). Moreover, participants' own contribution level was associated with both the frequency of punishment (Table S7, column 1; source's contribution coefficient) and the expenditure on punishment (Table S7, column 3; source's contribution coefficient), indicating that high contributors punished more than low contributors (note that the consumptions in the take-some treatment were reverse-coded; see explanation below under *Contribution*).

Crucially, and complementing Experiment 1, participants punished dissimilar others more frequently than similar others (Table S7, column 1; target's subgroup coefficient), irrespective of whether the target was free-riding or cooperating (Table S7, column 2; target's subgroup × target a free-rider interaction coefficient). Likewise, participants incurred more costs to punish dissimilar others than dissimilar others (Table S7, column 3; target's subgroup coefficient), irrespective of whether the target was free-riding or cooperating (Table S7, column 4; target's subgroup × target a free-rider interaction coefficient).

**Table S7. Frequency of and expenditure on punishment as a function of target's subgroup**

| | (1) | (2) | (3) | (4) |
|---|---|---|---|---|
| Intercept | 0.113 (0.647) | 0.093 (0.650) | -0.217 (0.286) | -0.226 (0.287) |
| Target's subgroup (0 = similar, 1 = dissimilar) | 0.397*** (0.106) | 0.435** (0.159) | 0.078*** (0.020) | 0.095* (0.040) |
| Target a free-rider (0 = no, 1 = yes) | 1.615*** (0.219) | 1.651*** (0.245) | 0.147*** (0.039) | 0.159*** (0.045) |
| Target's contribution | -0.722*** (0.035) | -0.722*** (0.035) | -0.243*** (0.005) | -0.243*** (0.005) |
| Source's contribution (mean centred) | -0.191 (0.111) | -0.191 (0.111) | -0.058 (0.151) | -0.058 (0.151) |
| Wave (0 = sem1, 1 = sem2) | -1.720* (0.673) | -1.720* (0.673) | -0.904** (0.311) | -0.904** (0.311) |
| First subgroup (0 = dissimilar, 1 = similar) | 0.216 (0.633) | 0.216 (0.633) | 0.277 (0.297) | 0.277 (0.297) |
| Treatment (0 = take-some, 1 = give-some) | 0.550 (0.634) | 0.550 (0.634) | 0.321 (0.297) | 0.321 (0.297) |
| Target's subgroup × Target a free-rider | | -0.070 (0.212) | | -0.023 (0.047) |
| Random intercept variance (individual level) | 23.664 | 23.663 | 5.610 | 5.610 |

This table shows the results from the models estimating the frequency of punishment (0 = no, 1 = yes) as a function of target's subgroup (column 1), and as a function of target's subgroup × target a free-rider (column 2); the results from the models estimating the expenditure on punishment as a function of target's subgroup (column 3), and as a function of target's subgroup × target a free-rider (column 4). SEs shown in parentheses. *** $P \leq 0.001$, ** $P \leq 0.01$, * $P \leq 0.05$.

### 2.2.2. Additional and Exploratory Results

*Discriminatory punishers*

Like in Experiment 1, we again calculated a difference score for each participant of both their average frequency of punishment and their average expenditure on punishment to identify how many participants engaged in discriminatory punishment. In Experiment 2, participants specified punishment strategies (rather than punishing across rounds as was the case in Experiment 1), and we subtracted the average frequency (expenditure) with which each participant punished similar others from the average frequency (expenditure) with which they punished dissimilar others across all possible contributions. Hence, this difference score also captures discriminatory punishment (i.e., positive value = they punished dissimilar others more than similar others; negative value = they punished similar others more than dissimilar others; zero = they punished the similar and dissimilar others equally) and again allows us to identify whether or not participants, on average, were discriminatory punishers.

Figure S31 shows that, in contrast to Experiment 1, the majority of participants punished dissimilar others equally to similar others and, thus, were not discriminatory punishers. Of the participants who were discriminatory punishers (23.6% in terms of frequency and 34.4% in terms of expenditure), most of them directed this towards dissimilar rather than similar others. Whereas the difference scores in Experiment 1 were calculated based on participants' average punishment across rounds in the repeated interaction, the difference scores in Experiment 2 were calculated based on participants' average punishment across all possible contributions in the one-shot interaction. This difference, together with the fact that participants were third parties overseeing the public good provision of another pluriform group without being subject to noise about others' intentions, may explain the difference in results between Experiments 1 and 2.

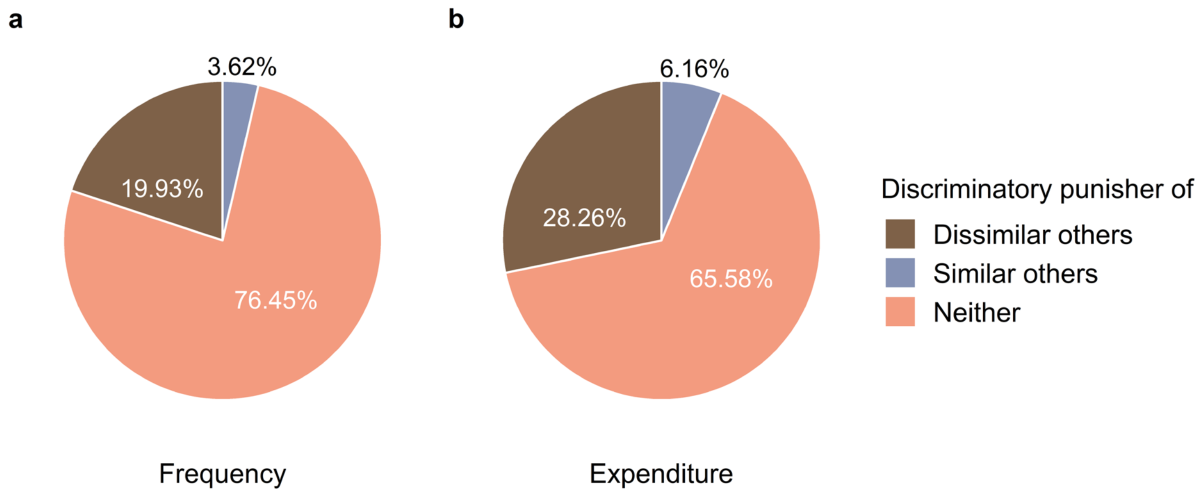


**Figure S31. The proportion of discriminatory punishers. (a)** In terms of frequency of punishment. **(b)** In terms of expenditure on punishment.

*Change in punishment strategy for alternative group compositions*

Participants were asked whether and how they wanted to change their punishment strategies if the composition would not be 3 psychology and 3 pedagogy students, but either 4 psychology students and 2 pedagogy students (i.e., majority of psychology students) or vice versa (i.e., majority of pedagogy students). This allowed us to see whether the observed patterns of discriminatory punishment would change when dissimilar others would either become a majority or minority in the pluriform group. To analyse participants' punishment strategies across the three group compositions (i.e., equal, dissimilar majority, dissimilar minority), we extended the initial models we ran on frequency of punishment and expenditure on punishment by including fixed-effect contrasts for dissimilar majority (= 1; equal = 0) and dissimilar minority (= 1; equal = 0), as well as their interactions with target's subgroup.

Interestingly, these additional models showed that when dissimilar others would become a majority, the difference in the expenditure on punishment between dissimilar others and similar

others became larger (dissimilar majority × target's subgroup interaction; $b \pm se = 0.06 \pm 0.03$, $P = 0.038$), but not the difference in the frequency of punishment (dissimilar majority × target's subgroup interaction; $b \pm se = 0.16 \pm 0.15$, $P = 0.298$). When dissimilar others would become a minority, by contrast, both the difference in frequency of punishment (dissimilar minority × target's subgroup interaction; $b \pm se = -0.06 \pm 0.15$, $P = 0.673$) and expenditure on punishment (dissimilar majority × target's subgroup interaction; $b \pm se = 0.02 \pm 0.03$, $P = 0.614$) remained the same. Irrespective of group composition, dissimilar others were punished more than similar others, both in terms of frequency of punishment ($b \pm se = 0.44 \pm 0.06$, $P \leq 0.001$) and expenditure on punishment ($b \pm se = 0.10 \pm 0.01$, $P \leq 0.001$).

*Contribution*

Before participants specified their punishment strategies, they had first made a contribution decision (in the give-some treatment) or consumption decision (in the take-some treatment) themselves. To include this contribution/consumption decision as predictor in the above models, we reverse-recoded the different consumption-levels in the take-some treatment to match them with the different contribution-levels in the give-some treatment. For example, the consumption of 40 MU equalled a contribution of 60 MU and was, therefore, reverse-coded to a non-consumption of 60 MU. We collapsed these decisions across treatments and refer to them as contributions in the results. Figure S32 shows the frequency of contributions. Participants, on average, contributed 55.36 MU ($SD = 29.95$) in the PGG.

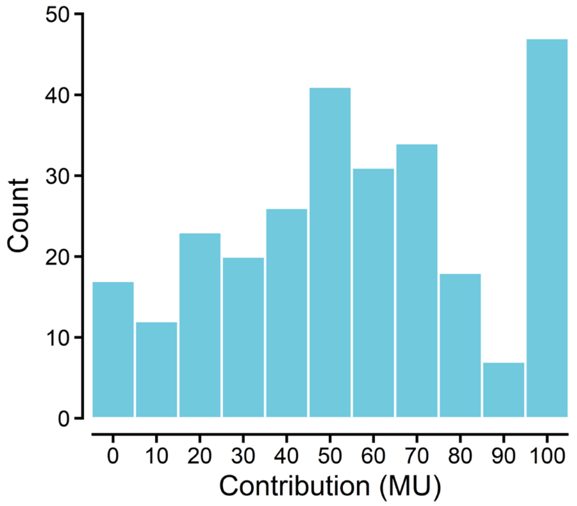


**Figure S32. The frequency of contributions.**

*Felt affiliation*

As in Experiment 1, Experiment 2 always started with an assessment of participants felt affiliation with other psychology and pedagogy students. We specified a linear mixed-effects regression model (fitted by maximum likelihood), with a random-effect intercept for participants, and a fixed-effect contrast (0 = similar other; 1 = dissimilar other). This model showed that participants felt more affiliated with similar others ($M$ = 5.32, $SD$ = 0.96) than dissimilar others ($M$ = 2.69, $SD$ = 1.10), $b \pm se$ = -2.63 ± 0.08, $P \leq 0.001$.

Next, we calculated a difference score for each participant, capturing their felt affiliation with similar others over dissimilar others (i.e., positive value = they felt more affiliated with similar others than with dissimilar others; negative value = they felt less affiliated with similar others than with dissimilar others), and we added this difference score (mean centred) and its interaction with target's subgroup as fixed-effect predictors to the initial models we ran on frequency of punishment and expenditure on punishment in the pluriform groups (for the initial models, see Table S7, columns 1 & 3). Like Experiment 1, this model for expenditure on

punishment yielded a significant difference score × target's subgroup interaction coefficient ($b \pm se = 0.06 \pm 0.02$, $P \leq 0.001$), which was not the case for frequency of punishment ($b \pm se = 0.13 \pm 0.08$, $P = 0.110$). This indicates that participants displayed more discriminatory punishment (in terms of the costs they incurred to punish) the more affiliated they felt with similar others rather than dissimilar others.

In contrast to our first experiment, participants in this second experiment where third parties overseeing the one-shot public good provision of another pluriform group without being subject to noise about others' intentions. One or more of these differences in experimental design may explain why the difference in felt affiliation between dissimilar and similar others was positively associated with the degree of discriminatory punishment in terms of incurred costs but not in terms of frequency.

*General trust, felt threat, and perceptions*

Throughout the experiment, we assessed participants' perceptions of other students in the study programmes Psychology and Pedagogical Science. More specifically, after participants were instructed about the PGG they faced, we assessed their general trust that other psychology and pedagogy students would serve the collective interest, and how threatened the involvement of other psychology and pedagogy students made them feel. Moreover, at the end of the experiment, we assessed some general positive and negative perceptions of other psychology and pedagogy students. These measures allowed us to assess whether participants had differential perceptions about similar and dissimilar others.

For each measure (i.e., general trust, felt threat, positive perceptions, and negative perceptions), we specified a linear mixed-effects regression model (fitted by maximum likelihood), with a random-effect intercept for participants, and a fixed-effect contrast (0 = similar other; 1 = dissimilar other). These models showed that participants generally trusted dissimilar others (*M*

= 4.82, *SD* = 0.92) to the same degree as similar others (*M* = 4.80, *SD* = 0.93; $b \pm se = 0.02 \pm 0.04$, $P = 0.680$), felt equally threatened by dissimilar others (*M* = 2.76, *SD* = 1.42) and similar others (*M* = 2.76, *SD* = 1.44; $b \pm se = 0.01 \pm 0.05$, $P = 0.878$), and had comparable level of negative perceptions of dissimilar others (*M* = 2.41, *SD* = 0.95) and similar others (*M* = 2.39, *SD* = 0.94; $b \pm se = 0.02 \pm 0.04$, $P = 0.607$). Participants did have lower positive perceptions of dissimilar others (*M* = 4.79, *SD* = 0.73) than of similar others (*M* = 5.17, *SD* = 0.74; $b \pm se = -0.38 \pm 0.03$, $P \leq 0.001$). Taken together, however, these measures suggest that participants did not have strong differential perceptions of similar and dissimilar others.

### 2.3. Experiment 3

#### 2.3.1. Extended Results

*Frequency of and expenditure on TP punishment*

Regardless of whether others were in a uniform or pluriform group, participants mainly directed their punishments at free-riders rather than cooperators (Table S8, column 1; target a free-rider coefficient), and incurred more costs to punish these free-riders (Table S9, column 1; target a free-rider coefficient). Moreover, participants own contribution level was associated with the expenditure on punishment of others in the uniform and pluriform groups (Table S9, column 3; source's contribution coefficient), but not with the frequency with which participants punished others in the uniform and pluriform groups (Table S8, column 1; source's contribution coefficient), which indicates that high contributors incurred more costs to punish others in the uniform and pluriform groups than low contributors (as in Experiment 2, consumptions in the take-some treatment were reverse-coded).

Participants punished dissimilar others more frequently than similar others (Table S8, column 1; target's subgroup coefficient), and they incurred more costs to punish them (Table S9, column 3; target's subgroup coefficient), irrespective of whether the other was free-riding or cooperating (frequency: Table S8, column 2; target's subgroup × target a free-rider interaction coefficient; expenditure: Table S9, column 2; target's subgroup × target a free-rider interaction coefficient). Interestingly, however, the effects of target's subgroup were dependent on whether the target was in a pluriform or uniform group (frequency: Table S8, column 2; target's subgroup × group structure interaction coefficient; expenditure: Table S9, column 2; target's subgroup × group structure interaction coefficient). Replicating the results of Experiments 1 and 2, participants punished dissimilar others rather than similar others in the pluriform group more frequently (Table S8, column 4; target's subgroup coefficient) and incurred more costs to

punish them (Table S9, column 4; target's subgroup coefficient). When psychology and pedagogy students were each in separate uniform groups, however, participants punished dissimilar and similar others as frequent (Table S8, column 3; target's subgroup coefficient), but did incur more costs on punishing dissimilar others (Table S9, column 3; target's subgroup coefficient).

**Table S8. Frequency of punishment as a function of target's subgroup**

| | (1) | (2) | (3) | (4) |
|---|---|---|---|---|
| Intercept | 0.797 (0.564) | 0.851 (0.570) | 0.870 (0.601) | 0.802 (0.625) |
| Target's subgroup (0 = similar, 1 = dissimilar) | 0.261** (0.081) | 0.158 (0.154) | 0.028 (0.118) | 0.516*** (0.119) |
| Target a free-rider (0 = no, 1 = yes) | 0.839*** (0.149) | 0.948*** (0.169) | 1.225*** (0.237) | 0.856*** (0.220) |
| Group structure (0 = uniform, 1 = pluriform) | 0.067 (0.092) | -0.160 (0.123) | | |
| Target's contribution | -0.685*** (0.026) | -0.686*** (0.026) | -0.716*** (0.039) | -0.689*** (0.038) |
| Source's contribution (mean centred) | 0.059 (0.042) | 0.059 (0.042) | -0.158 (0.116) | -0.197 (0.116) |
| First subgroup (0 = dissimilar, 1 = similar) | 0.442 (0.617) | 0.444 (0.619) | 0.647 (0.624) | 0.368 (0.648) |
| Treatment (0 = take-some, 1 = give-some) | 0.246 (0.617) | 0.246 (0.619) | 0.343 (0.620) | -0.063 (0.646) |
| Target's subgroup × Target a free-rider | | -0.209 (0.164) | | |
| Target's subgroup × Group structure | | 0.450** (0.162) | | |
| Random intercept variance (individual level) | 16.282 | 16.362 | 15.569 | 16.627 |

This table shows the results from the models estimating the frequency of punishment (0 = no, 1 = yes) as a function of target's subgroup (column 1), and as a function of target's subgroup × target a free-rider (column 2), when only including the uniform groups (column 3), and when only including the pluriform groups (column 4). SEs shown in parentheses. *** $P \leq 0.001$, ** $P \leq 0.01$, * $P \leq 0.05$.

**Table S9. Expenditure on punishment as a function of target's subgroup**

| | (1) | (2) | (3) | (4) |
|---|---|---|---|---|
| Intercept | -0.239 (0.301) | -0.226 (0.301) | -0.019 (0.262) | -0.184 (0.290) |
| Target's subgroup (0 = similar, 1 = dissimilar) | 0.119*** (0.017) | 0.092* (0.042) | 0.058* (0.024) | 0.181*** (0.024) |
| Target a free-rider (0 = no, 1 = yes) | 0.223*** (0.033) | 0.245*** (0.040) | 0.260*** (0.049) | 0.213*** (0.046) |
| Group structure (0 = uniform, 1 = pluriform) | 0.059** (0.020) | -0.004 (0.027) | | |
| Target's contribution | -0.241*** (0.004) | -0.241*** (0.005) | -0.248*** (0.006) | -0.232*** (0.006) |
| Source's contribution (mean centred) | 0.044*** (0.009) | 0.044*** (0.009) | -0.024 (0.054) | -0.047 (0.057) |
| First subgroup (0 = dissimilar, 1 = similar) | 0.213 (0.342) | 0.213 (0.342) | 0.230 (0.294) | 0.157 (0.324) |
| Treatment (0 = take-some, 1 = give-some) | 0.412 (0.342) | 0.412 (0.342) | 0.411 (0.293) | 0.294 (0.323) |
| Target's subgroup × Target a free-rider | | -0.041 (0.042) | | |
| Target's subgroup × Group structure | | 0.119*** (0.034) | | |
| Random intercept variance (individual level) | 4.978 | 4.978 | 3.603 | 4.369 |

This table shows the results from the models estimating the expenditure on punishment as a function of target's subgroup (column 1), and as a function of target's subgroup × target a free-rider (column 2), when only including the uniform groups (column 3), and when only including the pluriform groups (column 4). SEs shown in parentheses. *** $P \leq 0.001$, ** $P \leq 0.01$, * $P \leq 0.05$.

### 2.3.2. Additional and Exploratory Results

*Discriminatory punishers*

Similar to Experiments 1 and 2, we again calculated a difference score for each participant of both their average frequency of punishment and their average expenditure on punishment to identify how many participants engaged in discriminatory punishment. However, in Experiment 3, participants specified punishment strategies for uniform and pluriform groups. Separately for the uniform groups and the pluriform group, we therefore subtracted the average frequency (expenditure) with which each participant punished similar others from the average frequency (expenditure) with which they punished dissimilar others across all possible contributions. Hence, these difference scores both capture discriminatory punishment (i.e., positive value = they punished dissimilar others more than similar others; negative value = they punished similar others more than dissimilar others; zero = they punished the similar and dissimilar others equally) and again allow us to identify whether or not participants, on average, were discriminatory punishers and whether this differed between the uniform groups and the pluriform group.

Complementing Experiment 2, Figure S33 shows that the majority of participants punished dissimilar others equally to similar others in both uniform and pluriform groups and, thus, never were discriminatory punishers. Of the participants who were discriminatory punishers towards dissimilar others (31.8% in terms of frequency and 44.1% in terms of expenditure), most of them either were so in both the uniform and pluriform groups or, more importantly, only in the pluriform group.

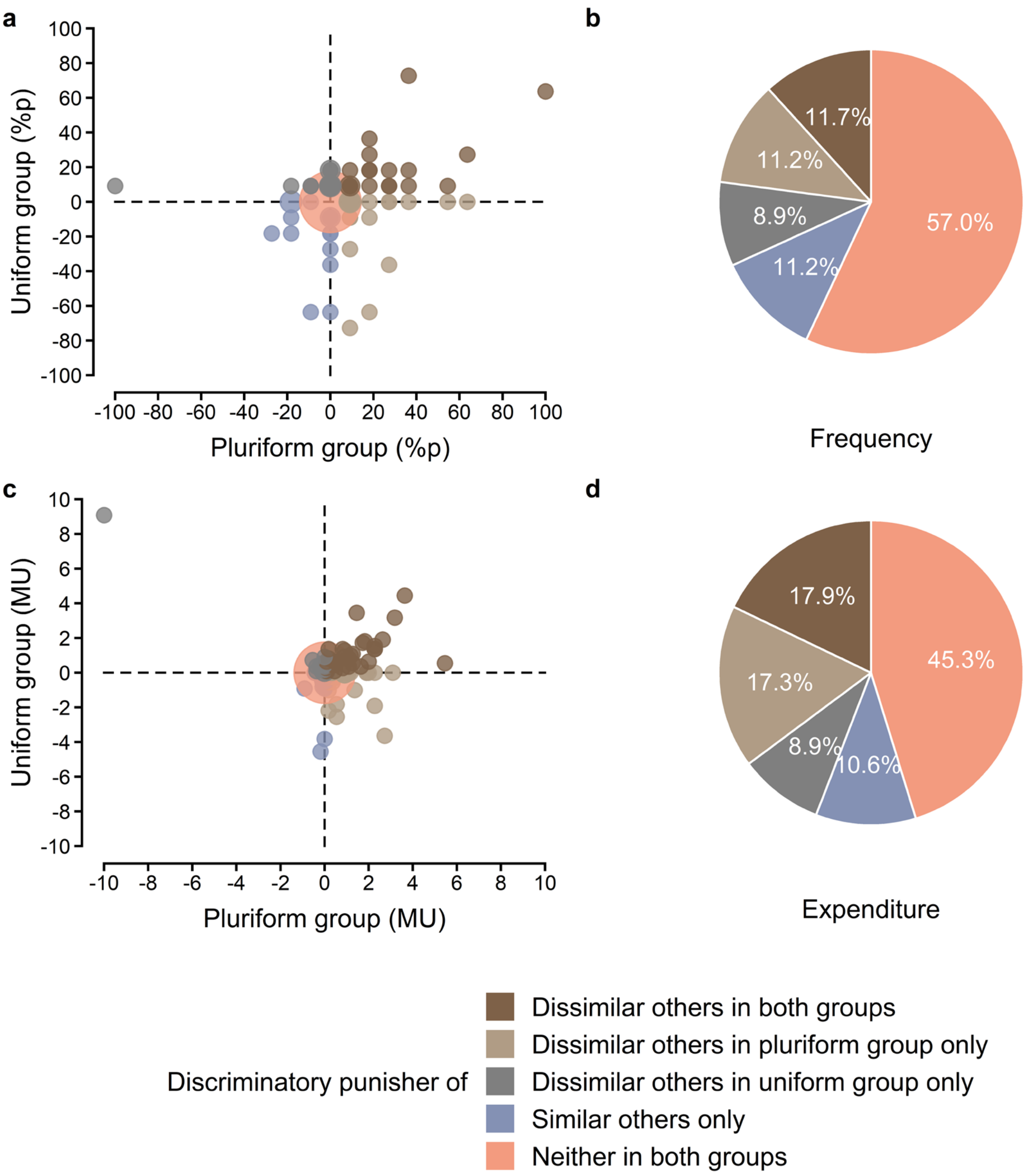


**Figure S33. Discriminatory punishers in uniform and pluriform groups. (a)** The difference in average frequency of punishment between dissimilar others and similar others per participant (i.e., each dot is one participant), as a function of group structure. **(b)** The proportion of discriminatory punishers in uniform and pluriform groups in terms of frequency of punishment. **(c)** The difference in the average expenditure on punishment between dissimilar others and similar others per participant (i.e., each dot is one participant), as a function of group structure. **(d)** The proportion of discriminatory punishers in uniform and pluriform groups in terms of expenditure on punishment.

*Contributions*

Before participants specified their punishment strategies, they had first made contribution decisions (in the give-some treatment) or consumption decisions (in the take-some treatment) themselves. Similar to Experiment 2, we reverse-coded the consumption-levels in the take-some treatment to match them with the contribution-levels in the give-some treatment, and collapsed participants' decisions across treatments. We refer to these collapsed decisions as contributions in the results. Figure S34 shows the frequency of contributions in the uniform groups and the pluriform groups. Although the underlying outcome structure of the PGG in the uniform group was not exactly the same as in the pluriform group (due to the difference in group size), we tested for differences in contributions across the two groups. We specified a linear mixed-effects regression model (fitted by maximum likelihood), with a random-effect intercept for participants, and a fixed-effect predictor for group structure (0 = uniform group; 1 = pluriform group). This model showed that participants contributed more to the group account in the uniform group ($M$ = 63.35 $SD$ = 28.04) than in the pluriform group ($M$ = 52.85, $SD$ = 29.36), $b \pm se$ = -1.05 ± 0.14, $P \leq 0.001$.

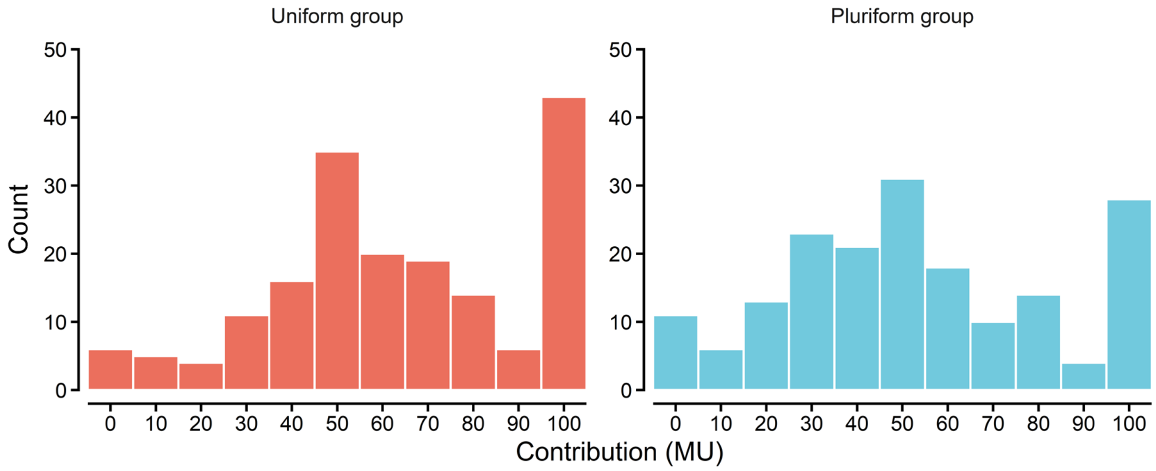


**Figure S34. The frequency of contributions.**

*Felt affiliation*

Like Experiments 1 and 2, Experiment 3 always started with an assessment of participants felt affiliation with other psychology and pedagogy students. We specified a linear mixed-effects regression model (fitted by maximum likelihood), with a random-effect intercept for participants, and a fixed-effect contrast (0 = similar other; 1 = dissimilar other). This model showed that participants felt more affiliated with similar others ($M$ = 5.43 $SD$ = 0.91) than dissimilar others ($M$ = 2.78, $SD$ = 1.18), $b \pm se$ = -2.65 ± 0.09, $P \leq 0.001$.

Next, we again calculated a difference score for each participant, capturing their felt affiliation with similar over dissimilar others (i.e., positive value = they felt more affiliated with similar than dissimilar others; negative value = they felt less affiliated with similar than dissimilar others). We added this difference score (mean centred) and its interaction with target's subgroup and group structure as fixed-effect predictors to the initial models we ran on frequency of punishment and expenditure on punishment (for the initial models, see Tables S8 & S9, columns 2, 3 and 4). This model for expenditure yielded a significant difference score × target's subgroup × group structure interaction coefficient ($b \pm se$ = -0.05 ± 0.03, $P$ = 0.048), which was not the case for frequency of punishment ($b \pm se$ = -0.10 ± 0.14, $P$ = 0.477). Thus, complementing Experiment 2, participants displayed more discriminatory punishment (in terms of the costs they incurred to punish) in pluriform rather than uniform groups, the more affiliated they felt with similar others rather than dissimilar others.

*General trust, felt threat, and perceptions*

Throughout the experiment, we assessed participants' perceptions of other students in the study programmes Psychology and Pedagogical Science to assess whether participants had differential perceptions about similar and dissimilar others. For each measure (i.e., general trust, felt threat, positive perceptions, and negative perceptions), we specified a linear mixed-effects

regression model (fitted by maximum likelihood), with a random-effect intercept for participants, and a fixed-effect contrast (0 = similar other; 1 = dissimilar other).

As in Experiment 2, these models showed that participants generally trusted dissimilar others ($M = 4.81$, $SD = 0.91$) to the same degree as similar others ($M = 4.87$, $SD = 0.82$; $b \pm se = -0.07 \pm 0.05$, $P = 0.220$), and felt equally threatened by dissimilar others ($M = 3.15$, $SD = 1.57$) and similar others ($M = 3.04$, $SD = 1.57$; $b \pm se = 0.11 \pm 0.07$, $P = 0.097$). However, participants did have lower positive perceptions of dissimilar others ($M = 4.77$, $SD = 0.78$) than of similar others ($M = 5.18$, $SD = 0.69$; $b \pm se = -0.40 \pm 0.04$, $P \leq 0.001$) and higher negative perceptions of dissimilar others ($M = 2.35$, $SD = 0.97$) than of similar others ($M = 2.25$, $SD = 0.95$; $b \pm se = 0.10 \pm 0.05$, $P = 0.027$). Thus, in terms of general perceptions (measured after participants performed the PGG), participants seemed to have differential perceptions of similar and dissimilar others, but not in terms of general trust that the others would serve the collective interest and how threatened these others made them feel.

### 2.4. Sensitivity Analyses

In our experiments, sample size was determined based on feasibility concerns rather than a priori power calculations (see *Supplementary Methods*). We conducted sensitivity power analyses to determine the minimum effect size that could be detected with a power of .80 in our mixed-effects regression models of the key dependent variables. For these estimated models, we substituted the coefficient of interest with a range of coefficients, and on each of these coefficients, we conducted 500 simulated power analyses using the simr package in R[10]. In each simulation, new values for the response variable were simulated using the specified model, the model (with the substituted coefficient) was then refitted to the simulated response, and a statistical test was applied to the simulated fit. Power was calculated from the number of positive and negative runs.

*Experiment 1*

First, we took the model estimating total group contribution (Table S1, column 1) and substituted the punishment × group structure interaction coefficient with coefficients ranging from $b$ = -3 through $b$ = -7. Second, we also took the model estimating frequency of punishment in the pluriform groups (Table S6, column 1) and substituted the target's subgroup coefficient with coefficients ranging from $b$ = 0.2 through $b$ = 0.4. Early simulation runs showed that the upper limit of $b$ = -7 and $b$ = 0.4 were sufficient to approach a statistical power of 1. Results are depicted in Figure S35. The minimum effect size to be detected with a power of .80 for the punishment × group structure interaction in the model estimating total group contribution was a coefficient of approximately -5.19 (the observed coefficient was -8.278). For the effect of target's subgroup in the model estimating frequency of punishment in the pluriform groups, the minimum effect size to be detected with a power of .80 was a coefficient of approximately 0.30 (the observed coefficient was 0.293).

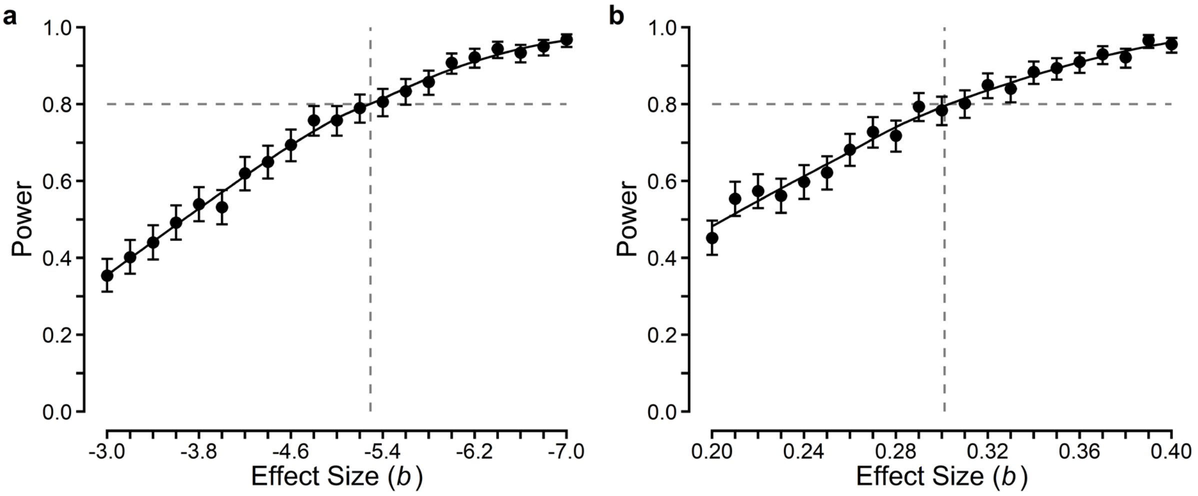


**Figure S35. Statistical power for a range of effect sizes. (a)** For the model of total group contribution. **(b)** For the model of frequency of punishment.

*Experiments 2 and 3*

For Experiment 2, we took the model estimating frequency of punishment (Table S7, column 1), and substituted the target's subgroup coefficient with coefficients ranging from $b$ = 0.1 through $b$ = 0.4. For Experiment 3, we also took the model estimating frequency of punishment (Table S8, column 2), but substituted the target's subgroup × group structure interaction coefficient with coefficients ranging from $b$ = 0.2 through $b$ = 0.6. Early simulation runs showed that the upper limit of $b$ = 0.4 and $b$ = 0.6, respectively, were sufficient to approach a statistical power of 1. Results are depicted in Figure S36. The minimum effect size to be detected with a power of .80 for the effect of target's subgroup in the model estimating frequency of punishment in Experiment 2 was a coefficient of approximately 0.29 (the observed coefficient was 0.397). For the target's subgroup × group structure interaction in the model estimating frequency of punishment in Experiment 3, the minimum effect size to be detected with a power of .80 was a coefficient of approximately 0.46 (the observed coefficient was 0.450).

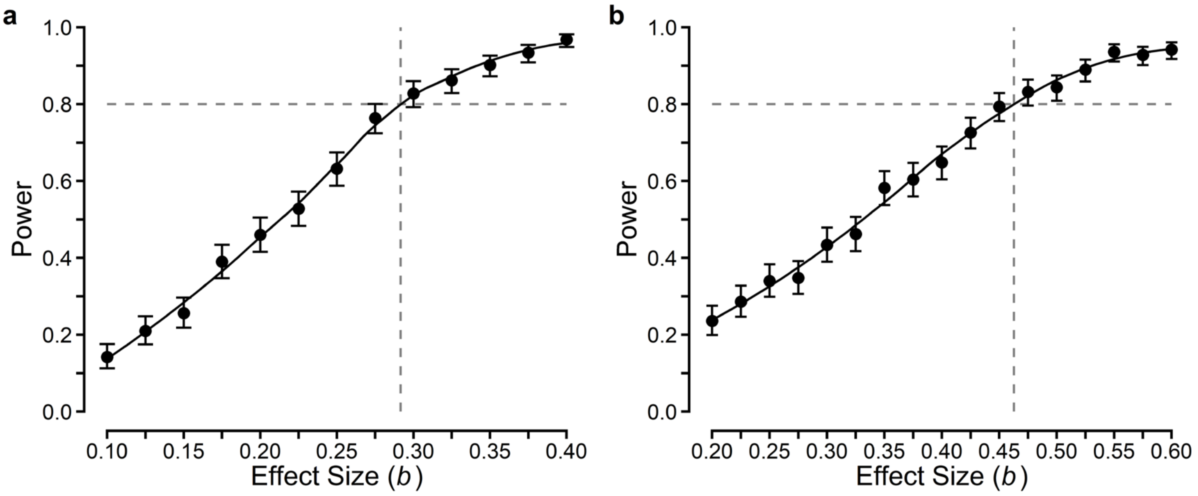


**Figure S36. Statistical power for a range of effect sizes. (a)** For the model of frequency of punishment in Experiment 2. **(b)** For the model of frequency of punishment in Experiment 3.

## 3. Supplementary References


1. van der Toorn, J., Ellemers, N. & Doosje, B. The threat of moral transgression: The impact of group membership and moral opportunity. *European Journal of Social Psychology* **45**, 609-622 (2015).
2. Doosje, B., Ellemers, N. & Spears, R. Perceived intragroup variability as a function of group status and identification. *Journal of Experimental Social Psychology* **31**, 410–436 (1995).
3. Murphy, R. O., Ackermann, K. A. & Handgraaf, M. Measuring social value orientation. *Judgment and Decision Making* **6**, 771–781 (2011).
4. Molenmaker, W. E., Lelieveld, G., de Kwaadsteniet, E. W. & van Dijk, E. Applying a logic of appropriateness to understand behavioral differences between common resource dilemmas and public good dilemmas. *Journal of Behavioral Decision Making* **35**, e2243 (2022).
5. Yamagishi, T. The provision of a sanctioning system as a public good. *Journal of Personality and Social Psychology* **51**, 110–116 (1986).
6. Mooijman, M., van Dijk, W. W., Ellemers, N. & van Dijk, E. Why leaders punish: A power perspective. *Journal of Personality and Social Psychology* **109**, 75–89 (2015).
7. Kuznetsova, A., Brockhoff, P. B. & Christensen, R. H. B. lmerTest package: Tests in linear mixed effects models. *Journal of Statistical Software* **82**, 1–26 (2017).
8. Raudenbush, S. W., Yang, M.-L. & Yosef, M. Maximum likelihood for generalized linear models with nested random effects via high-order, multivariate laplace approximation. *Journal of Computational and Graphical Statistics* **9**, 141–157 (2000).
9. Herrmann, B., Thöni, C. & Gächter, S. Antisocial punishment across societies. *Science* **319**, 1362–1367 (2008).
10. Green, P. & MacLeod, C. J. SIMR: an R package for power analysis of generalized linear mixed models by simulation. *Methods in Ecology and Evolution* **7**, 493–498 (2016).
